# The association between dietary protein intake and metabolic syndrome: a GRADE-assessed systematic review and meta-analysis of observational studies

**DOI:** 10.1186/s13098-025-02011-0

**Published:** 2025-12-29

**Authors:** Dorsa Ghazvineh, Ali Hosseinpour, Vahid Basirat, Elnaz Daneshzad

**Affiliations:** 1 Department of Exercise Physiology, K.C.*, Islamic Azad University of Karaj, Karaj, Iran; 2 Non-Communicable Diseases Research Center, Alborz University of Medical Sciences, Karaj, Iran; 3 Department of Gastroenterology, School of Medicine, Isfahan University of Medical Sciences and Health Services, Isfahan, Iran

**Keywords:** Animal proteins, Plant proteins, Metabolic syndrome, Systematic review, Meta-analysis

## Abstract

**Objectives:**

The primary aim of this meta-analysis is to assess the association of dietary protein with the risk of metabolic syndrome (MetS) in observational studies. In addition, the secondary aim is to evaluate the effectiveness of protein intake on MetS components.

**Methods:**

An Initial search was conducted from PubMed, Web of Science (WOS), and Scopus until May 2024. Cohort, cross-sectional, and case-control studies were included, and their quality and certainty were evaluated by the Newcastle – Ottawa Quality Assessment Scale (NOS) and the Grading of Recommendations, Assessment, Development, and Evaluation (GRADE) tools, respectively.

**Result:**

Based on our meta-analysis, we found that plant protein (PP), and animal protein (AP) had an inverse association with MetS (OR: 0.77, 95% CI: 0.69, 0.87, *P* < 0.001; I^2^ = 93.0%; P_heterogeneity_ < 0.001), (OR: 0.92, 95% CI: 0.86, 0.98, *P* = 0.012; I^2^ = 83.5%; P_heterogeneity_ < 0.001), respectively. Besides, there was no association between total protein (TP) and MetS (OR: 0.90, 95% CI: 0.82, 1.00, *P* < 0.051; I^2^ = 91.3%; P_heterogeneity_ < 0.001) as the primary outcomes. Furthermore, TP, AP, and PP had a negative association with MetS components, except TP-WC (OR: 0.78; 95% CI: 0.55, 1.12; *P* = 0.178; I^2^ = 80.0%; P_heterogeneity_ < 0.001), TP-FBS (OR: 0.93; 95% CI: 0.82, 1.05; *P* = 0.231; I^2^ = 91.0%; P_heterogeneity_ < 0.001), TP-BP (OR: 0.86; 95% CI: 0.76, 0.96; *P* = 0.008; I^2^ = 87.9%; P_heterogeneity_ < 0.001), AP-FBS (OR: 1.04, 95% CI: 1.00, 1.07, *P* = 0.061; I^2^ = 29.6%; P_heterogeneity_ >0.001), PP-FBS (OR: 0.94, 95% CI: 0.86, 1.03, *P* = 0.207; I^2^ = 72.2%; P_heterogeneity_ =0.001).

**Conclusion:**

Current evidence suggests that PP and AP intake may be associated with reduced risk of MetS as the primary outcome. However, in specific contexts, such as some of the secondary outcomes, results showed no reaction, e.g., TP-WC, TP-FBS, TP-BP, AP-FBS, PP-FBS. Besides, due to the high heterogeneity, methodological quality, and significant bias in PP-MetS and PP-TG, recommendations must be made cautiously. Finally, no definitive conclusions can be drawn regarding a causal or uniform protective relationship.

**Trial registration:**

Prospero ID 1020957.

**Supplementary Information:**

The online version contains supplementary material available at 10.1186/s13098-025-02011-0.

## Introduction

Chronic diseases are the most prevalent health disorders, including diabetes mellitus (DM), hypertension, and dyslipidemia, which can often increase the risk of mortality [1]. It can be prevented by managing risk factors, such as unhealthy eating habits (including overdrinking, overeating of saturated fats, added sugar, and ultra-processed foods), and obesity [2, 3].

Syndrome X or metabolic syndrome (MetS) implies a complex disorder that has become a global issue [4]. Six organizations set out an agreement for the diagnostic criteria of MetS in 2009, including the National Heart, Liver, and Blood Institute, the International Diabetes Federation (IDF), the American Heart Association (AHA), the World Heart Federation, the International Association for the Study of Obesity, and the International Atherosclerosis Society [5, 6]. Today, if a person presents at least three of the following items will be considered as an individual with MetS: (a) serum triglycerides (TG) ≥ 1.7 mmol/l (b) plummeted high-density lipoprotein cholesterol (HDL) (men < 1.03 mmol/l and women < 1.29 mmol/l) (c) blood pressure (BP) ≥ 130/85 mmHg or the use of antihypertension medication (d) raised fasting blood glucose (FBG) ≥ 100 mg/dl, and (e) waist circumference (WC) ≥ 102 cm for men and ≥ 88 cm for women [7, 8]. The outbreak of the MetS is increasing not only in the United States of America (USA) and Europe but also in Asian countries such as China, India, Iran, and South Korea [9]. There are many suggestions from specialists that a lifestyle shift in combination with dietary intervention has a considerable impact on people who suffer from MetS [10].

Protein, including Animal Protein (AP) (e.g., red meat, fish, and poultry), Plant Protein (PP) (e.g., plant-based diet), and Total Protein (TP) (e.g., AP and PP) [11, 12], comprises the majority of people’s daily energy intake. Notably, imbalanced or excessive macronutrient intake of specific types may lead to adverse metabolic outcomes in certain populations, particularly older adults [13, 14]. However, a normal amount of protein, whole-grain carbohydrates (carbs), and fiber can effectively control body weight (BW), diabetes, and pre-diabetes disorders [15, 16]. There is also a helpful impression of soy protein with isoflavones as a PP on serum lipids [10]. Mechanistically, protein can increase satiety, delay gastrointestinal emptying, and improve postprandial thermogenesis, which may contribute to decreased food intake, improved insulin sensitivity, and lipid profiles [17, 18].

According to the increasing global prevalence of MetS, we primarily aimed to understand how different dietary protein sources, such as TP, AP, and PP, affect MetS. Additionally, our secondary aim is to acquire evidence about the impact of protein intake on metabolic markers, including FBS, WC, BP, TG, and HDL. While previous studies have typically explored the relationship between protein intake and MetS, there remains a lack of comprehensive analyses comparing the specific impact of TP, AP, and PP on each MetS component. Besides, this study was carried out to fill the gaps by employing methods, such as the GRADE framework, to evaluate the accuracy of the association between causal factors, such as TP, AP, and PP consumption, on MetS and its components. Furthermore, our meta-analyses provide a more quantitative and detailed evaluation, offering new perspectives that may help healthcare providers, scientists, and policymakers to pursue more reliable dietary recommendations. The innovative aspect of this study consists of its focused comparison of protein sources and their potential impacts on MetS components. Additionally, our findings may complement previous research, which can be in favor of future studies aimed at personalized dietary interventions for those who are at risk of MetS.

## Methods

A systematic review and meta-analysis were conducted according to the Preferred Reporting Items for Systematic Reviews and Meta-Analyses (PRISMA), which assesses the relationship between dietary protein consumption and the MetS guidelines [19].

### Search strategy

Our initial search was conducted according to the main databases Scopus, PubMed, and Web of Science (WOS) until May 2024. The combination of text words and terms were applied in this investigation: (“protein“[tiab] OR “proteins“[tiab] OR “protein diet“[tiab] OR “protein-rich diets“[tiab] OR “total protein”[tiab] OR “protein intakes“[tiab] OR “dietary protein“[tiab] OR “Dietary Proteins“[MeSH] OR “animal protein“[tiab] OR “Animal Proteins“[MeSH] OR “vegetal protein“[tiab] OR “plant protein“[tiab] OR “Plant Proteins”[tiab] OR “Plant Proteins“[MeSH] OR “dairy protein“[tiab] OR “Grain Protein“[tiab] OR “Grain Proteins“[tiab] OR “Grain Proteins“[MeSH] OR “Fish Protein“[tiab] OR “Fish Proteins“[MeSH] OR “Meat Protein“[tiab] OR “Meat Proteins“[tiab] OR “Meat Proteins” [MeSH] OR “protein consumption“[tiab]) AND (“Metabolic Syndrome“[tiab] OR “Metabolic Syndrome“[MeSH] OR “syndrome X“[tiab] OR “Metabolic Syndrome X“[tiab] OR “Metabolic X Syndrome“[tiab]). Consequently, articles published before May 2024 were explored and imported into EndNote version 21. After removing duplicate studies, two authors screened the titles and abstracts to detect the related ones according to the purpose. In the next stage, all authors reviewed the remaining studies completely. Finally, we carefully reviewed bibliographies to decrease the likelihood of missing any publications.

### Inclusion and exclusion criteria

Observational studies that considered the association between dietary protein intake and MetS were included. Eligible criteria for inclusion were determined as follows:

- Any type of observational studies (cross-sectional, case-control, and cohort),
- Adult individuals (> 18 years old),
- Reported relative risk (RR), odds ratio (OR), hazard ratio (HR), and 95% confidence interval (CI).
- Articles which were published in English.

In addition, the following exclusion criteria were defined:

- Clinical trials, animal studies, and reviews.
- Lactating or pregnant women.
- Gray literature (books, letters, commentaries, and conferences).

### Data extraction

Two reviewers independently scanned the included publications for titles and abstracts. Also, they entered data from the final included studies into a datasheet in Microsoft Excel. Next, the OR and their 95% CI of MetS as primary outcome, and TG, HDL, BP, WC, and FBS, as secondary outcomes, were defined with TP, AP, and PP consumption outcomes added to the sheet. Besides, the first author’s name, publication date, sample size, design, population, age of participants, gender, study location, duration of observation, body mass index (BMI), protein assessment method, and MetS assessment method are shown in Table 1, separately. Protein intake categories (“high” vs. “low”) were extracted as defined in each study in a separate column for comparison. Additionally, most studies reported intake in quantiles (quartiles or tertiles), and we compared the highest vs. the lowest intake for the meta-analysis.

**Table 1:** Characteristics of included studies that investigated the relationship between dietary protein and metabolic syndrome

Authors year country Ref.	Design	Age (year)	BMI (kg/m^2^)	Gender	Sample size	Follow-up (Month)	Mets assessment method /Protein assessment method	Effect size OR (95%CI)	Outcomes	Comparison	Adjustment	NOS
Brunner et al. 2001 UK [30]	Cross-sectional	50.5±5.75	M: 25.2 ± 3.2 F: 25.4 ± 4.6	Both	6343 M:4480 F: 1863	24	Self-administered diet, questionnaire, and FFQ	TP M: 1.43 (1.13- 1.8) F: 1.13 (0.80- 1.62)	MetS*	T3 vs. T1	Age	8
Eilat-Adar et al. 2008 USA [22]	Cross-sectional	M:58.8±7.9 F:59.9±8.2	M: 28.9 ± 5.0 F: 31.1 ± 6.7	Both	1516 M:603 F: 913	24	ATP III and 24-hour dietary recalls	TP M: 0.7 (0.50–1.19.50.19) F: 1.36 (0.98–1.90.98.90) AP M: 0.91 (0.59–1.39.59.39) F: 1.36 (0.98–1.90.98.90) PP M: 0.68 (0.44–1.05.44.05) F: 1.06 (0.75–1.48.75.48)	MetS*	T3 vs. T1	Age, Smoking, Energy, Education, Study Center, Drinking	8
Gadgil et al. 2015 USA [23]	Cohort	53.8±9.35	26.4±5.04	Both	892	36	Health Assessment and Risk in Ethnic Groups: Food and FFQ	AP 4.26 (0.56–9.08.56.08)	WC, TG, ** HDL, LDL, TC, BP, FBS	T3 vs. T1	BMI, WC, Hb, HDL, LDL	8
Shang et al. 2017 Australia [32]	Cohort	49.2±7.1	24.1 ± 2.9	Both	5324	134	ATP III, and FFQ	TP 1.46 (1.01- 2.10) AP 1.67 (1.13- 2.48) PP 0.60 (0.37- 0.97)	MetS*	Q4 vs. Q1	Age, Smoking, PA, BMI, Sex, Percentage of energy, SFAs, MUFA, PUFA, Income, Drinking, WC, TFA, GI, BP, Vitamin C, and E, TC	8
Ahola et al. 2017 Finland [28]	Cross-sectional	50.5±12.8	26.6 ± 2.74	Both	791	1>	The self-reported questionnaire	TP M: 1.31 (0.76–2.36.76.36) F: 0.87 (0.49- 1.54)	WC, TG, ** HDL, BP	The highest Q vs. the lowest Q	Age, smoking, PA	8
Azemati et al. 2021 USA and Canada [29]	Cross-sectional	62.2±13.7	32.5 ± 6.6	Both	518	6	ATP III, and 24-h dietary recalls	TP 0.94 (0.89- 0.99) AP 0.94 (0.89–0.99.89.99) PP 0.94 (0.85–1.04.85.04)	MetS*	The highest Q vs. the lowest Q	Age, PA, BMI, Sex, Ethnicity, PUFA to SFAs ratio, Energy, Dietary Pattern, GL	7
Nabuco et al. 2018 Brazil [31]	Cross-sectional	67.5±5.2	30.2±1.95	F	245	-	ATP III, and 24-h dietary recalls	TP 3.10 (1.14–8.46.14.46)	WC, TG, ** HDL, BP, FBS	T3 vs. T1	SMM, Percent of BFM, Chronological Age	7
Kim et al. 2019 Korea [26]	Cross-sectional	48.7±0.3	23.7 ± 0.1	Both	7374	24	ATP III, and 24-h dietary recalls	TP 0.96 (0.63–1.46.63.46)	MetS*	Highest intake vs. lowest intake (Model 3 vs. unadjusted)	Daily Fiber, Carbohydrate, Protein, and Fat Intake	8
Chung et al. 2020 Korea [16]	Cohort	47±8.5	-	Both	13,485	60	ATP III, and 24-h dietary recalls	AP M: 1.28 (0.96–1.71.96.71) F: 0.81 (0.58–1.14.58.14) PP M: 0.90 (0.67–1.20.67.20) F: 0.92 (0.62–1.35.62.35)	WC, TG, ** HDL, BP, FBS	Q5 vs. Q1	Age, Smoking, PA, SFAs, MUFA, PUFA, Income, Education, Drinking	8
Park et al. 2021 Korea [27]	Case-control	M: 55±3.25 F: 57±2.75	M: 26±0.8 F:25.4±0.93	Both	130,423	108	ATP III and FFQ	TP M: 0.98 (0.94–1.02.94.02) F: 0.90 (0.87–0.94.87.94) AP M: 0.97 (0.94–1.0.94.0) F: 0.91 (0.89–0.93.89.93) PP M: 1.05 (1.01–1.1.01.1) F: 1.09 (1.05–1.13.05.13)	WC, TG, ** HDL, BP, FBS	Q3 vs. Q1	Age, Smoking, BMI, Energy, Income, Education, Drinking, Marital status, Occupation, Regular Exercise, Menopausal status	7
Vasbinder et al. 2021 USA [24]	Cohort	60<	32.2	F	3960	36	ATP III and FFQ	AP 1.01 (0.93–1.09) PP. 0.99 (0.94–1.05)	WC**	Highest Q vs. lowest Q	Age, Smoking, PA, Ethnicity, Income, Race, Medical History	8
Hajihashemi et al. 2021 Iran [33]	Cohort	35<	29.81 ± 4.02	Both	6504 M: 3168 F: 3336	138	Joint Scientific Statement, and FFQ	TP 0.83 (0.81–0.85.81.85) AP 0.8 (0.77–0.83.77.83) PP 0.7 (0.64–0.76.64.76)	MetS*	Multivariate adjusted vs. crude	Age, Smoking, PA, BMI, Fruits, Vegetables, Cereal, and Protein Sources	8
Jamshidi et al. 2022 Iran [34]	Cohort	M:48.95±9.6 F:48.72±9.57	M:24.07±4.35, F: 26.83±4.78	Both	M: 3168, F: 3336	55	ATP III, and FFQ	TP M: 0.24 (0.18–0.33), F: 0.42 (0.34–0.51), AP M: 0.50 (0.37–0.68), F: 0.88 (0.71–1.10), PP M: 0.35 (0.25–0.48), F: 0.41 (0.33–0.52)	WC, HC, ** WHR	Q5 vs. Q1	Age, PA wealth score, Smoking, MUFAs, PUFAs, SFAs, Sodium, Potassium, DM, Dyslipidemia, HTN	8
Lee et al. 2024 Korea [25]	Cross-sectional	70±2.5	-	Both	Urban: 1259 Rural: 462	24	ATP III, and 24-h dietary recalls	PP Urban: 0.23 (0.13–0.39) AP Urban: 0.86 (0.53–1.37) PP Rural: 0.59 (0.23–1.48) AP Rural: 0.57 (0.26–1.23)	WC, TG, ** HDL, BP, FBS	Q4 vs. Q1	Age, Sex, Income, Education level, Smoking, Alcohol Drinking, PA, Energy Intake	8

M: Male; F: Female; BMI: Body Mass Index; USA: United States America; AP: Animal Protein; PP: Plant Protein; TP: Total Protein; ATP III: National Cholesterol Education Program Adult Treatment III; FFQ: Food Frequency Questionnaire; MetS: Metabolic Syndrome; WC: Waist Circumference; GEE: The Generalized Estimating Equation; PA: Physical Activity; BP: Blood Pressure; WHR: Waist-to-Hip Ratio, HC: Hip Circumference, MUFA: Monounsaturated Fatty Acids, PUFA: Polyunsaturated Fatty Acids, SFAs: Saturated Fatty Acids, DM: Diabetes Mellitus, HTN: Hypertension, GL: Glycemic Load, TG: Triglycerides, HDL: High-Density Lipoprotein, LDL: Low-Density Lipoprotein, TC: Total Cholestrol, FBS: Fasting Blood Sugar, Hb: Hemoglobin, TFA: Trans Fatty Acid, GI: Glycemic Index, BFM: Body Fat Mass, SMM: Skeletal Muscle Mass, Q: Quartile, T: Tertile,

*: it refers to primary outcome, **: it refers to secondary outcome

### Quality assessment

Assessing the quality of studies was performed by the Newcastle-Ottawa Scale (NOS) [20], one of the most popular questionnaires for cross-sectional (Supplementary Table 1), cohort (Supplementary Table 2), and case-control (Supplementary Table 3) studies. Selection, outcome, and comparability are the main parts of this scale tool. A maximum of two, three, and four points was assigned to each section. By NOS thresholds, each numeric range has its description; for instance, 1–3 poor quality, 4–6 fair quality, and 7–9 high quality. The GRADE (Grading of Recommendations, Assessment, Development, and Evaluation) framework was employed to evaluate the certainty of the evidence, which assesses each outcome according to study limitations, consistency, directness, risk of publication bias, and precision.

### Statistical analysis

Studies that reported ratios and their 95% CI were used for the association between protein intake and MetS. Only observational studies were eligible for inclusion, as no randomized controlled trials on the topic were available at the time of the review. Egger’s weighted regression test was used to assess the presence of small-study effects as an indicator of potential publication bias. Log ORs, standard errors (SEs) using the ORs, and their 95% CI were considered for all data. A random-effects model was used to pool effect sizes. To discover the sources of heterogeneity, the Q Cochrane test and I^2^ statistics were used, as described by Higgins and Thompson: a statistic of I^2^ >50% indicated heterogeneity by the scores of 50% or higher [21]. Moreover, subgroup analysis used for the following factors: study type, gender (men, women, or both), countries which finally are presented as continents (USA, United Kingdom (UK), Australia, Korea, Finland, Brazil, and Iran), protein assessment tool (24-hour dietary recall method, food frequency questionnaire (FFQ)), and finally if the included studies adjusted or not adjusted the ratios for confounders (age, physical activity (PA), smoking, and BMI). A funnel plot was also applied to identify any publication bias. Moreover, Egger’s weighted regression was employed to address statistical issues. To detect publications that affect the overall effect size, sensitivity analysis was applied to remove particular studies, and finally, STATA software was used for statistical analysis (version 14.0 (Stata Corp LP, College Station, TX)).

## Results

### Study selection

**Fig. 1:**
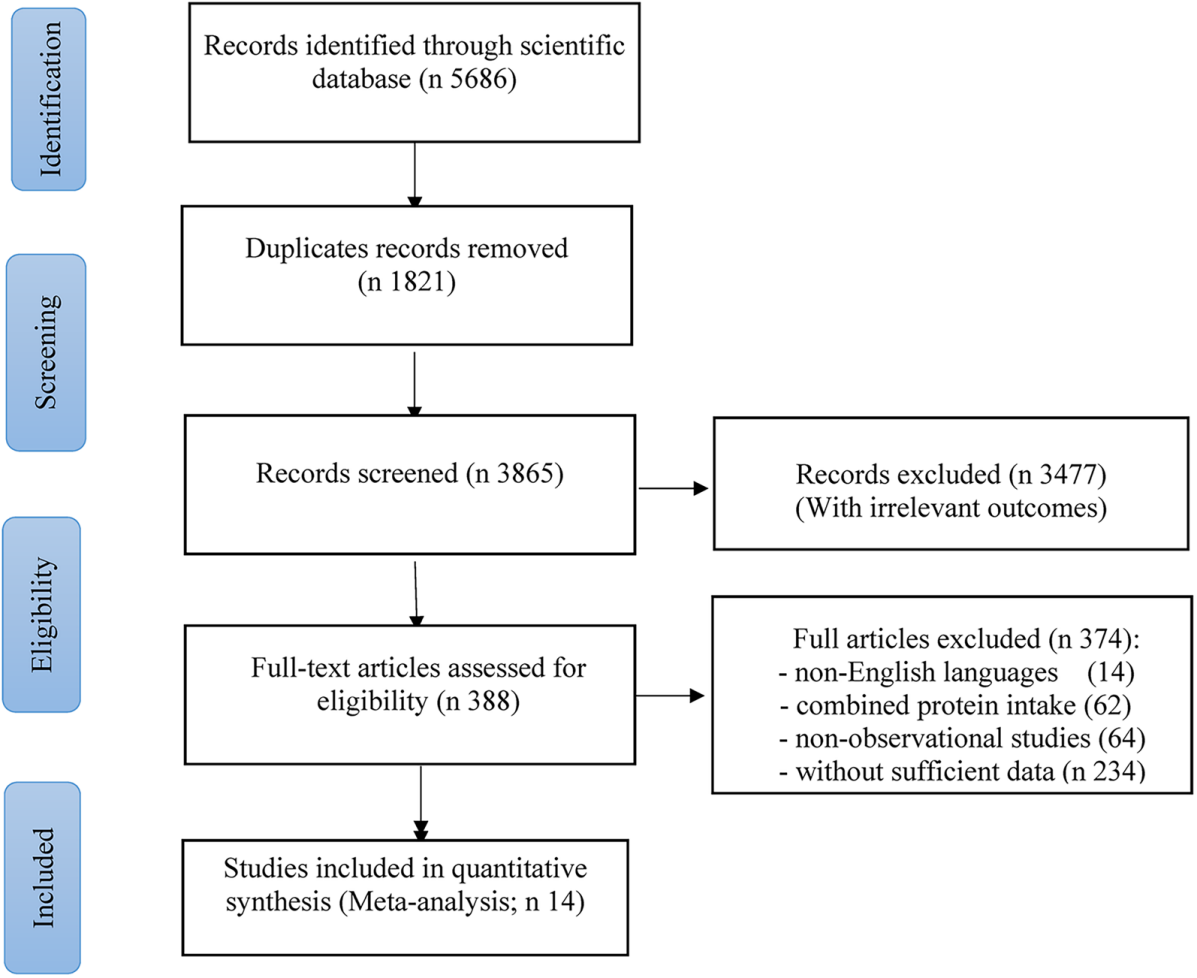
Flow diagram showing the selection of observational studies for the current systematic review and meta-analysis of the association between dietary protein intake and MetS

Figure 1 shows a PRISMA diagram summarizing data screening and inclusion. 5686 publications were identified via screening of records through the databases PubMed: 2446, Scopus: 2345, and Web of Science: 1130. After eliminating 1821 duplicates and 3477 articles by title and abstract, 388 remained and were subjected to full-text screening. Finally, fourteen studies were eligible to be included in our meta-analysis.

### Basic characteristics of the selected studies

The basic characteristics of the final fourteen included studies can be found in Table 1. The included studies were published between 2001 and 2024, with 185,564 participants aged between 30 and 75 years and older. Also, all studies reported two or more MetS component results, such as WC, BP, low-density lipoprotein (LDL), HDL, FBG, and total cholesterol (TC). Three studies were conducted in the USA [22–24], and Four in Korea [16, 25–27]. However, the remaining were performed in Finland [28], the USA and Canada [29], the United Kingdom (UK) [30], Brazil [31], Australia [32], and two in Iran [33, 34]. Seven studies were cross-sectional [22], [25], [26], [28–31], six were cohort studies [16, 23, 24, 32–34], and one was case-control [27]. All studies examined both genders, except two in women [24, 31]. FFQ was employed in seven papers [23, 24, 27, 30, 32–34], while 24-hour food recall was applied in six studies [16, 22, 25, 26, 29, 31]. Ten studies evaluated MetS by the National Cholesterol Education Program Adult Treatment Panel III, the 2009 Joint Scientific Statement (ATP III) [16, 22, 24–27, 29, 31, 32, 34], and the rest of the studies used other types of evaluating MetS [23, 28, 30].

### Protein intake and MetS

Table 2 consists of supplementary data for evaluating the effect of proteins on MetS among observational studies in our research and was considered part of the systematic review. For instance, Azemati et al.‘s study reported that WC had a positive association with TP (β = 0.004, 95%CI: 0.002, 0.006) and AP intakes (β < 0.001, 95%CI: 0.001, 0.007). Also, fasting blood sugar (FBS) was strongly associated with the AP ratio (β = 0.023, 95%CI: 0.005, 0.041). However, HDL, TG, systolic, and diastolic blood pressure (SBP and DBP) showed no significant relationship with dietary protein intake [29].

**Table 2:** Summary of observational studies on protein intake and metabolic syndrome components

Study Ref.	Design	Population	Exposure	Outcome(s)	Effect size/Result	Key findings
Brunner et al. [30]	Cross-sectional	British Men	TC, TP	MetS*	Not quantified	TC and TP intake are associated with increased risk of MetS in men.
Eilat-Adar et al. [22]	Cross-sectional	Israeli Men	Percent of Energy from PP, AP, TP	MetS*	Not quantified	Higher PP intake is associated with a lower prevalence of MetS signs in men.
Gadgil et al. [23]	Cohort	Not specified	AP	TC, LDL**	AP is not significantly associated with TC/LDL after adjusting for BMI, lifestyle, etc.	The fully adjusted model shows no association between AP and lipid profile.
Shang et al. [32]	Cohort	Not specified	AP, PP, TP	WC, SBP, ** BW, TC	AP (per +5%): ↑1.25 cm on WC, ↑1.05 mmHg on SBP, ↑0.94 kg on BW TP: ↑1.17 cm on WC, ↑0.97 mmHg on SBP, ↑0.78 kg on BW PP: ↓1.73 cm on WC, ↓1.97 kg on BW; no effect on TC	AP and TP intake are associated with increases in WC, BP, and weight; PP intake is associated with reductions.
Ahola et al. [28]	Cross-sectional	Not specified	Replacing Carbs/ Fats with Protein	SBP**	Protein substitution is associated with lower SBP	Replacing carbs/fats with protein lowered SBP.
Azemati et al. [29]	Cross-sectional	Iranian Adults	TP, AP, AP ratio	WC, FBS, ** HDL, TG, SBP, DBP	WC: TP (β = 0.004), AP (β < 0.001); FBS: AP ratio (β = 0.023); no significant association with other outcomes	TP and AP are positively associated with WC and FBS. Besides, there was no significant effect on other components.
Nabuco et al. [31]	Cross-sectional	Brazilian Women	TP, Carbs, Fats	MetS*	Not quantified	Women with MetS consumed less protein and more carbs/fats.
Kim et al. [26]	Cross-sectional	Korean Women with MHO	TP	MHO**	OR = 5.85 (95% CI: 1.13–30.31)	Low protein intake is significantly associated with MHO in females.
Chung et al. [16]	Cohort	Korean Adults	AP, PP	WC, FBG**	OR (WC, men) = 1.30; OR (FBG, men) = 1.32; no significant association in women	AP is associated with increased WC and FBG in men only.
Park et al. [27]	Case-control	Older Adults	AP, PP	HDL, BP, ** hyperglycemia	Not quantified	Low AP and high PP are associated with lower HDL, higher BP; hyperglycemia is more common in males.
Vasbinder et al. [24]	Cohort	Dutch adults	AP, PP	BW status, **	AP positively associated with MetS (95% CI: 1.02–1.14); no significant effect for PP (95% CI: 0.95–1.03)	AP increased MetS risk; no association for PP.
Hajihashemi et al. [33]	Cohort	Iranian adults	AP, PP, TP	MetS*	AP: OR = 0.80; TP: OR = 0.83; PP: OR = 0.70	All protein types are inversely associated with MetS risk.
Jamshidi et al. [34]	Cohort	Iranian Adults	AP, PP, TP	Abdominal** Obesity, BP, TG, HDL, and BFM	PP reduced all MetS components; AP reduced abdominal obesity (both sexes), SBP, DBP, TG, MetS in males; TP ↓ FM by 7.1 kg (men), 4.5 kg (women)	PP had broad benefits; AP and TP reduced fat and MetS components, particularly in males.
Lee et al. [25]	Cross-sectional	Rural/urban older adults (Korea)	AP, PP	Abdominal** obesity, TG, HDL, MetS	Urban: PP reduced abdominal obesity, TG, MetS; ↑HDL AP reduced obesity but had a limited effect on HDL	PP is more beneficial in urban adults; AP reduced obesity but had a less overall effect.

NS: Not Significant, WC: Waist Circumference, FBS: Fasting Blood Sugar, FBG: Fasting Blood Glocuse, BP: Blood Pressure, SBP/DBP: Systolic/Diastolic Blood Pressure, HDL: High-Density Lipoprotein, TG: Triglycerides, TC: Total Cholesterol, LDL: Low-Density Lipoprotein, AP: Animal Protein, PP: Plant Protein, TP: Total Protein, MetS: Metabolic Syndrome, MHO: Metabolically Healthy Obesity, BW: Body Weight, BFM: Body Fat Mass, Carbs: Carbohydrates, BMI: Body Mass Index

*: it refers to primary outcome, **: it refers to secondary outcome

### Meta-analysis

This review evaluates the association between dietary protein intake and MetS and its components among 14 publications, with 185,564 cases.

## Primary outcomes

### TP and MetS

**Fig. 2:**
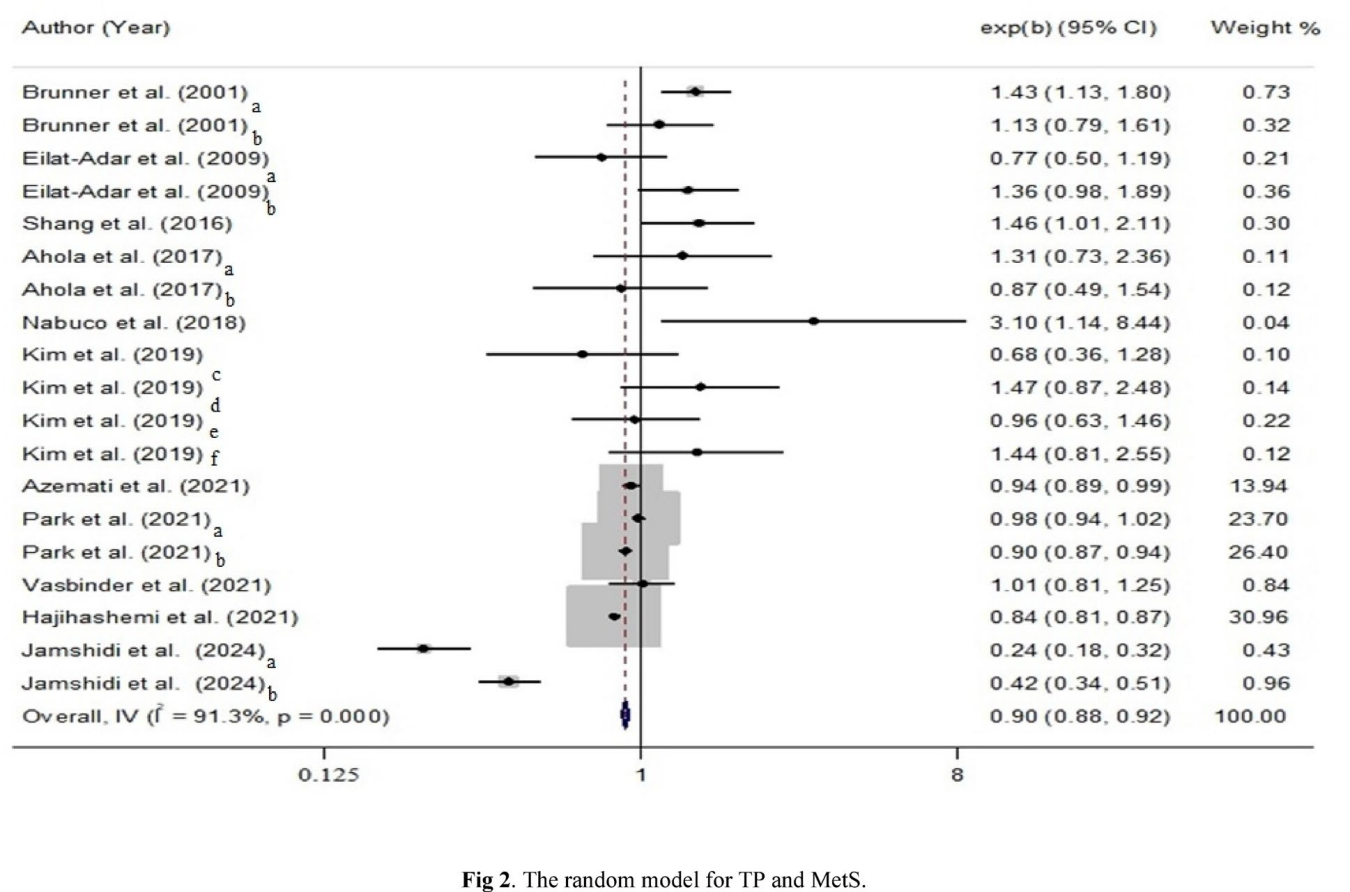
The random model for TP and MetS In this analysis a to f represents the effect sizes according different variables including as follows: a: males, b: females, c: males metabolically abnormal but of normal weight (MANW), d: female MANW, e: males metabolically abnormal and obese (MAO), f: females MAO

There was a non-significant trend toward an inverse association between TP and MetS (OR: 0.90, 95% CI: 0.82, 1.00, *P* < 0.051; I^2^ = 91.3%; P_heterogeneity_ < 0.001) (Fig. 2). According to subgroup analysis, the continent, gender, MET assessment, and protein assessment methods were the sources of heterogeneity. Also, the studies adjusted for age and BMI were the source of heterogeneity (Supplementary Table 4). We found asymmetry by visually examining the funnel plot (Supplementary Fig. 1); however, by conducting Egger’s regression test (*P* = 0.832), no considerable publication bias was seen. Notably, following the sensitivity analysis, the overall effect size did not depend on any study (OR: 0.90, CI: 0.82, 1.00) (Supplementary Fig. 2).

### AP and MetS

**Fig. 3:**
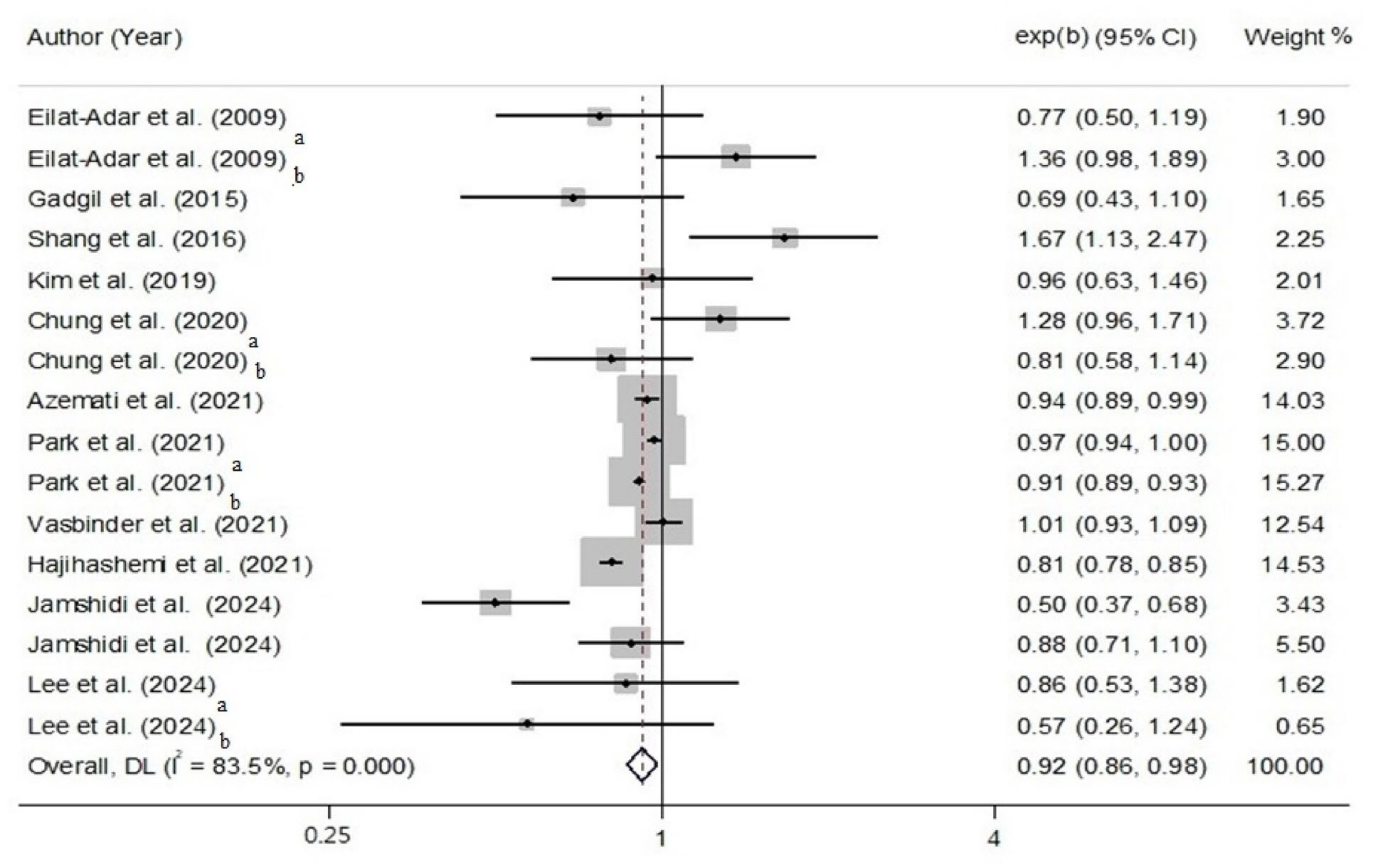
The random model for AP and MetS In this analysis, a and b represent the effect sizes according to different genders, as follows: a: males, b: females

Based on meta-analysis, AP negatively correlated with MetS (OR: 0.92, 95% CI: 0.86, 0.98, *P* = 0.012; I^2^ = 83.5%; P_heterogeneity_ < 0.001) (Fig. 3). According to subgroup analysis, continent, study design, protein assessment, and MetS assessment made heterogeneity. Notably, BMI and smoking adjustments made heterogeneity (Supplementary Table 5). We found no asymmetry by visually testing the funnel plot (Supplementary Fig. 3). Besides, by conducting Egger’s regression test (*P* = 0.991), no significant publication bias was found. Sensitivity analysis also showed that the overall effect size regarding the association between AP and MetS did not depend on a single study (OR: 0.92, CI: 0.86, 0.98) (Supplementary Fig. 4).

### PP and MetS

**Fig. 4:**
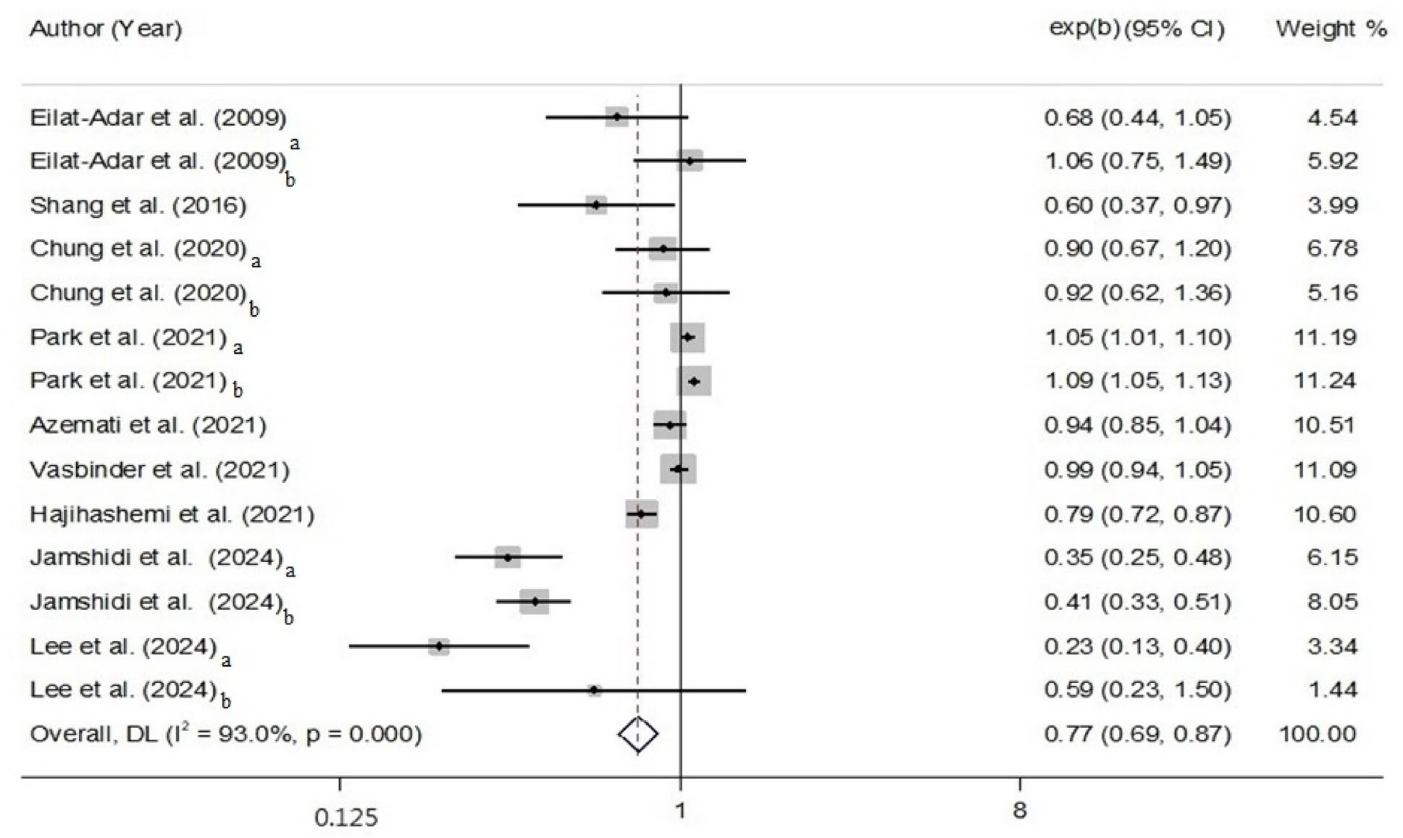
The random model for PP and MetS In this analysis, a and b represent the effect sizes according to different genders, as follows: a: males, b: females

PP had an inverse association with MetS according to the meta-analysis (OR: 0.77, 95% CI: 0.69, 0.87, *P* < 0.001; I^2^ = 93.0%; P_heterogeneity_ < 0.001) (Fig. 4). The subgroup analysis demonstrated that the continent and the MetS assessment caused heterogeneity. Notably, smoking and PA adjustments made heterogeneity (Supplementary Table 6). We found asymmetry by visually examining the funnel plot (Supplementary Fig. 5). Moreover, Egger’s regression test showed publication bias (*P* = 0.004); therefore, interpreting the results must be done cautiously. However, following sensitivity analysis, the overall effect size did not depend on a study (OR: 0.77, CI: 0.69, 0.87) (Supplementary Fig. 6).

## Secondary outcomes

### TP and MetS components

#### TP and TG

TP was inversely correlated to TG (OR: 0.80, 95% CI: 0.70, 0.91, *P* < 0.001; I^2^ = 89.5%; P_heterogeneity_ < 0.001) (Supplementary Fig. 7). In addition, there were some sources of heterogeneity in the subgroup, such as continent, protein assessment, and MetS assessment. Regarding adjustments, BMI and smoking were heterogeneous (Supplementary Table 7). By conducting a visual examination of the funnel plot, we found no asymmetry (Supplementary Fig. 8). Additionally, by conducting Egger’s regression test (*P* = 0.336), no considerable publication bias was seen. Additionally, sensitivity analysis revealed that the overall effect size regarding the association between TP and TG was not dependent on a single study (OR: 0.80, CI: 0.70, 0.91) (Supplementary Fig. 9).

#### TP and HDL

There was a negative correlation between TP and HDL (OR: 0.80, 95% CI: 0.72, 0.89, *P* < 0.001; I^2^ = 85.6%; P_heterogeneity_ < 0.001) models (Supplementary Fig. 10). Based on the subgroup analysis, continent, study design, and protein assessment were the sources of heterogeneity. According to the adjustments, BMI and smoking caused heterogeneity (Supplementary Table 8). By the visual inspection of the funnel plot, we found a symmetry (Supplementary Fig. 11). Additionally, by conducting Egger’s regression test (*P* = 0.394), no significant publication bias was seen. Sensitivity analysis also showed that the overall effect size regarding the association between TP and HDL did not depend on a single study (OR: 0.80, CI: 0.72, 0.89) (Supplementary Fig. 12).

#### TP and WC

There was no significant association between TP and WC (OR: 0.78; 95% CI: 0.55, 1.12; *P* = 0.178; I^2^ = 80.0%; P_heterogeneity_ < 0.001) (Supplementary Fig. 13). Furthermore, continent, study designs, protein assessment, and MetS assessment made heterogeneity. Additionally, BMI, smoking, and PA adjustments were the sources of heterogeneity (Supplementary Table 9). Following the visual inspection of the funnel plot, we found an asymmetry (Supplementary Fig. 14); however, by conducting Egger’s regression test (*P* = 0.279), no substantial publication bias was seen. Sensitivity analysis also showed that the overall effect size regarding the association between TP and WC did not depend on a single study (OR: 0.78, CI: 0.55, 1.12) (Supplementary Fig. 15).

#### TP and FBS

There was no considerable correlation between TP and FBS (OR: 0.93; 95% CI: 0.82, 1.05; *P* = 0.231; I^2^ = 91.0%; P_heterogeneity_ < 0.001) (Supplementary Fig. 16). Continent, study designs, and protein assessment caused heterogeneity. Regarding adjustments, BMI, smoking, and PA were the potential sources (Supplementary Table 10). Besides, by visual inspection of the funnel plot, we found no asymmetry (Supplementary Fig. 17); additionally, Egger’s regression test (*P* = 0.422) showed no significant publication bias. Sensitivity analysis also revealed that the overall effect size regarding the association between TP and FBS did not depend on a single study (OR: 0.93, CI: 0.82, 1.05) (Supplementary Fig. 18).

#### TP and BP

TP and BP had no substantial association (OR: 0.86; 95% CI: 0.76, 0.96; *P* = 0.008; I^2^ = 87.9%; P_heterogeneity_ < 0.001) (Supplementary Fig. 19). Besides, in the subgroup analysis, continent, study design, protein assessment, and MetS assessment were considered. In terms of sources of heterogeneity in adjustment groups, BMI, smoking, and PA (Supplementary Table 11). Following the visual inspection, there was a symmetry in the funnel plot (Supplementary Fig. 20). Additionally, by conducting Egger’s regression test (*P* = 0.368), no substantial publication bias was seen. Sensitivity analysis also showed that the overall effect size regarding the association between TP and BP did not depend on a single study (OR: 0.86, CI: 0.76, 0.96) (Supplementary Fig. 21).

### AP and MetS components

#### AP and TG

There was an inverse association between AP and TG (OR: 0.92, 95% CI: 0.87, 0.97, *P* = 0.003; I^2^ = 64.4%; P_heterogeneity_ >0.001) (Supplementary Fig. 22). Subgroup analysis showed that gender, protein assessment, and study designs were the sources of heterogeneity. Besides, BMI and PA adjustments were the sources of heterogeneity (Supplementary Table 12). On the other hand, testing the funnel plot visually showed asymmetry (Supplementary Fig. 23). Besides, no significant publication bias was found following Egger’s regression test (*P* = 0.653). In addition, sensitivity analysis showed that the overall effect size regarding the association between AP and TG did not depend on a single study (OR: 0.92, CI: 0.87, 0.97) (Supplementary Fig. 24).

#### AP and HDL

There was an inverse association between AP and HDL (OR: 0.90, 95% CI: 0.85, 0.95, *P* < 0.000; I^2^ = 60.0%; P_heterogeneity_ >0.001) (Supplementary Fig. 25). Regarding subgroup analysis, study design, protein assessment, and gender contributed to heterogeneity. Furthermore, PA and BMI adjustments were considered as the sources of heterogeneity (Supplementary Table 13). Furthermore, testing the funnel plot visually showed asymmetry (Supplementary Fig. 26). Besides, by conducting Egger’s regression test (*P* = 0.767), no considerable publication bias was seen. In addition, sensitivity analysis showed that the overall effect size regarding the association between AP and HDL did not depend on a single study (OR: 0.90, CI: 0.85, 0.95) (Supplementary Fig. 27).

#### AP and WC

There was an inverse association between AP and WC (OR: 0.78, 95% CI: 0.68, 0.90, *P* = 0.001; I^2^ = 94.0%; P_heterogeneity_ < 0.001) (Supplementary Fig. 28). Additionally, the sources of heterogeneity were study design and gender. PA adjustment also made heterogeneity (Supplementary Table 14). Furthermore, an asymmetry was seen after visually testing the funnel plot (Supplementary Fig. 29). Besides, Egger’s regression test showed no significant publication bias (*P* = 0.112). In addition, sensitivity analysis depicted that the overall effect size regarding the association between AP and WC depended on the study of Jamshidi et al. [34]. By excluding that study, a significant association was found between AP and WC (OR: 0.94, CI: 0.86, 1.03) (Supplementary Fig. 30).

#### AP and FBS

The association between AP and FBS was not significant (OR: 1.04, 95% CI: 1.00, 1.07, *P* = 0.061; I^2^ = 29.6%; P_heterogeneity_ >0.001) (Supplementary Fig. 31). Moreover, there were some sources of heterogeneity, including study design, gender, and protein assessment. Furthermore, PA and BMI adjustments were the sources of heterogeneity (Supplementary Table 15). Almost no asymmetry was seen after visually testing the funnel plot (Supplementary Fig. 32). Besides, Egger’s regression test showed no significant publication bias (*P* = 0.536). Additionally, sensitivity analysis illustrated that the overall effect size regarding the association between AP and FBS did not depend on a single study (OR: 1.04, CI: 1.00, 1.07) (Supplementary Fig. 33).

#### AP and BP

The association between AP and BP was negative (OR: 0.96, 95% CI: 0.95, 0.98, *P* < 0.001; I^2^ = 0.0%; P_heterogeneity_ >0.001) (Supplementary Fig. 34). Besides, protein assessment was the source of heterogeneity (Supplementary Table 16). Notably, no asymmetry was found after visually examining the funnel plot (Supplementary Fig. 35). Besides, Egger’s regression test showed no significant publication bias (*P* = 0.170). Furthermore, based on sensitivity analysis, the overall effect size did not depend on a study (OR: 0.96, CI: 0.95, 0.98) (Supplementary Fig. 36).

### PP and MetS components

#### PP and TG

PP was inversely correlated to TG according to the meta-analysis (OR: 0.85, 95% CI: 0.74, 0.96, *P* = 0.012; I^2^ = 89.2%; P_heterogeneity_ < 0.001) (Supplementary Fig. 37). Moreover, continents, study designs, and protein assessment were the sources of heterogeneity. Notably, PA and BMI adjustments made heterogeneity (Supplementary Table 17). We identified asymmetry by visually examining the funnel plot (Supplementary Fig. 38). Additionally, Egger’s regression test revealed publication bias (*P* = 0.004); therefore, interpreting the results must be done cautiously. Furthermore, following sensitivity analysis, the overall effect size had no dependency on a study (CI: 0.74, 0.96) (Supplementary Fig. 39).

#### PP and HDL

PP was inversely correlated to HDL according to the meta-analysis (OR: 0.83, 95% CI: 0.72, 0.95, *P* = 0.007; I^2^ = 90.9%; P_heterogeneity_ < 0.001) (Supplementary Fig. 40). According to the subgroup analysis, the study design was the source of heterogeneity. Furthermore, PA adjustment caused heterogeneity (Supplementary Table 18). Besides, we found asymmetry after visually examining the funnel plot (Supplementary Fig. 41). There was no publication bias following Egger’s regression test (*P* = 0.076). Additionally, sensitivity analysis showed that the overall effect size regarding the association between PP and HDL did not depend on a single study (OR: 0.83, CI: 0.72, 0.95) (Supplementary Fig. 42).

#### PP and WC

The relationship between PP and WC was negative according to the meta-analysis (OR: 0.34, 95% CI: 0.14, 0.80, *P* = 0.014; I^2^ = 99.9%; P_heterogeneity_ < 0.001) (Supplementary Fig. 43). According to the subgroup analysis, the study design and PA adjustment were the sources of heterogeneity (Supplementary Table 19). Additionally, we observed asymmetry upon visually examining the funnel plot (Supplementary Fig. 44). No publication bias was detected using Egger’s regression test (*P* = 0.642). Additionally, sensitivity analysis showed that the overall effect size regarding the association between PP and WC did not depend on a single study (OR: 0.33, CI: 0.14, 0.80) (Supplementary Fig. 45).

#### PP and FBS

There was no substantial correlation between PP and FBS following the meta-analysis (OR: 0.94, 95% CI: 0.86, 1.03, *P* = 0.207; I^2^ = 72.2%; P_heterogeneity_ =0.001) (Supplementary Fig. 46). In addition, by subgroup analysis, gender, protein assessment, and study design were sources of heterogeneity. Moreover, BMI and PA adjustments caused heterogeneity (Supplementary Table 20). An asymmetry was found after visually examining the funnel plot (Supplementary Fig. 47). Also, there was no publication bias following Egger’s regression test (*P* = 0.051). Additionally, sensitivity analysis showed that the overall effect size depended on the study of Park et al. [27] (Females: OR: 0.84, CI: 0.68, 1.03, and males: OR: 0.83, CI: 0.68, 1.02). Excluding that study, a significant association was found between PP and FBS (Supplementary Fig. 48).

#### PP and BP

PP and BP showed a negative association following the meta-analysis (OR: 0.91, 95% CI: 0.82, 1.01, *P* = 0.090; I^2^ = 83.1%; P_heterogeneity_ <0.001) (Supplementary Fig. 49). Based on subgroup analysis, gender, study design, and protein assessment were the sources of heterogeneity. Besides, BMI and PA adjustments caused heterogeneity (Supplementary Table 21). Finally, an asymmetry was seen after visually examining the funnel plot (Supplementary Fig. 50). Also, there was no publication bias following Egger’s regression test (*P* = 0.034). Additionally, sensitivity analysis showed that the overall effect size depended on the study of Park et al. [27], such that by excluding that study, a significant association was found between PP and BP (OR: 0.81, CI: 0.64, 1.02) (Supplementary Fig. 51).

### Quality assessment

Based on the NOS quality assessment, three studies ranked 7 [27, 29, 31] and eleven scored 8 [16, 22–26, 28, 30, 32–34].

### Grade assessment

The GRADE profile for the impact of dietary protein intake on MetS and its components, either regarding its incidence or its prevalence, is presented in Table 3. The quality of evidence for most outcomes (MetS, WC, BP, HDL, TG, and FBS) was graded as very low.

**Table 3:** GRADE assessment of dietary protein intake and the risk of metabolic syndrome

**Type of protein**	**Exposure**	**Number of studies**	**Risk of Bias**	**Inconsistency**	**Indirectness**	**Imprecision**	**Publication bias**	**Number**	**Relative (95% CI)**	**Certainty**
**Primary outcomes**										
AP	MetS	16	not serious	very serious^f^	not serious	not serious	None	178221	0.92 (0.86, 0.98)	⨁◯◯◯Very low
PP	MetS	14	not serious	very serious^f^	serious^b^	not serious	publication bias strongly suspected^e^	169955	0.77 (0.69 to 0.87)	⨁◯◯◯Very low
TP	MetS	19	not serious	very serious^f^	not serious	serious^d^	None	190833	0.90 (0.82 to 1.00)	⨁◯◯◯Very low
**Secondary outcomes**										
AP	BP	9	not serious	not serious	very serious^b^	not serious	None	153025	0.96 (0.95 to 0.98)	⨁◯◯◯Very low
AP	FBS	9	not serious	not serious	very serious^b^	serious^d^	None	153025	1.04 (1.00 to 1.07)	⨁◯◯◯Very low
AP	HDL	8	not serious	Serious^f^	very serious^b^	not serious	None	152133	0.90 (0.85 to 0.95)	⨁◯◯◯Very low
AP	TG	8	not serious	very serious^f^	very serious^b^	not serious	None	152133	0.92 (0.87 to 0.97)	⨁◯◯◯Very low
AP	WC	8	not serious	very serious^f^	very serious^b^	not serious	None	152133	0.78 (0.55 to 1.12)	⨁◯◯◯Very low
PP	BP	8	not serious	very serious^f^	very serious^b^	serious^d^	publication bias strongly suspected^e^	152133	0.91 (0.82 to 1.01)	⨁◯◯◯Very low
PP	FBS	8	not serious	serious^f^	very serious^b^	serious^d^	None	152133	0.94 (0.86 to 1.03)	⨁◯◯◯Very low
PP	HDL	8	not serious	very serious^f^	very serious^b^	not serious	None	152133	0.83 (0.72 to 0.95)	⨁◯◯◯Very low
PP	TG	8	not serious	very serious^f^	very serious^b^	not serious	publication bias strongly suspected^e^	152133	0.85 (0.74 to 0.96)	⨁◯◯◯Very low
PP	WC	8	not serious	very serious^f^	very serious^b^	not serious	None	152133	0.34 (0.14 to 0.80)	⨁◯◯◯Very low
TP	BP	7	not serious	very serious^f^	serious^b^	not serious	None	137172	0.86 (0.76 to 0.96)	⨁◯◯◯Very low
TP	FBS	5	not serious	very serious^f^	very serious^b^	serious	None	137172	0.93 (0.82 to 1.05)	⨁◯◯◯Very low
TP	HDL	7	not serious	very serious^f^	very serious^b^	not serious	None	137172	0.80 (0.72 to 0.89)	⨁◯◯◯Very low
TP	TG	7	not serious	very serious^f^	very serious^b^	not serious	None	137172	0.80 (0.70 to 0.91)	⨁◯◯◯Very low
TP	WC	7	not serious	very serious^f^	serious^b^	serious^d^	None	137172	0.78 (0.55 to 1.12)	⨁◯◯◯Very low

AP: Animal Protein, PP: Plant Protein, TP: Total Protein, MetS: Metabolic Syndrome, CI: confidence interval, OR: odds ratio, WC: Waist Circumference, FBS: Fasting Blood Sugar, BP: Blood Pressure, HDL: High-Density Lipoprotein, TG: Triglycerides

^a^: Downgraded since more than 50% of the participants were from high-risk of-bias studies

^b^: Downgraded for indirectness in the country

^c^: Downgraded since the participants included were fewer than 400 persons

^d^: Downgraded since the 95% CI crosses the threshold of interest

^e^: Publication Bias was detected through Egger and Begg's test. (p-value < 0.05)

^f^: The I2 value was >50% (or Heterogeneity among the studies was high)

## Discussion

### Summary of findings

This systematic review and meta-analysis focused on the effect of dietary protein intake on MetS and its components as the primary and secondary outcomes, respectively. Firstly and most importantly, protein is categorized as AP (e.g., red meat, fish, and poultry), PP (e.g., plant-based diet), and TP (e.g., AP and PP) [11, 12]. Based on our meta-analysis, we found that PP, and AP had an inverse association with MetS (OR: 0.77, 95% CI: 0.69, 0.87, *P* < 0.001; I^2^ = 93.0%; P_heterogeneity_ < 0.001), (OR: 0.92, 95% CI: 0.86, 0.98, *P* = 0.012; I^2^ = 83.5%; P_heterogeneity_ < 0.001), respectively. Besides, there was no association between TP and MetS (OR: 0.90, 95% CI: 0.82, 1.00, *P* < 0.051; I^2^ = 91.3%; P_heterogeneity_ < 0.001) as the primary outcomes. Furthermore, TP, AP, and PP had a negative association with MetS components, except TP-WC (OR: 0.78; 95% CI: 0.55, 1.12; *P* = 0.178; I^2^ = 80.0%; P_heterogeneity_ < 0.001), TP-FBS (OR: 0.93; 95% CI: 0.82, 1.05; *P* = 0.231; I^2^ = 91.0%; P_heterogeneity_ < 0.001), TP-BP (OR: 0.86; 95% CI: 0.76, 0.96; *P* = 0.008; I^2^ = 87.9%; P_heterogeneity_ < 0.001), AP-FBS (OR: 1.04, 95% CI: 1.00, 1.07, *P* = 0.061; I^2^ = 29.6%; P_heterogeneity_ >0.001), PP-FBS (OR: 0.94, 95% CI: 0.86, 1.03, *P* = 0.207; I^2^ = 72.2%; P_heterogeneity_ =0.001).

### Findings concerning the literature and mechanism

In line with our findings, some publications reported the effectiveness of dietary protein intake on MetS and its components. For example, Mohammadifard et al. showed the effect of soy protein can decrease TG (WMD: −0.29; 95% CI: −0.49, −0.09 mg/dL), TC (WMD: −1.46; 95% CI: −1.70, −1.22 mg/dL), LDL (WMD: −0.73; 95% CI: −0.93, −0.52 mg/dL), FBS (WMD: −0.90; 95% CI: −1.12, −0.68 mg/dL), and insulin (WMD: −1.06; 95% CI: −1.29, −0.84 pmol/L); but there was no change in serum HDL and BP levels [42]. Badely et al. reviewed the effect of whey protein supplementation on MetS components in overweight and obese individuals, which significantly reduced the SBP, DBP, HDL, WC, TG, and FBS in intervention groups in comparison to control groups (−7.46, 95% CI: −9.39,−6.13), (−5.68, 95% CI: −6.69,−4.67), (−6.07, 95% CI: −7.53,−4.61), (−2.76, 95% CI: −3.83,−1.69), (−18.19, 95% CI: −22.49,−15.30), and (−1.42, 95% CI: −1.52,−1.31), *P* < 0.0001, respectively [43]. Also, Qi et al. (2000) indicated no clear association between dietary TP intake and risk of all-cause, CVD, and cancer mortality. Additionally, higher PP intake was associated with a reduced risk of CVD mortality (RR: 0.88, 95% CI: 0.80–0.96, I^2^ = 63.7%, *P* = 0.001) [44]. Li et al. (2017) revealed that PP in substitution for AP decreased LDL level by 0.16 mmol/L (95% CI, − 0.20 to − 0.12 mmol/L; *P* < 0.00001) and non–HDL by 0.18 mmol/L (95% CI, − 0.22 to − 0.14 mmol/L; *P* < 0.00001) [45]. Wirunsawanya et al. (2018) concluded that whey protein can lower HDL, TC, and BG [46]. Similarly, Berthold et al. (2011) found that TG decreased after whey protein consumption [47]. Aune et al. (2016) revealed that PP helps decrease MetS and CVD if consumed regularly [48]. Zhao et al. (2016) demonstrated that fish consumption as a source of AP contributes to CVD risk reduction [49]. Asbaghi et al. (2022) also showed that soy protein can improve cardiovascular parameters in patients with T2DM [11]. Zhou et al. (2024) reported that high-quality proteins, including Milk protein supplementation, decreased SBP (− 2.30 [−3.45, − 1.15] mmHg) and TC (− 0.27 [−0.51, − 0.03] mmol/L). Whey supplementation decreased SBP (−2.20 [−3.89, −0.51] mmHg), DBP (−1.07 [−1.98, −0.16] mmHg), TG (−0.10 [−0.17, −0.03] mmol/L), TC (−0.18 [−0.35, −0.01] mmol/L), LDL (−0.09 [−0.16, −0.01] mmol/L) and fasting blood insulin (FBI) (−2.02 [−3.75, −0.29] pmol/L). Casein supplementation decreased SBP (−4.10 [−8.05, −0.14] mmHg) [50]. Ye et al. (2019) claimed that higher intake of PP did not affect T2DM risk (RR: 0.93; *P* = 0.074), whereas moderate intake can reduce risk of disease (RR: 0.94; *P* < 0.001) [51]. In contrast with our study results, Ye et al. (2019) indicated that high TP and AP intake can increase the risk of T2DM (RR: 1.10; *P* = 0.006) and (RR: 1.13; *P* = 0.013), respectively. Whereas moderate TP and AP intake did not affect T2DM (RR: 1.00; *P* = 0.917),(RR: 1.06; *P* = 0.058), respectively [51]. Zhang et al. (2023) revealed that processed meat was associated with a higher risk of all-cause mortality, CVD, and MetS components [52].

Protein typically increases satiety, delays gastrointestinal emptying, and improves postprandial thermogenesis, which may contribute to decreased food intake, improved insulin sensitivity, and lipid profiles [17, 18]. For example, PP can mostly improve MetS because it contains soluble fiber, saponins [53], polyphenols [54], steroids [55], phytoestrogens [56], phytates [57], bioactive compounds (e.g., isoflavones and beta-glucanin) [58], glutamic acid, and antioxidants [59], which have lipid-lowering properties that can reduce obesity-related parameters and improve lipid profile, reduce arterial stiffness, improve BP, and insulin sensitivity. These functions are mechanistically regulated by peroxisome proliferator-activated receptor (PPAR)-regulated gene expression, specifically the sterol regulatory element binding protein (SREBP) [60, 61]. Notably, the net effect of AP may depend on the specific food source, the overall dietary matrix, and population-specific factors. For instance, seafoods consist of Omega-3, polyunsaturated fatty acids (PUFAs), eicosapentaenoic acid (EPA), docosahexaenoic acid (DHA), and vitamin D, which can lower TG and inflammation effects [40, 41, 62]. PUFAs can prevent nuclear transcription factor kappa B (NF-kB), which is a key factor in cytokine gene expression and inflammation [63]. Dairy products contain whey (WP) and casein proteins, which have AAs tryptophan and tyrosine [64], and also linolenic acid (ALA), reducing the risk of MetS and its components [65]. WP also inhibits acyl-CoA:1,2-diacylglycerol acyltransferase (DGAT-2), increases lipolytic activity of lipoprotein lipase, and induces fatty oxidation genes in extrahepatic tissues that finally lead to TG lowering [66, 67]. However, red meat (RM) contains high levels of methionine and branched-chain amino acids (BCAAs) [68], saturated fatty acids (SFA), and heme iron, which increase the risk of T2DM, MetS, and increased BW [69–71]. Iron can potentially reinforce oxidative stress and subsequently lead to insulin resistance [72]. Furthermore, some additives, including nitrates and nitrites, can be converted into nitrosamines, and sodium, which are found in processed red meat (PRM), also contribute to impaired insulin response and BP [73, 74]. There are also some factors that can play a key role regarding the high heterogeneity in the current study; firstly, differences among countries regarding food cultures and dietary choices, age, medical history, levels of activity, and duration of consumption. For instance, men below 50 years are more prone to get MetS, and women after 50 years, because after menopause, they are more affected by hormone fluctuations through changes in biological pathways and response to socio-economic status [75]. Additionally, baseline health of participants can be affected by another factor, for instance, it is reported that Chinese people do more vigorous activity, walking, and sleeping, but fewer hours sitting than in North America [76]. Typical dietary choices based on regions and food culture can impact the prevalence of MetS; for instance, Asian people consume more fruits, aquatic foods, and the southern dietary pattern is characterized by high PUFAs, dietary fibers, vitamins, and minerals, and less saturated fat and sodium, which can reduce the risk of MetS among these populations [77]. Despite Asian diets, Western diets contain excessive fat, sugary foods, refined grains, fast foods, and ultra-processed foods (UPFs) such as PRM, which make them more susceptible to developing MetS [36]. Duration of consumption can also affect the MetS components; for instance, a 13-year follow-up showed a direct and considerable association with lipid profile than short-term durations [12]. Different tools’ assessment can also cause heterogeneity, for instance, FFQ shows long-term dietary patterns, despite the 24-h dietary recall, which shows short-term data. They are also more validated across Western populations and have limited accuracy when applied to Asian society [78]. Besides, there may be reporting bias because people mostly underreport unhealthy foods and overreport healthy foods [79]. In addition, the quality and quantity of different sources of macronutrients can affect MetS components and our overall well-being. For instance, Zhou et al. (2024) [50] showed that the quality of protein can be effective, and Ye et al. (2019) [51] depicted that the quantity of protein can modify its impact.

### Strengths

This current study is a systematic review and meta-analysis that explored the association between AP, PP, TP, and MetS, which is not limited to a specific gender, age group, medical record, or type of protein. We also performed subgroup analysis according to some factors that present the source of heterogeneity. Furthermore, the NOS assessment showed that all included studies were of high methodological quality [20], which allowed us to control for confounding variables and detect different possible interactions more accurately. Finally, we also applied the GRADE framework to evaluate evidence quality and consistency. Following this, our findings achieve increased scientific credibility and clinical relevance, supporting their reliability in clinical and public health contexts.

### Limitations

However, should it be considered, as this study was broad in scope, we could not concentrate on the exact food groups in detail. Notably, subgroup and meta-regression analyses provided insight into some sources of heterogeneity. Additionally, the definitions of “high” protein intake varied across studies (e.g., quartiles, tertiles, etc.). This variability was also considered as another source of heterogeneity in the pooled analyses. Given the high heterogeneity and significant bias in PP-MetS and PP-TG, the findings may not be generalizable to all populations and should be interpreted with caution in clinical settings. Besides, this heterogeneity may be affected by the potential effect of unknown confounders, residual effects among studies, differences in study design, populations, intervention doses, and outcome definitions. Additionally, these analyses may have been underpowered due to the limited number of included studies in certain categories. Following these, it is suggested that differences among protein sources and individual characteristics may be important considerations for future dietary guidelines, rather than focusing on protein intake. Besides, personalized dietary strategies could help optimize protein choices according to their medical history, genetics, metabolism, and gut microbiota profile. Advances in dietary assessment tools and biomarker validation may help reduce heterogeneity across studies and provide stronger evidence for causal inference.

## Conclusion

Current evidence suggests that PP and AP intake may be associated with reduced risk of MetS as the primary outcome. However, in specific contexts, such as some of the secondary outcomes, results showed no reaction, e.g., TP-WC, TP-FBS, TP-BP, AP-FBS, PP-FBS. Besides, due to the high heterogeneity, methodological quality, and significant bias in PP-MetS and PP-TG, recommendations must be made cautiously. Finally, no definitive conclusions can be drawn regarding a causal or uniform protective relationship.

## Supplementary Information

Supplementary Material 1

Supplementary Material 2

## Data Availability

No datasets were generated or analysed during the current study.

## Abbreviations

MetS: Metabolic syndrome
PP: Plant protein
TP: Total protein
AP: Animal protein
HDL: High-density lipoprotein
LDL: Low-density lipoprotein
BP: Blood pressure
FBG: Fasting blood glucose
WC: Waist circumference
CVD: Cardiovascular disease
DM: Diabetes mellitus

## Declarations

### Applying artificial intelligence chatbots

We used an AI-powered tool named Grammarly to evaluate the grammatical and academic quality of the text that the authors originally wrote.
