# Supplementary material for: The association between dietary protein intake and metabolic syndrome: a GRADE-assessed systematic review and meta-analysis of observational studies
Source: Diabetol Metab Syndr. 2025 Dec 29;18:41. doi: 10.1186/s13098-025-02011-0 (PMC12866194; doi:10.1186/s13098-025-02011-0)
Supplement: Supplementary file 1 — Supplementary Material 1 [file 13098_2025_2011_MOESM1_ESM.docx]

**Contents**

**Supplementary Figures for Primary Outcomes** 3

**Supplementary Fig. 1**. Funnel plot for the association of TP and Mets**3**

**Supplementary Fig. 2**. The sensitivity analysis for TP and Mets 3

**Supplementary Fig. 3.** The funnel plot for AP and Mets4

**Supplementary Fig. 4.** The sensitivity analysis for AP and Mets4

**Supplementary Fig. 5.** The funnel plot for PP and Mets5

**Supplementary Fig. 6.** The sensitivity analysis for PP and Mets5

**Supplementary Figures for Secondary Outcomes** 6

**Supplementary Fig. 7**. The random model for TP and TG6

**Supplementary Fig. 8.** The funnel plot for TP and TG6

**Supplementary Fig. 9.** The sensitivity analysis for TP and TG7

**Supplementary Fig. 10**. The random model for the association of TP and HDL7

**Supplementary Fig. 11.** The funnel plot for TP and HDL8

**Supplementary Fig. 12.** The sensitivity analysis for TP and HDL8

**Supplementary Fig. 13.** The random model for the association of TP and WC9

**Supplementary Fig. 14.** Funnel plot for TP and WC9

**Supplementary Fig. 15.** The sensitivity analysis for TP and WC10

**Supplementary Fig. 16.** The random model for the association of TP and FBS10

**Supplementary Fig. 17**. The funnel plot for the association of TP and FBS11

**Supplementary Fig. 18**. The sensitivity analysis for the association of TP and FBS11

**Supplementary Fig. 19**. The random model for the association of TP and BP12

**Supplementary Fig. 20**. The funnel plot for the association of TP and BP12

**Supplementary Fig. 21.** The sensitivity analysis for the association of TP and BP13

**Supplementary Fig. 22.** The random model for AP and TG13

**Supplementary Fig. 23.** The funnel plot for AP and TG14

**Supplementary Fig. 24**. The sensitivity analysis for AP and TG14

**Supplementary Fig. 25.** The random model for AP and HDL15

**Supplementary Fig. 26.** The funnel plot for AP and HDL15

**Supplementary Fig. 27.** The sensitivity analysis for AP and HDL16

**Supplementary Fig. 28.** The random model for AP and WC16

**Supplementary Fig. 29.** The funnel plot for AP and WC17

**Supplementary Fig. 30.** The sensitivity analysis for AP and WC17

**Supplementary Fig. 31.** The random model for AP and FBS18

**Supplementary Fig. 32**. The funnel plot for AP and FBS18

**Supplementary Fig. 33.** The sensitivity analysis for AP and FBS19

**Supplementary Fig. 34.** A. The random model for AP and BP19

**Supplementary Fig. 35.** The funnel plot for AP and BP20

**Supplementary Fig. 36.** The sensitivity analysis for AP and BP20

**Supplementary Fig. 37.** The random model for PP and TG21

**Supplementary Fig. 38.** The funnel plot for PP and TG21

**Supplementary Fig. 39.** The sensitivity analysis for PP and TG22

**Supplementary Fig. 40**. The random model for PP and HDL22

**Supplementary Fig. 41.** The funnel plot for PP and HDL23

**Supplementary Fig. 42.** The sensitivity analysis for PP and HDL23

**Supplementary Fig. 43**. The random model for PP and WC24

S**upplementary Fig. 44.** The funnel plot for PP and WC24

**Supplementary Fig. 45.** The sensitivity analysis for PP and WC25

**Supplementary Fig. 46**. The random model for PP and FBS25

S**upplementary Fig. 47.** The funnel plot for PP and FBS26

**Supplementary Fig. 48.** The sensitivity analysis for PP and FBS26

**Supplementary Fig. 49**. The random model for PP and BP27

**Supplementary Fig. 50.** The funnel plot for PP and BP27

**Supplementary Fig. 51.** The sensitivity analysis for PP and BP28


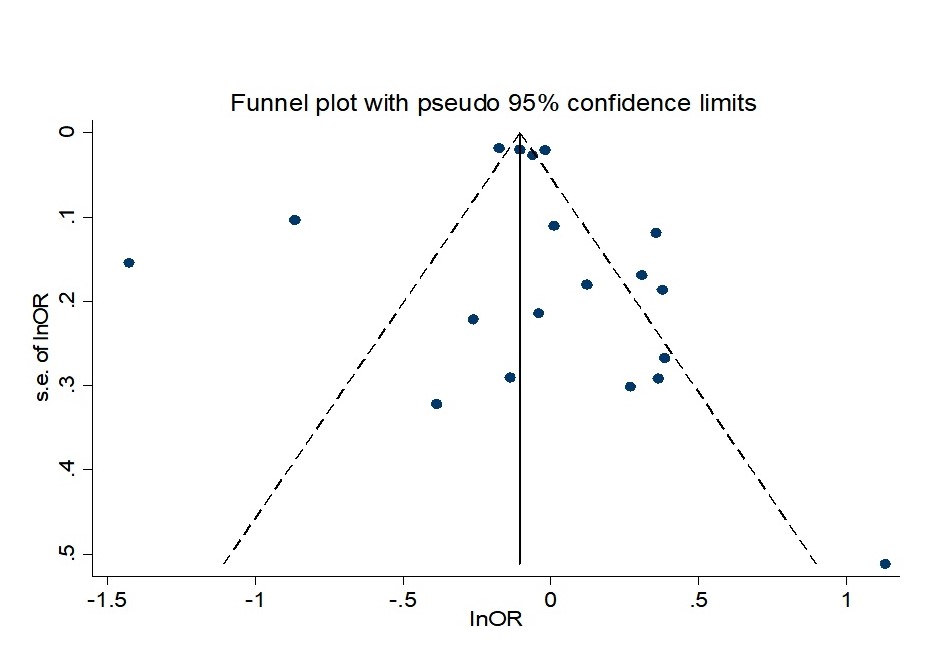


**Supplementary Fig. 1**. Funnel plot for the association of TP and Mets


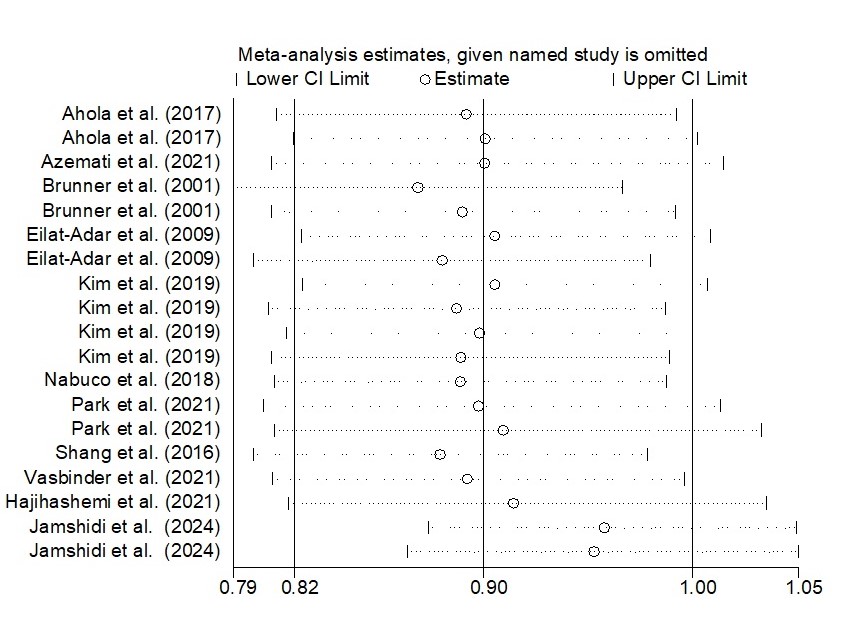


**Supplementary Fig. 2**. The sensitivity analysis for TP and Mets


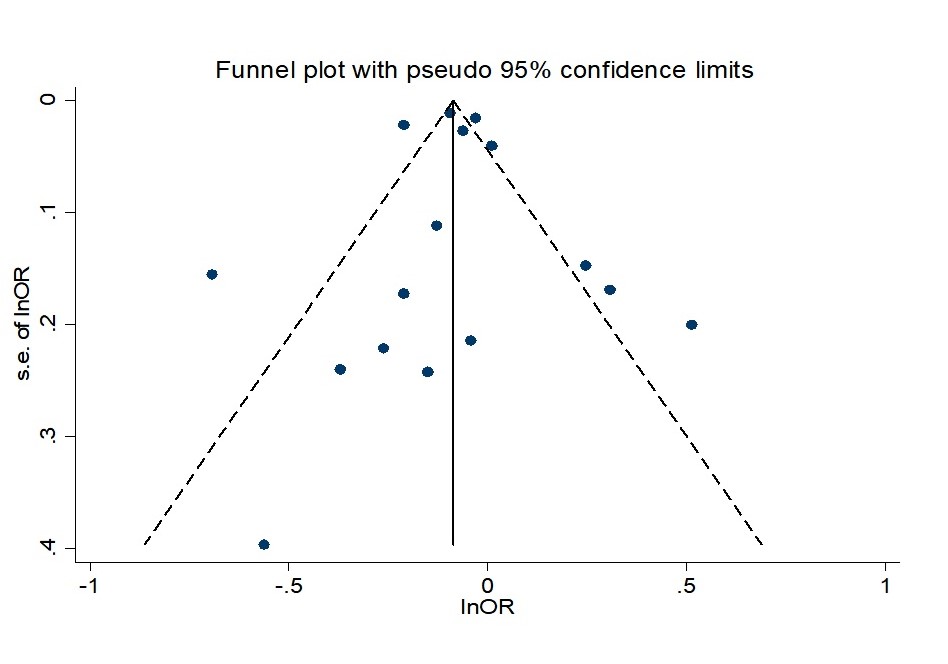


**Supplementary Fig. 3.** The funnel plot for AP and Mets


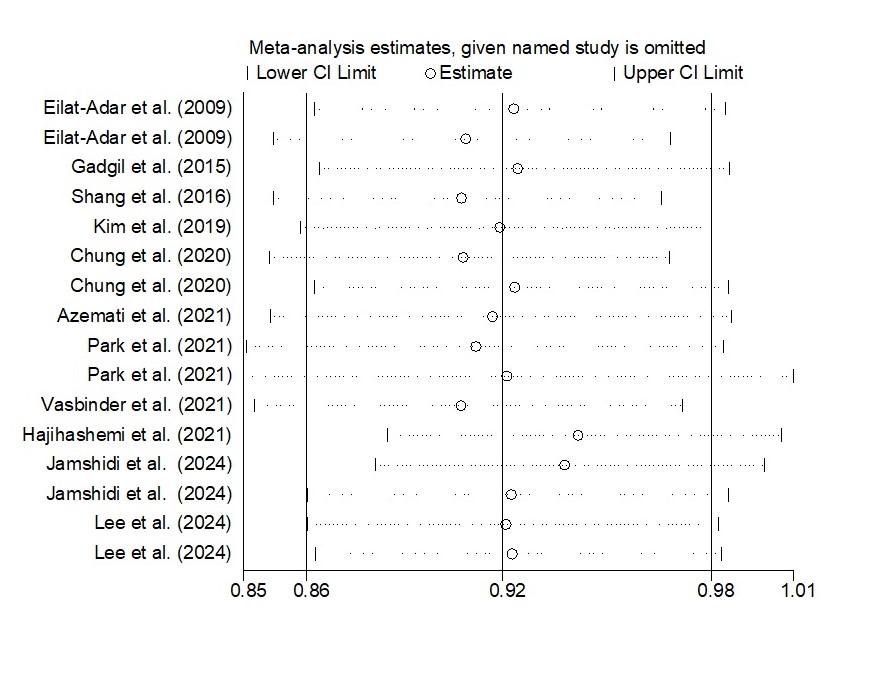


**Supplementary Fig. 4.** The sensitivity analysis for AP and Mets


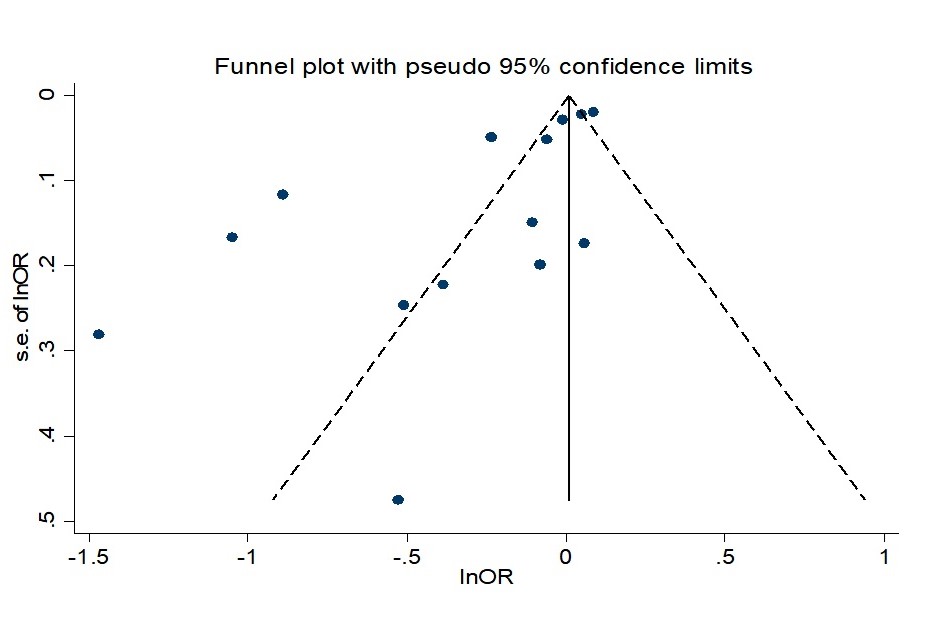


**Supplementary Fig. 5.** The funnel plot for PP and Mets


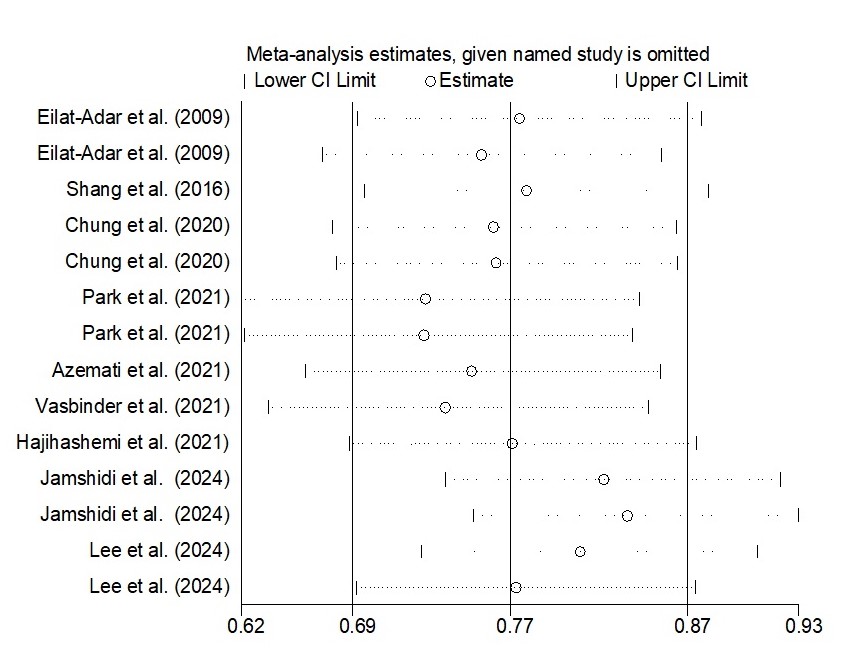


**Supplementary Fig. 6.** The sensitivity analysis for PP and Mets


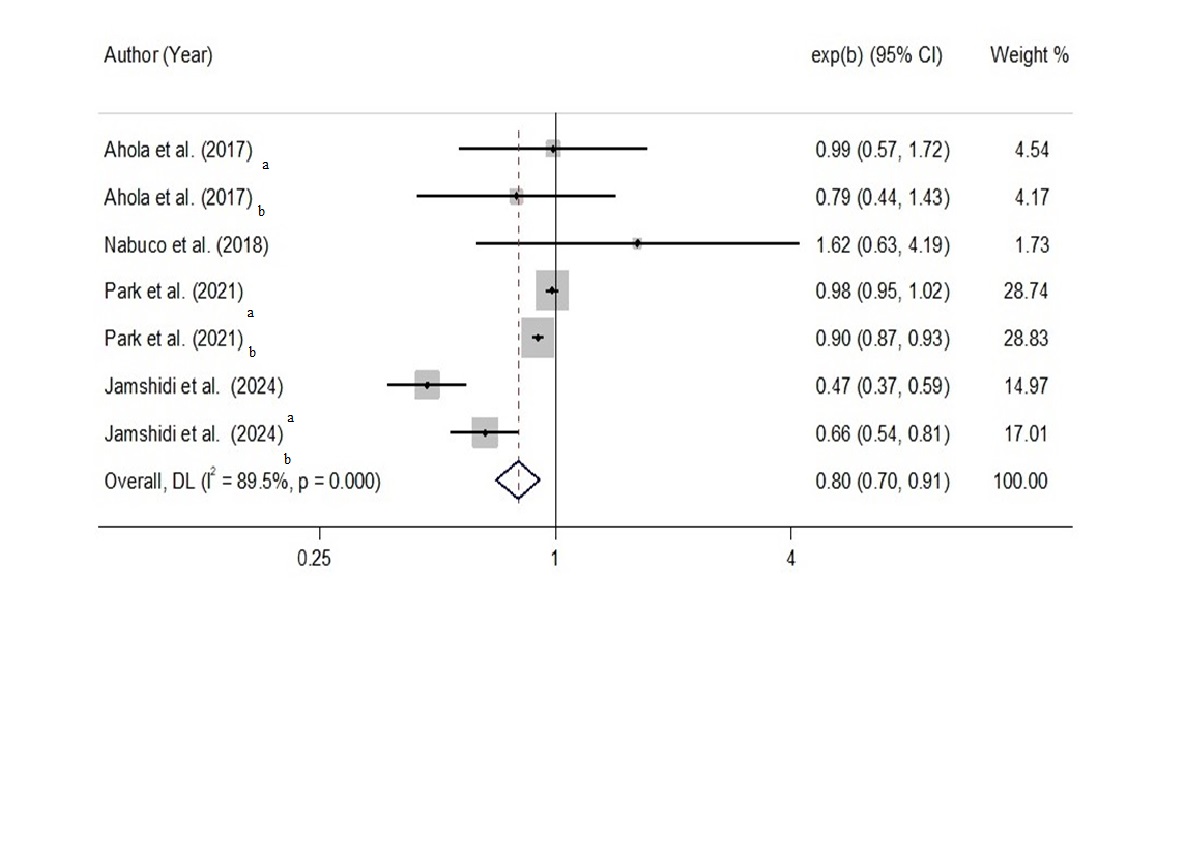
**Supplementary Fig. 7**. The random model for TP and TG, a: males, b: females


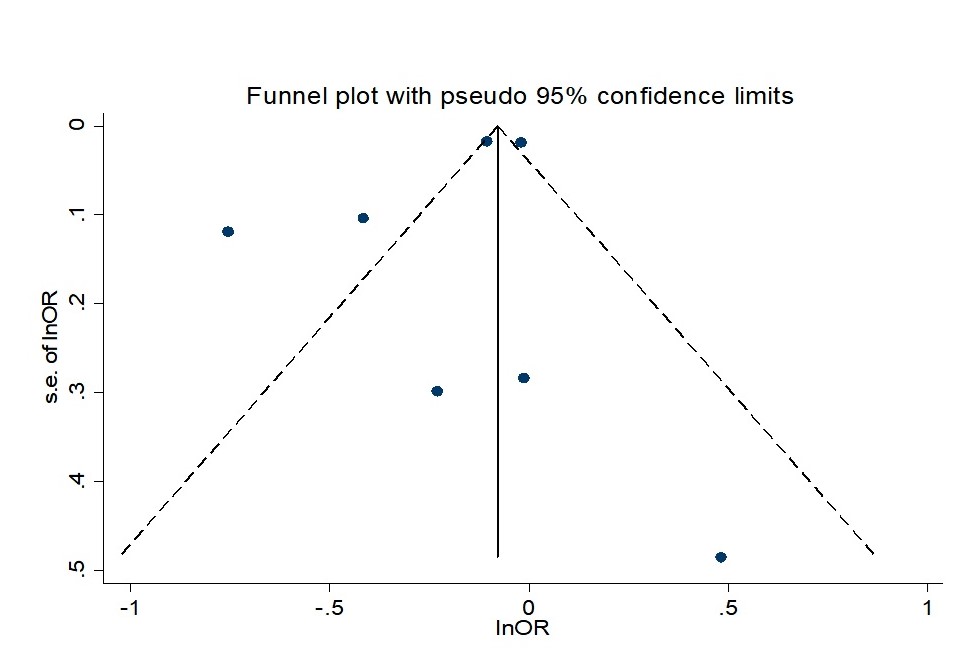


**Supplementary Fig. 8.** The funnel plot for TP and TG


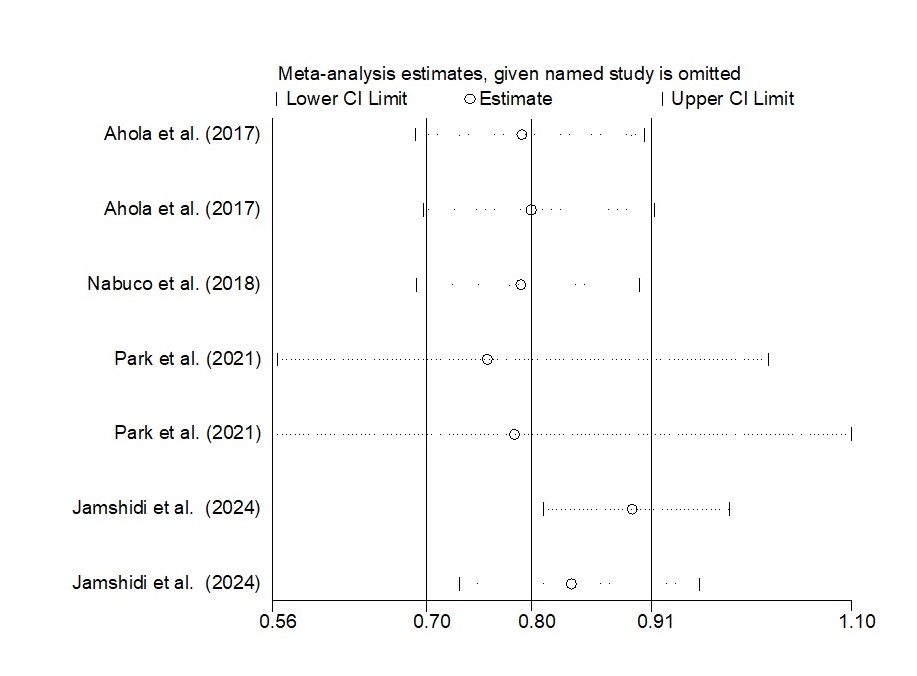


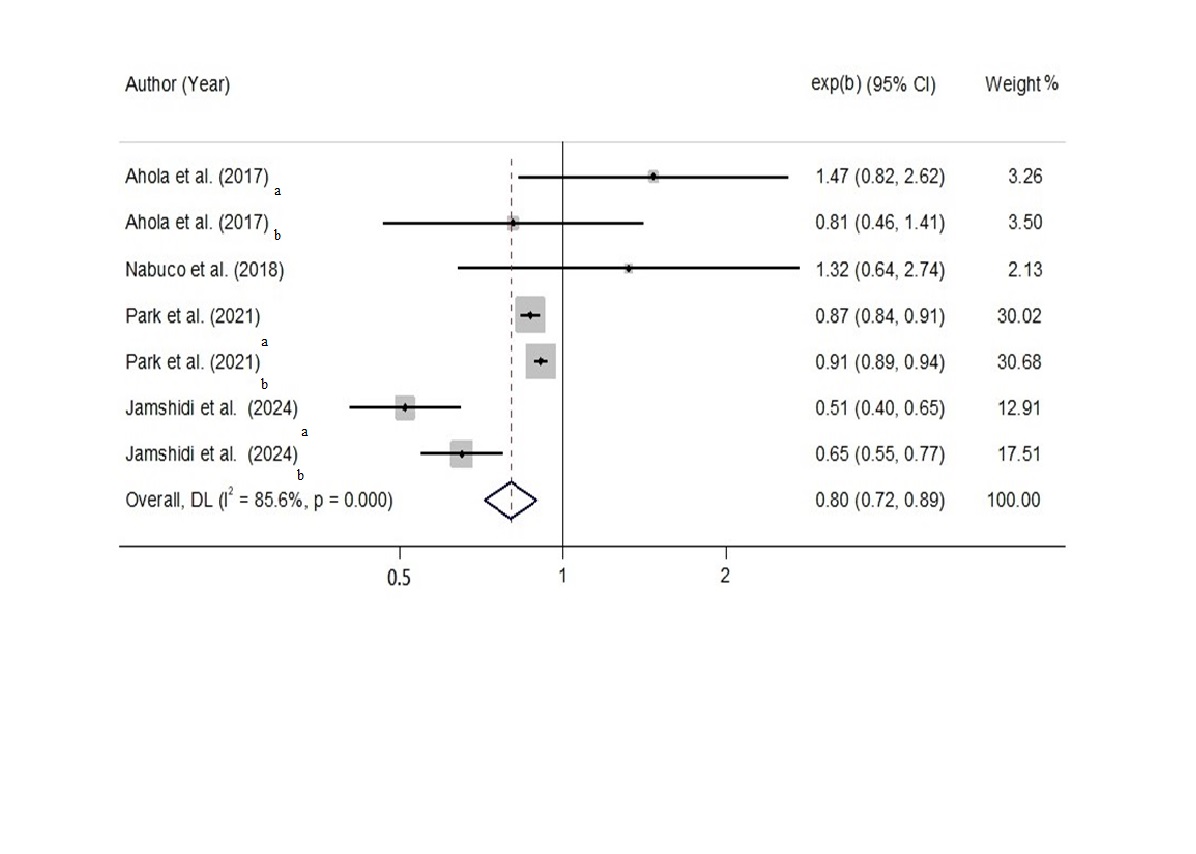
 **Supplementary Fig. 9.** The sensitivity analysis for TP and TG

**Supplementary Fig. 10**. The random model for the association of TP and HDL, a: males, b: females


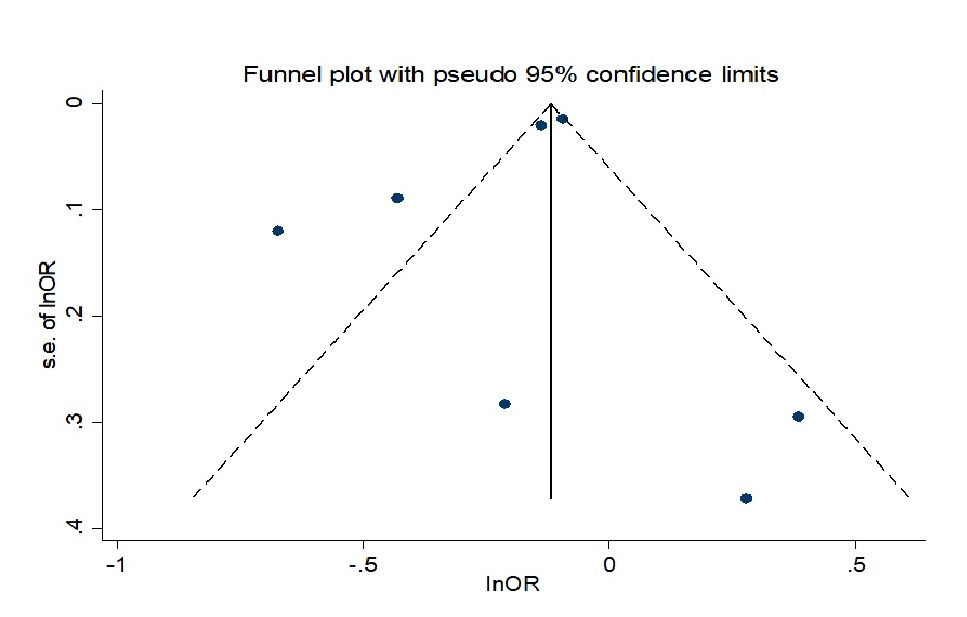


**Supplementary Fig. 11.** The funnel plot for TP and HDL


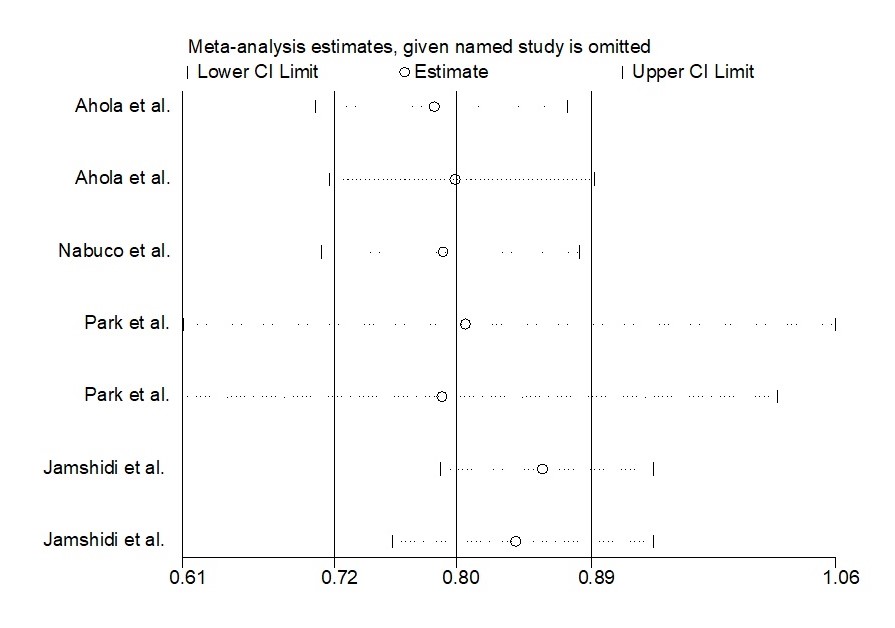


**Supplementary Fig. 12.** The sensitivity analysis for TP and HDL


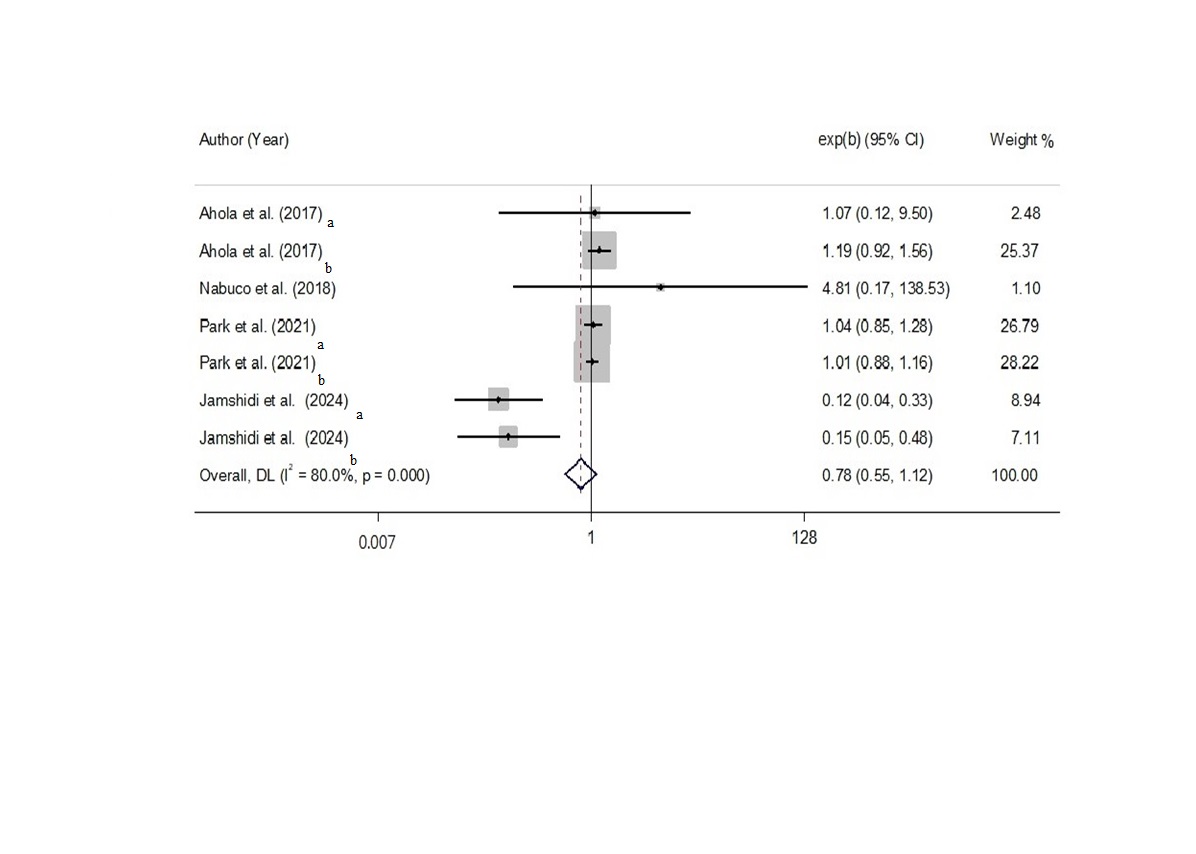


**Supplementary Fig. 13.** The random model for the association of TP and WC, a: males, b: females


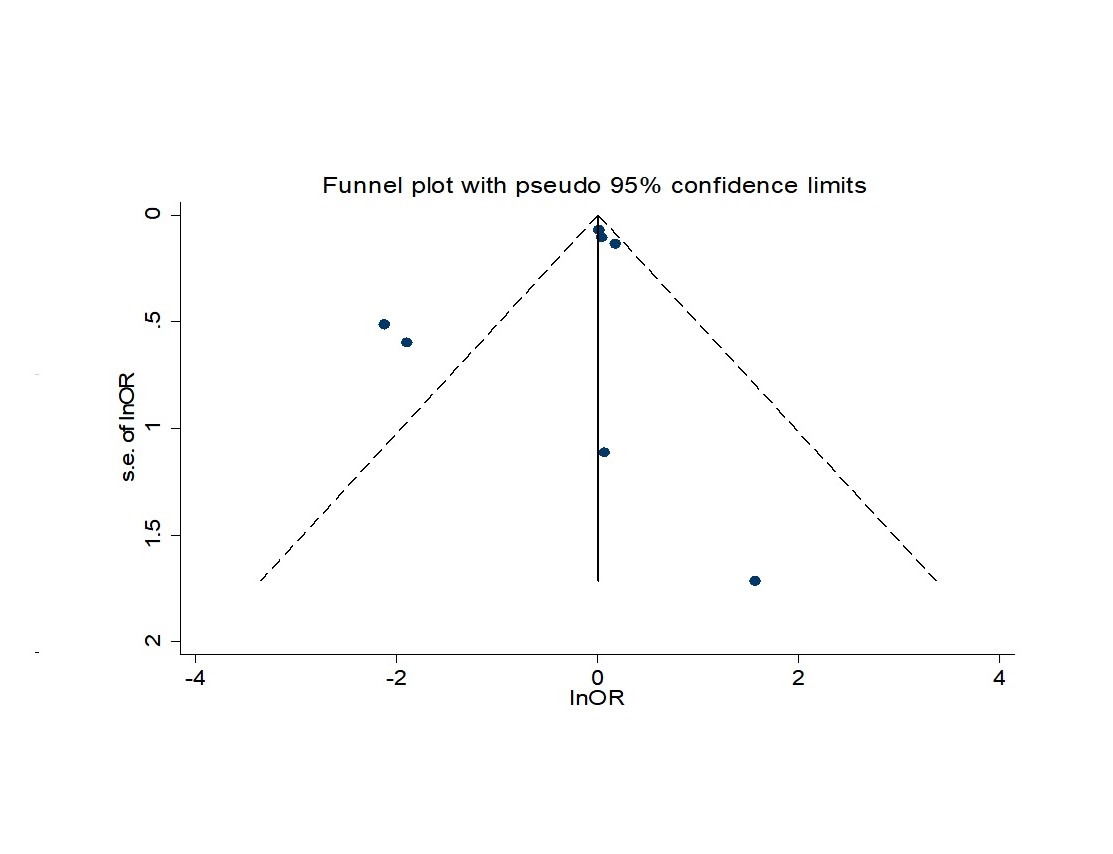


**Supplementary Fig. 14.** Funnel plot for TP and WC


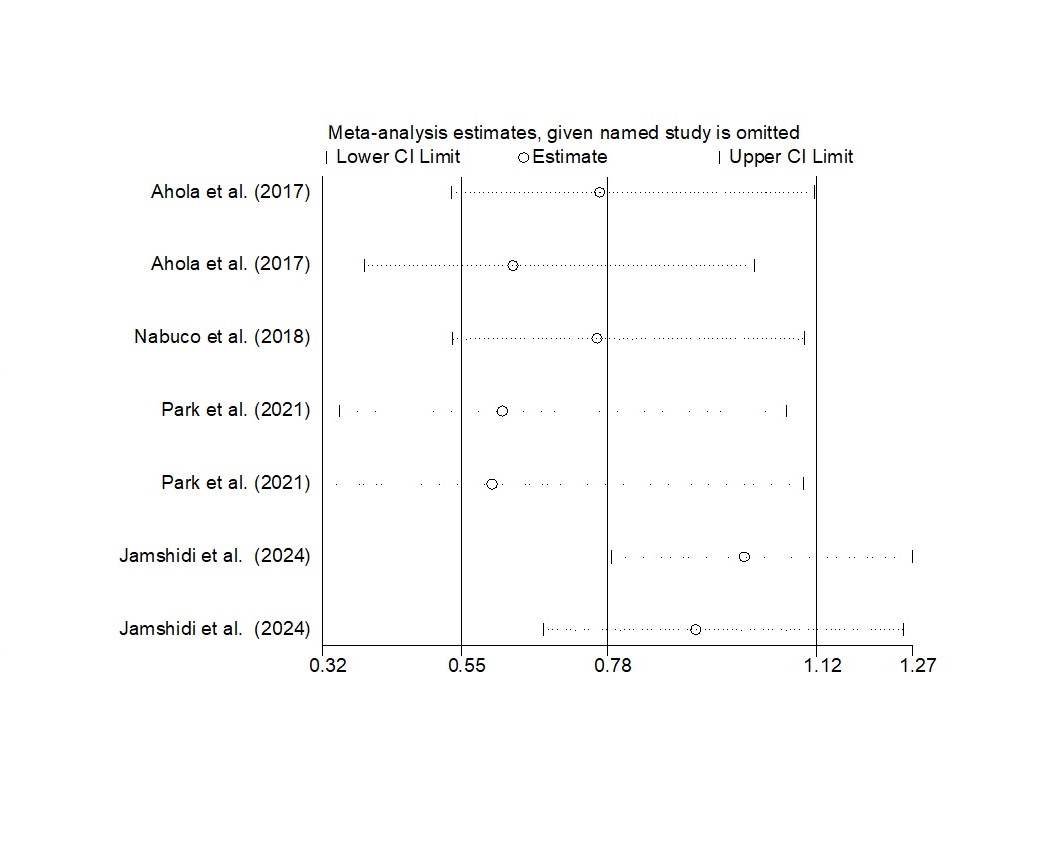


**Supplementary Fig. 15.** The sensitivity analysis for TP and WC


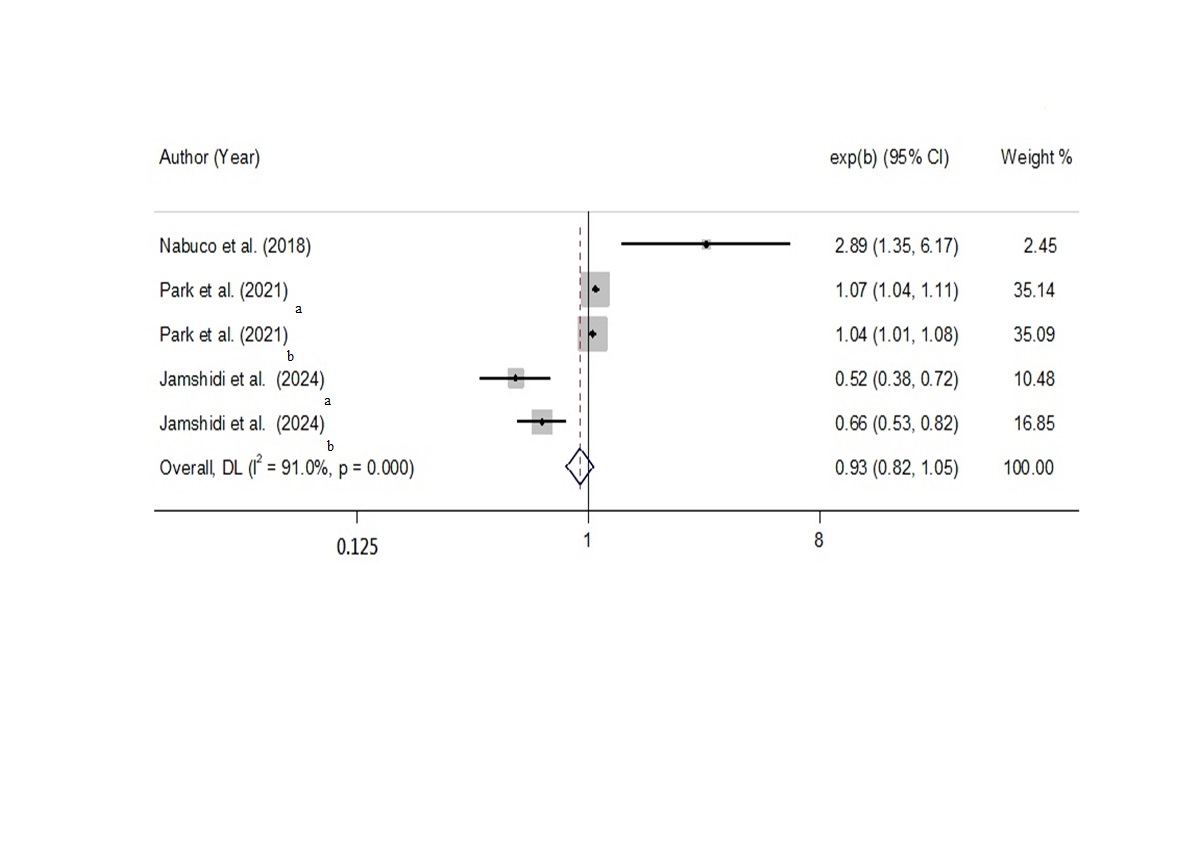


**Supplementary Fig. 16.** The random model for the association of TP and FBS, a: males, b: females


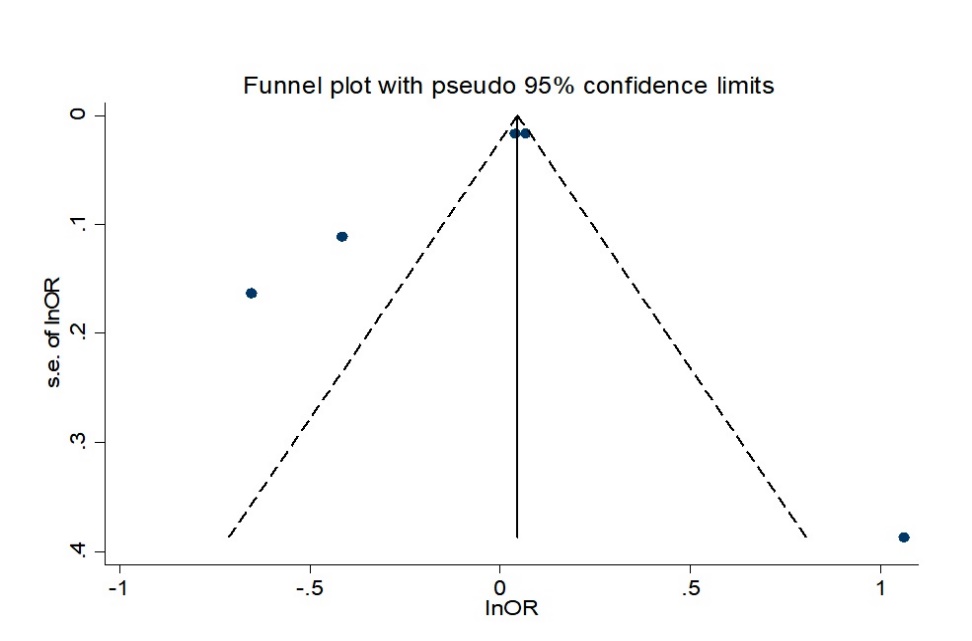


**Supplementary Fig. 17**. The funnel plot for the association of TP and FBS


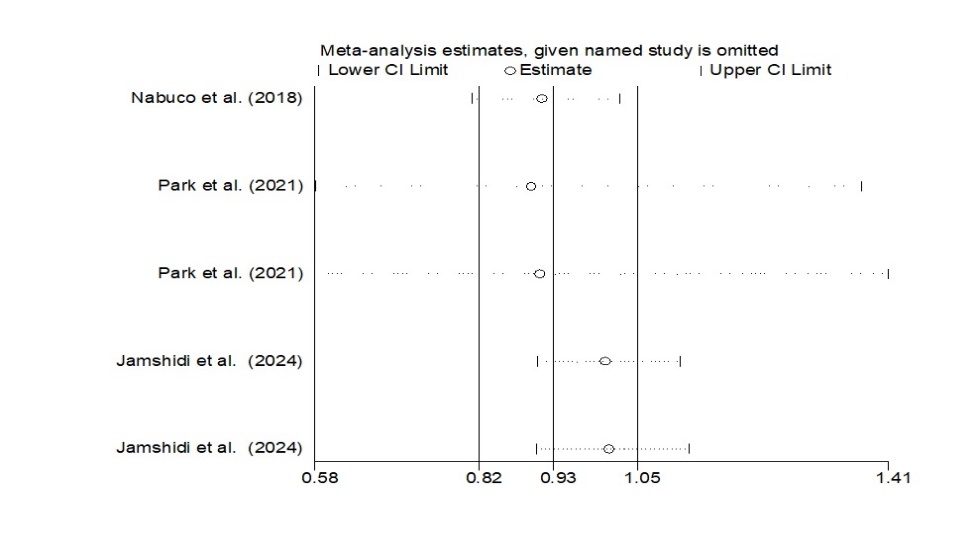


**Supplementary Fig. 18**. The sensitivity analysis for the association of TP and FBS


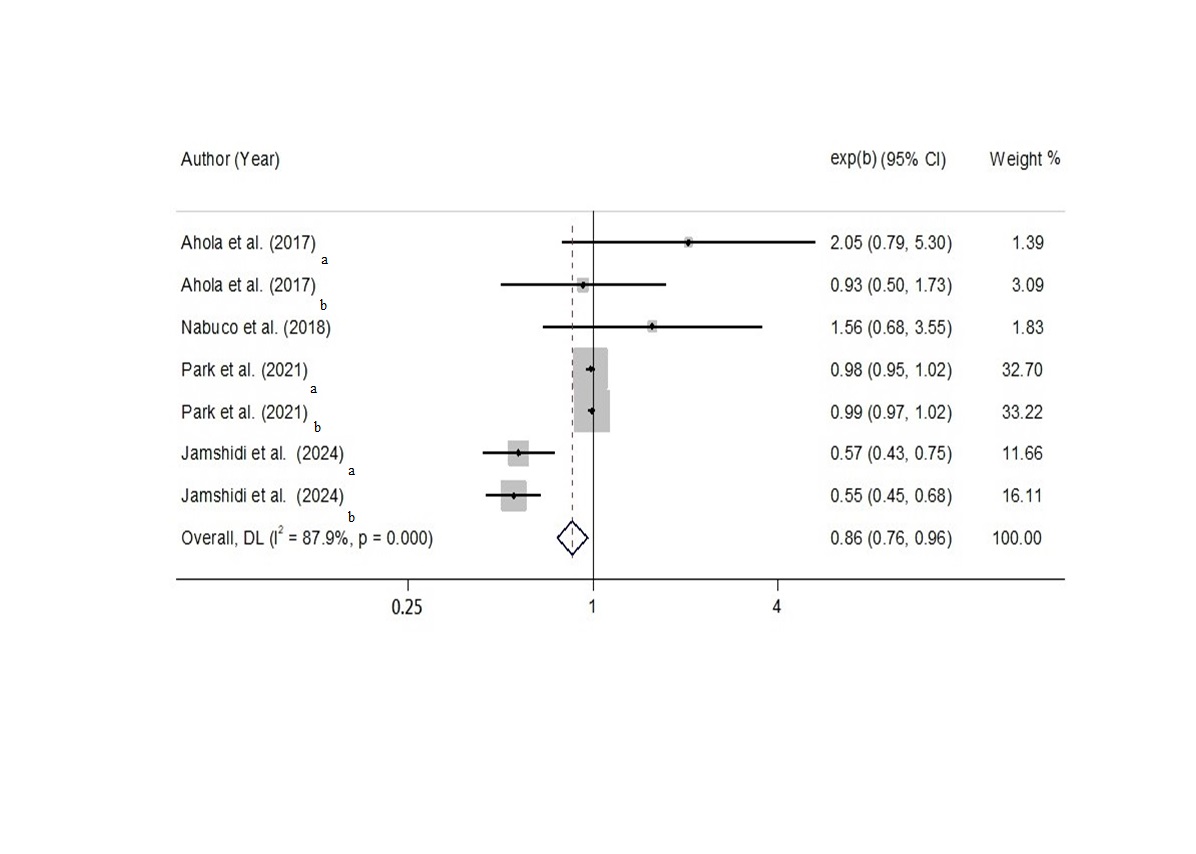


**Supplementary Fig. 19**. The random model for the association of TP and BP, a: males, b: females


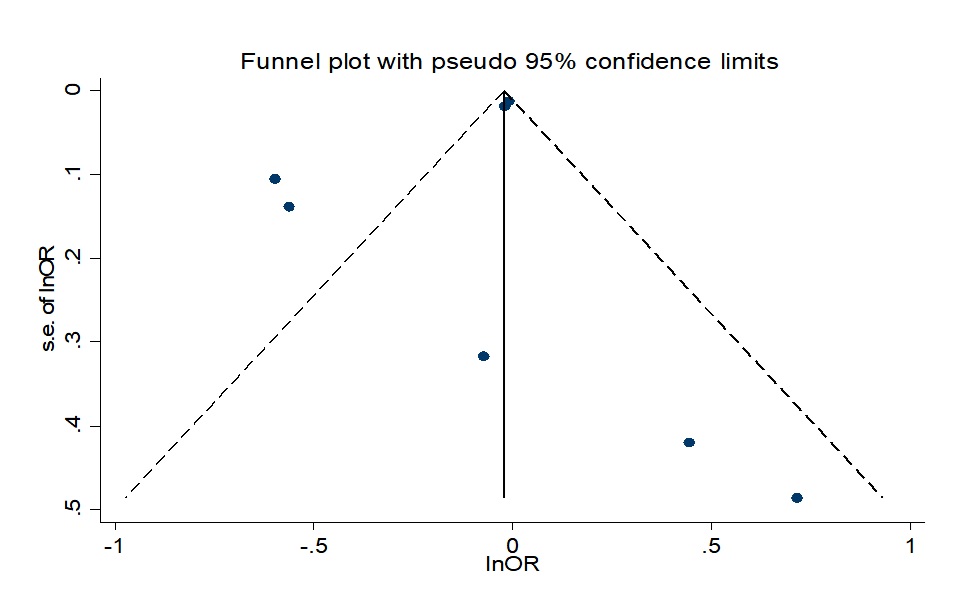


**Supplementary Fig. 20**. The funnel plot for the association of TP and BP


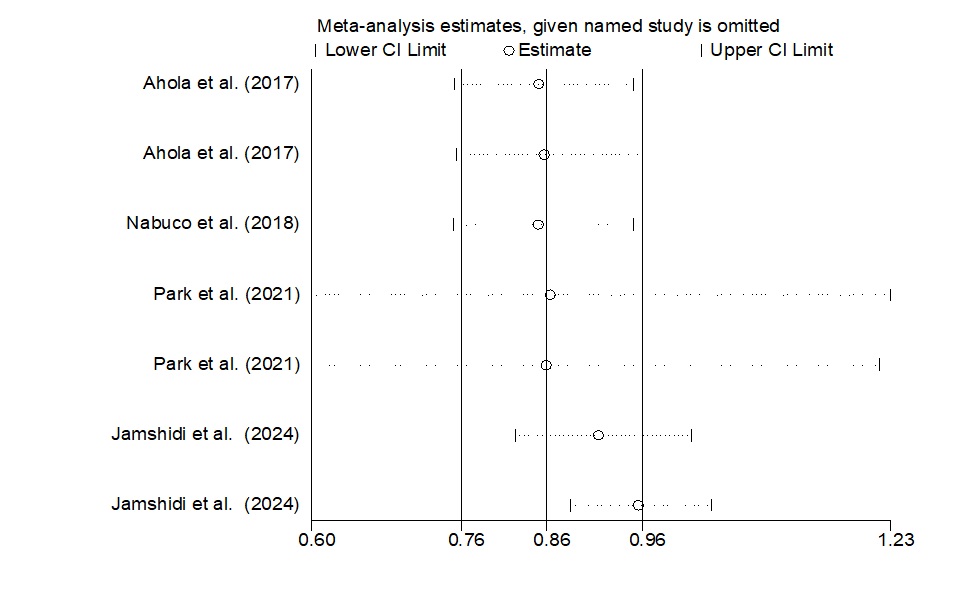


**Supplementary Fig. 21.** The sensitivity analysis for the association of TP and BP

**Supplementary Fig. 22.** The random model for AP and TG, a: males, b: females


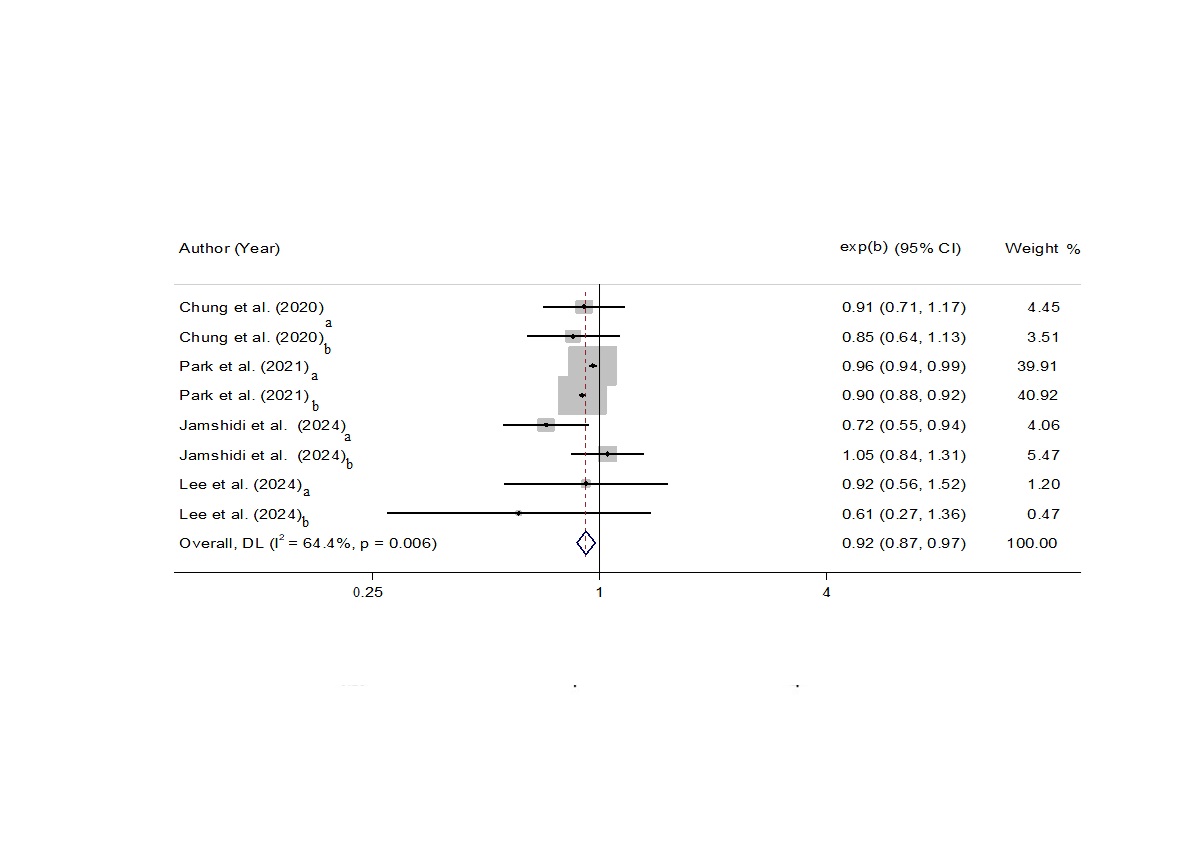


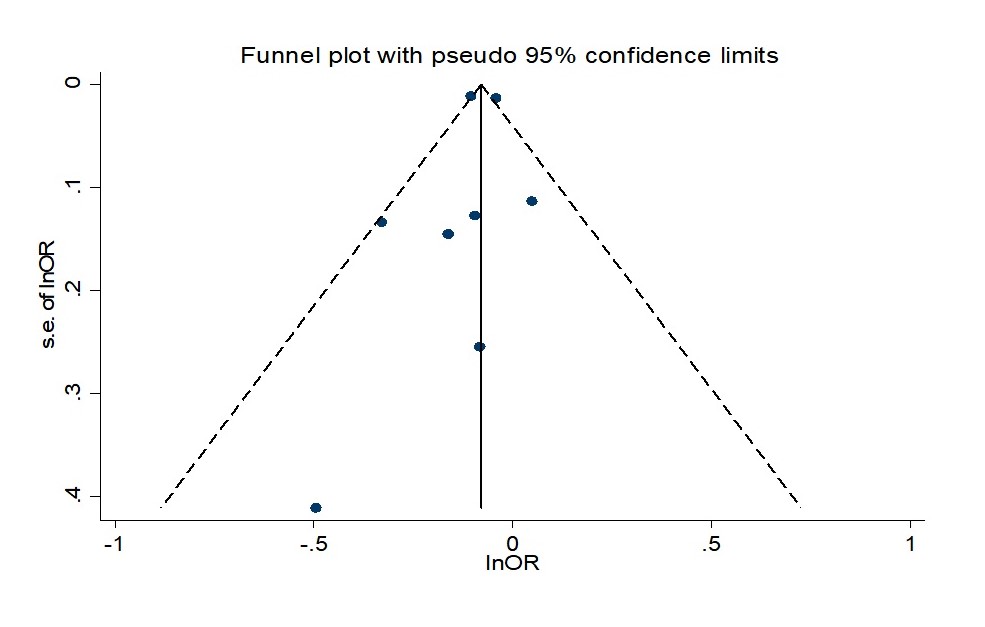


**Supplementary Fig. 23.** The funnel plot for AP and TG


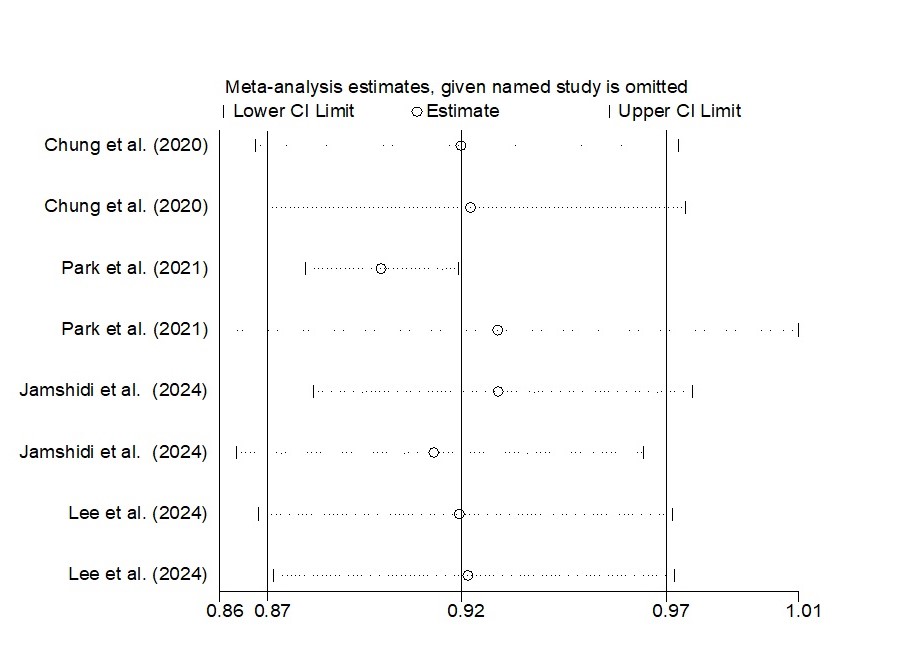


**Supplementary Fig. 24**. The sensitivity analysis for AP and TG


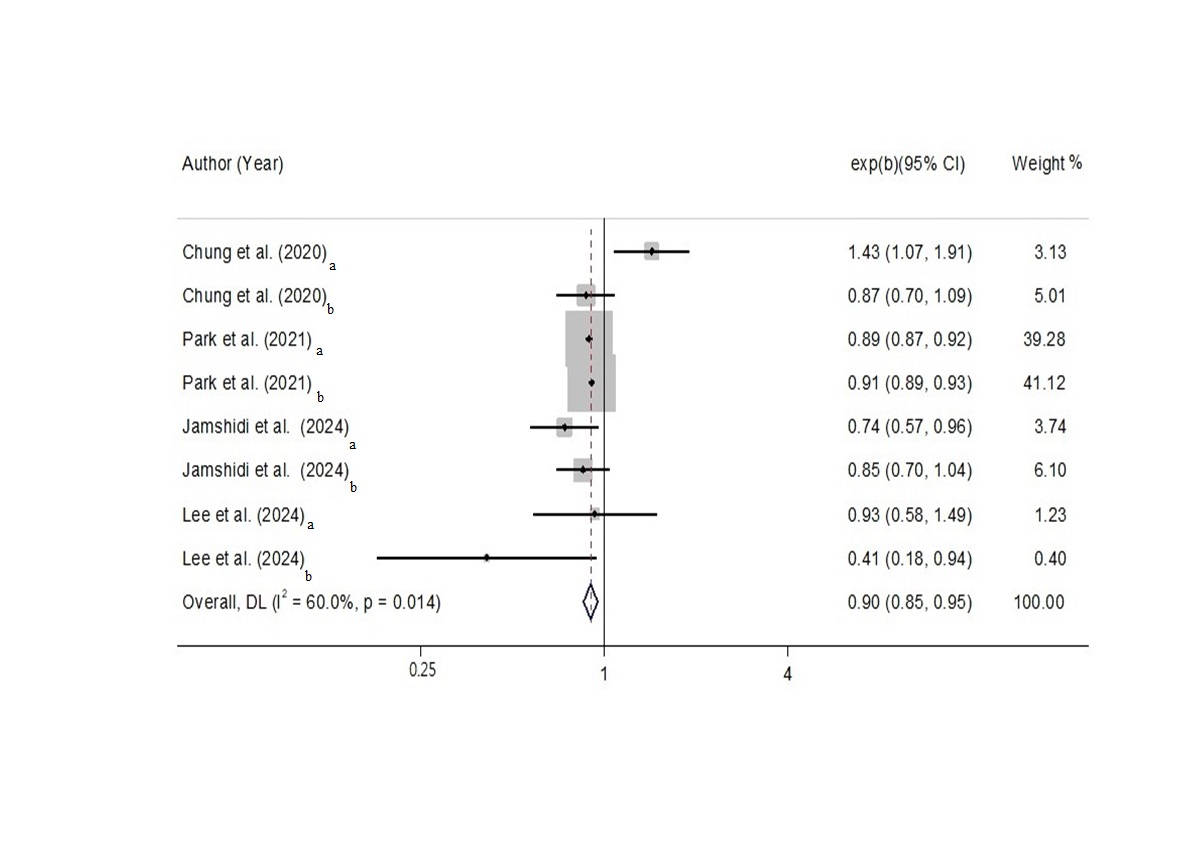


**Supplementary Fig. 25.** The random model for AP and HDL, a: males, b: females


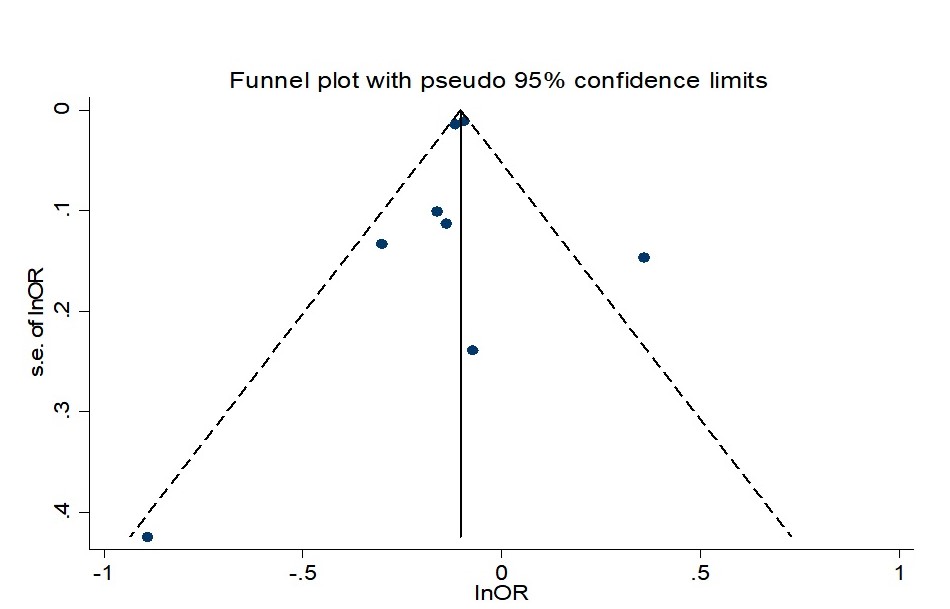


**Supplementary Fig. 26.** The funnel plot for AP and HDL


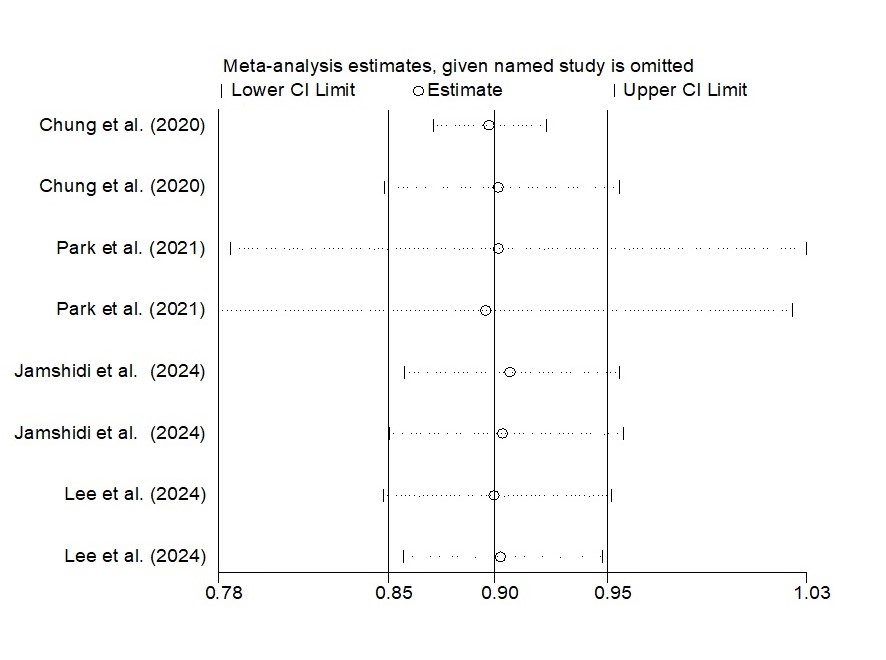


**Supplementary Fig. 27.** The sensitivity analysis for AP and HDL


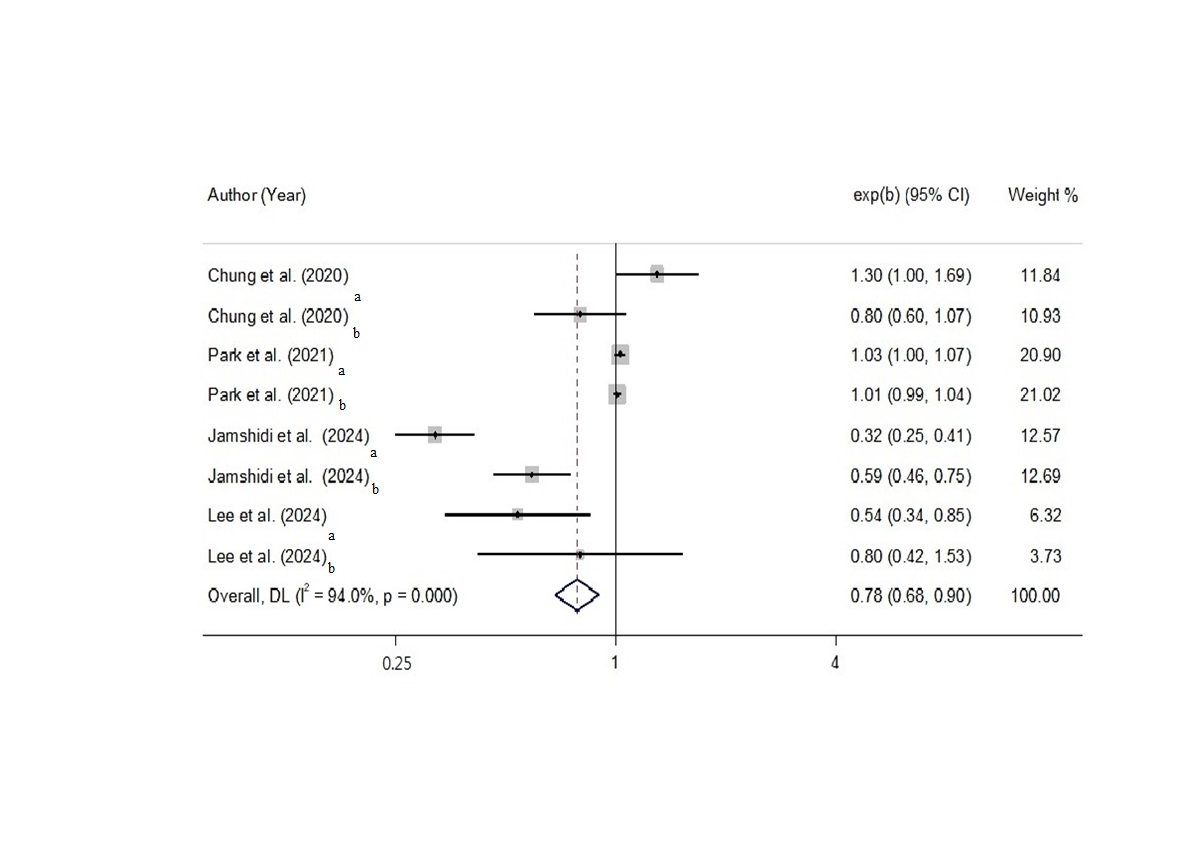


**Supplementary Fig. 28.** The random model for AP and WC, a: males, b: females


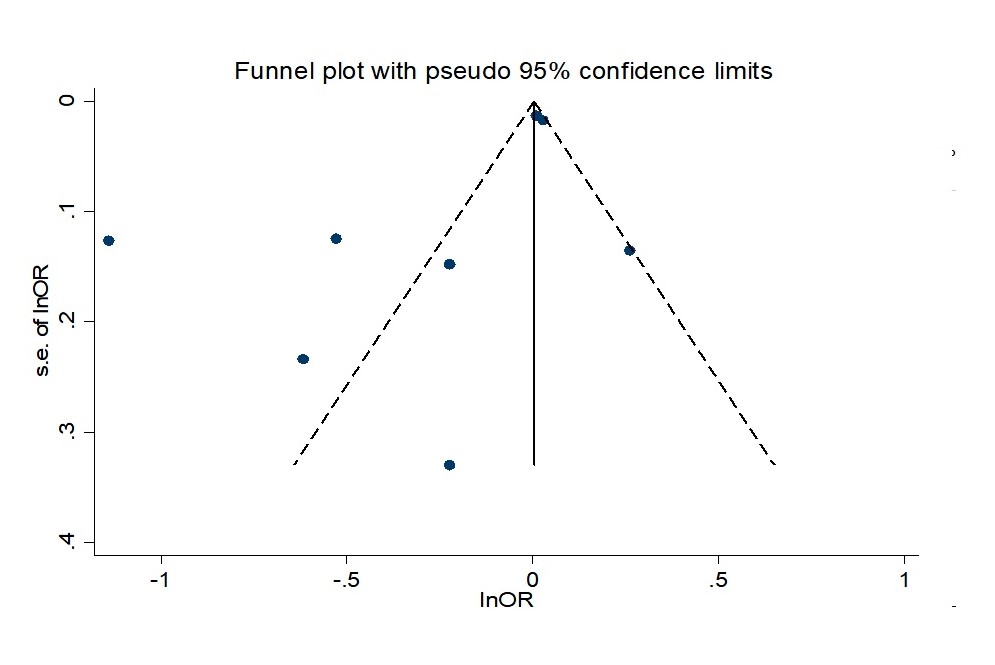


**Supplementary Fig. 29.** The funnel plot for AP and WC


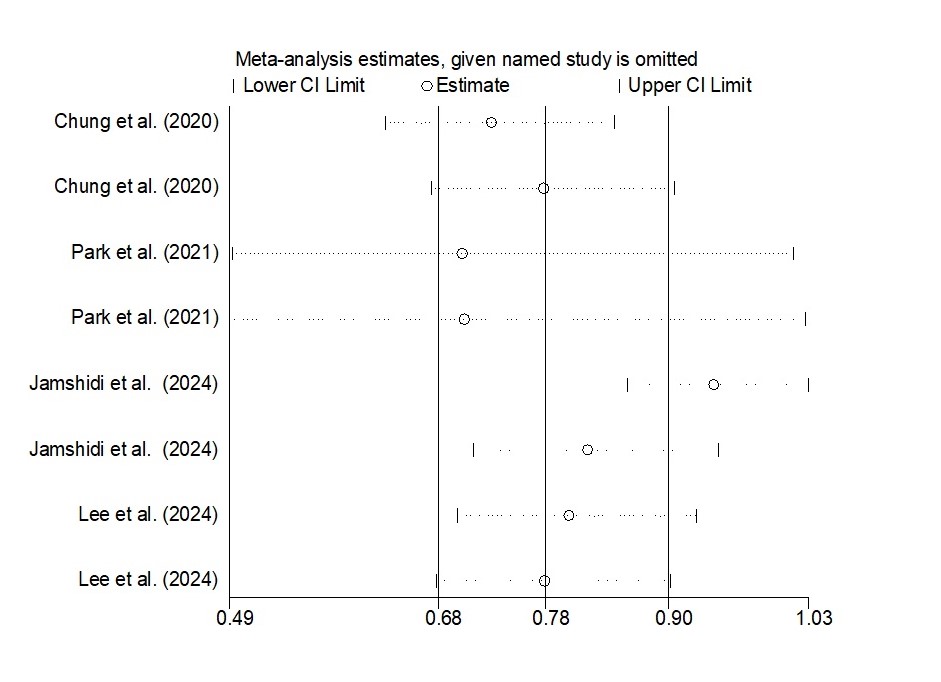


**Supplementary Fig. 30.** The sensitivity analysis for AP and WC


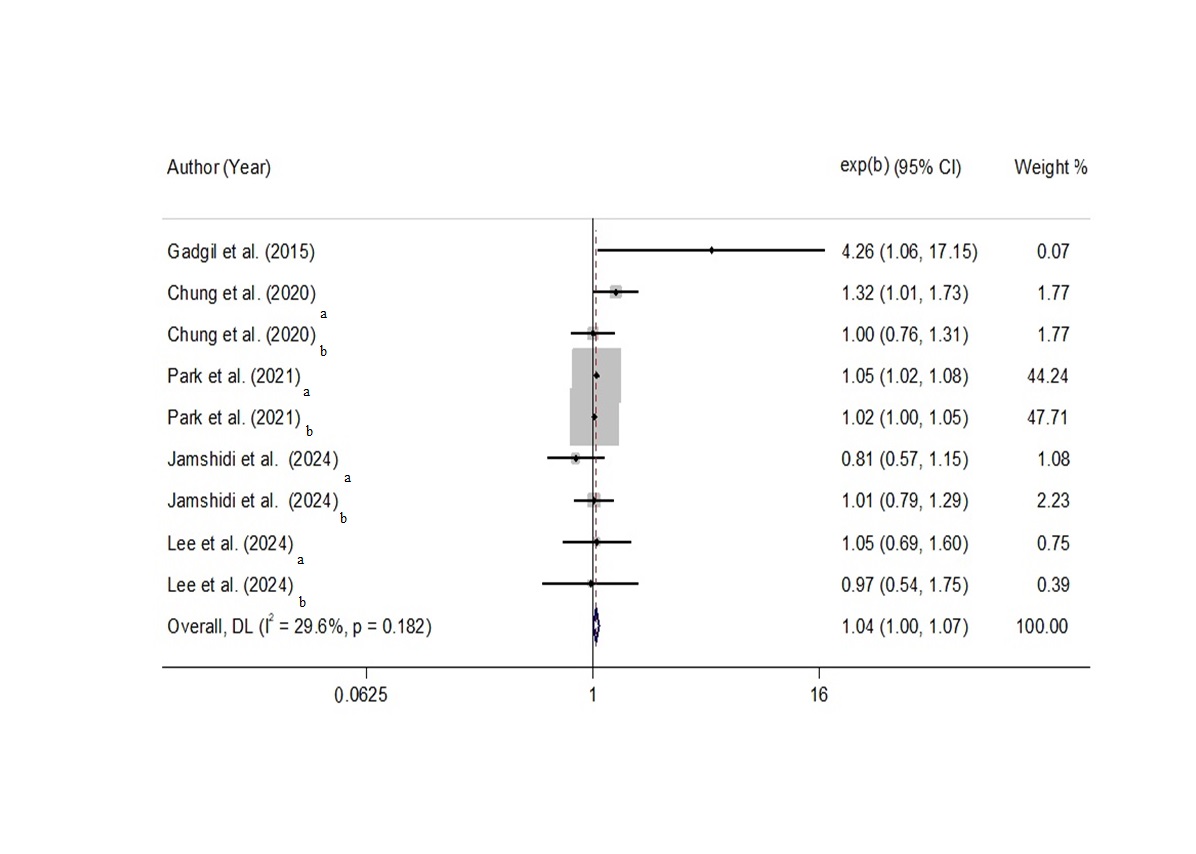


**Supplementary Fig. 31.** The random model for AP and FBS, a: males, b: females


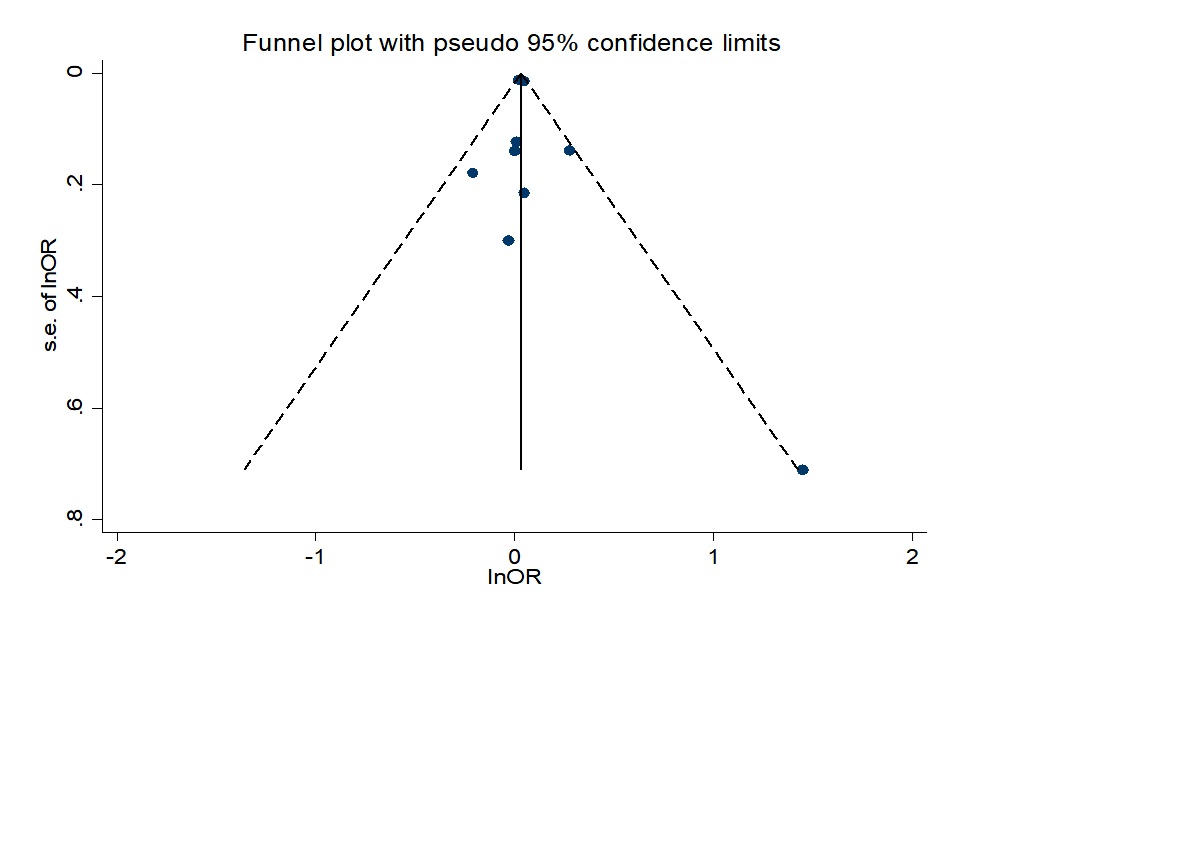


**Supplementary Fig. 32**. The funnel plot for AP and FBS


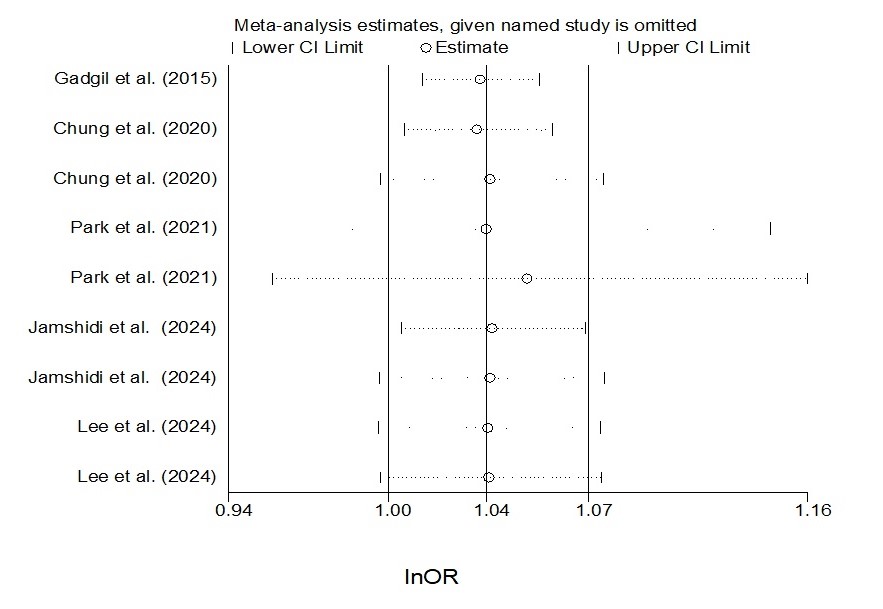


**Supplementary Fig. 33.** The sensitivity analysis for AP and FBS
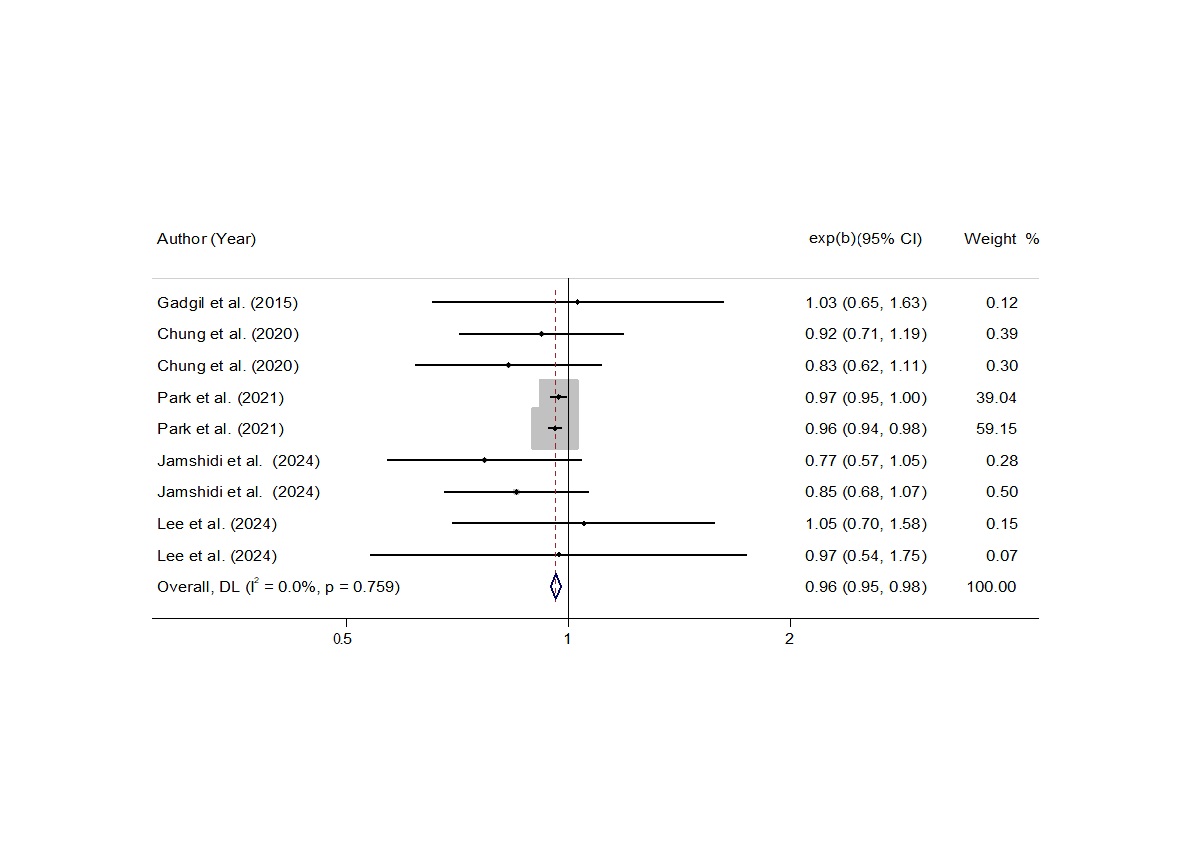


**Supplementary Fig. 34.** A. The random model for AP and BP, a: males, b: females


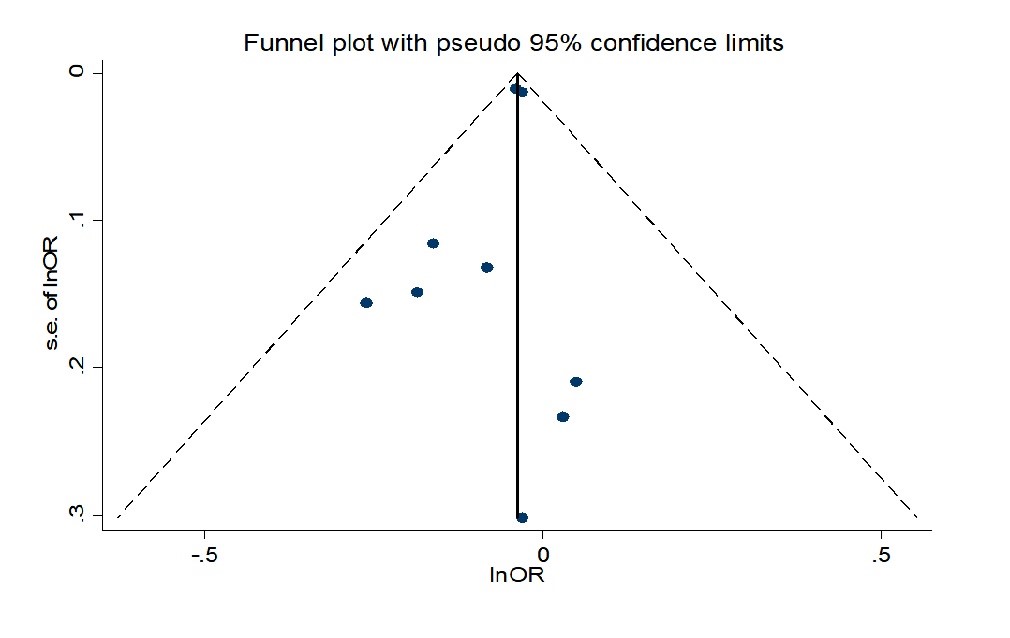


**Supplementary Fig. 35.** The funnel plot for AP and BP


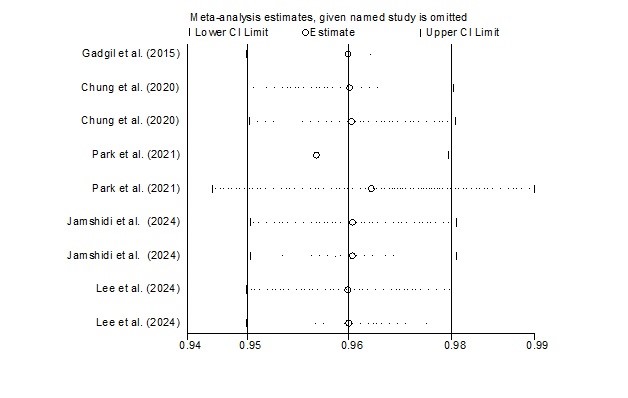


**Supplementary Fig. 36.** The sensitivity analysis for AP and BP


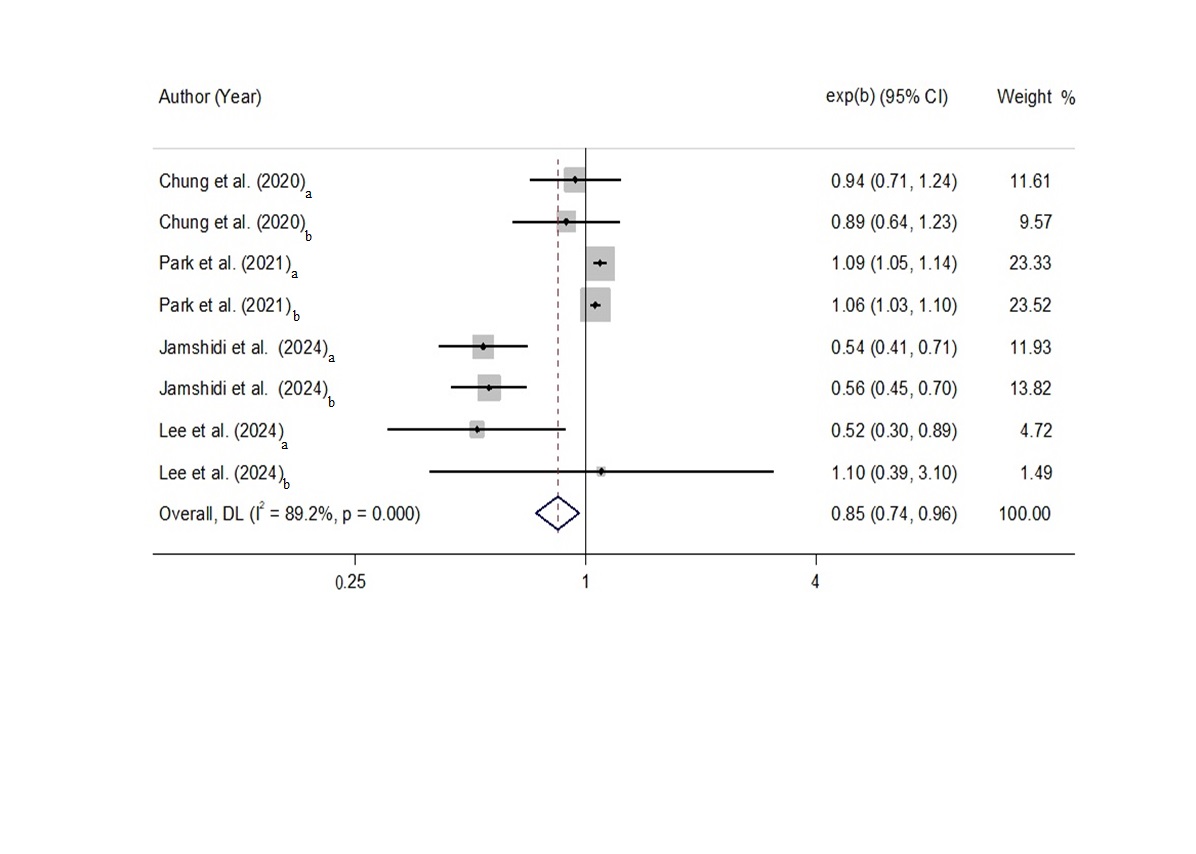


**Supplementary Fig. 37.** The random model for PP and TG, a: males, b: females


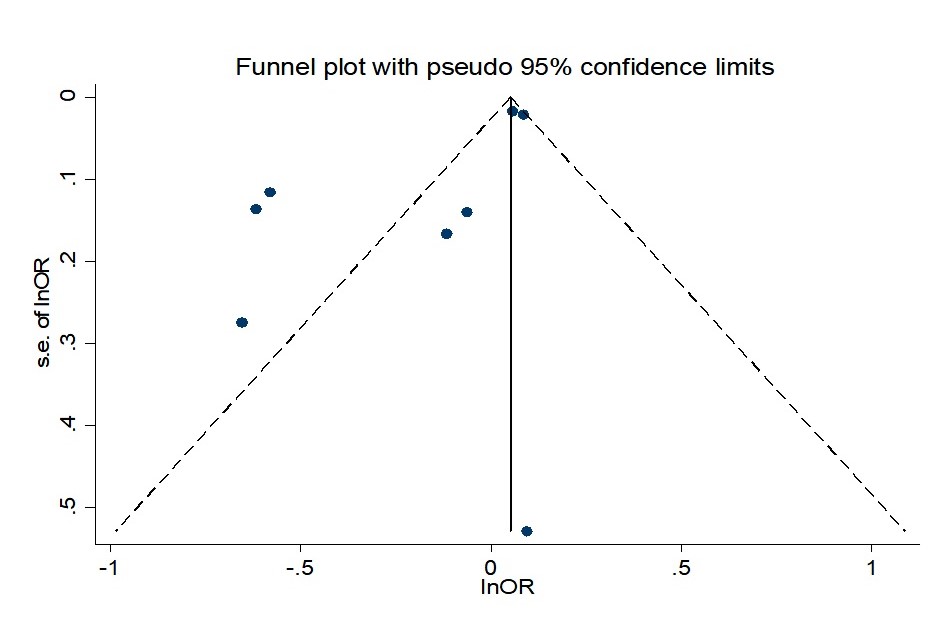


**Supplementary Fig. 38.** The funnel plot for PP and TG


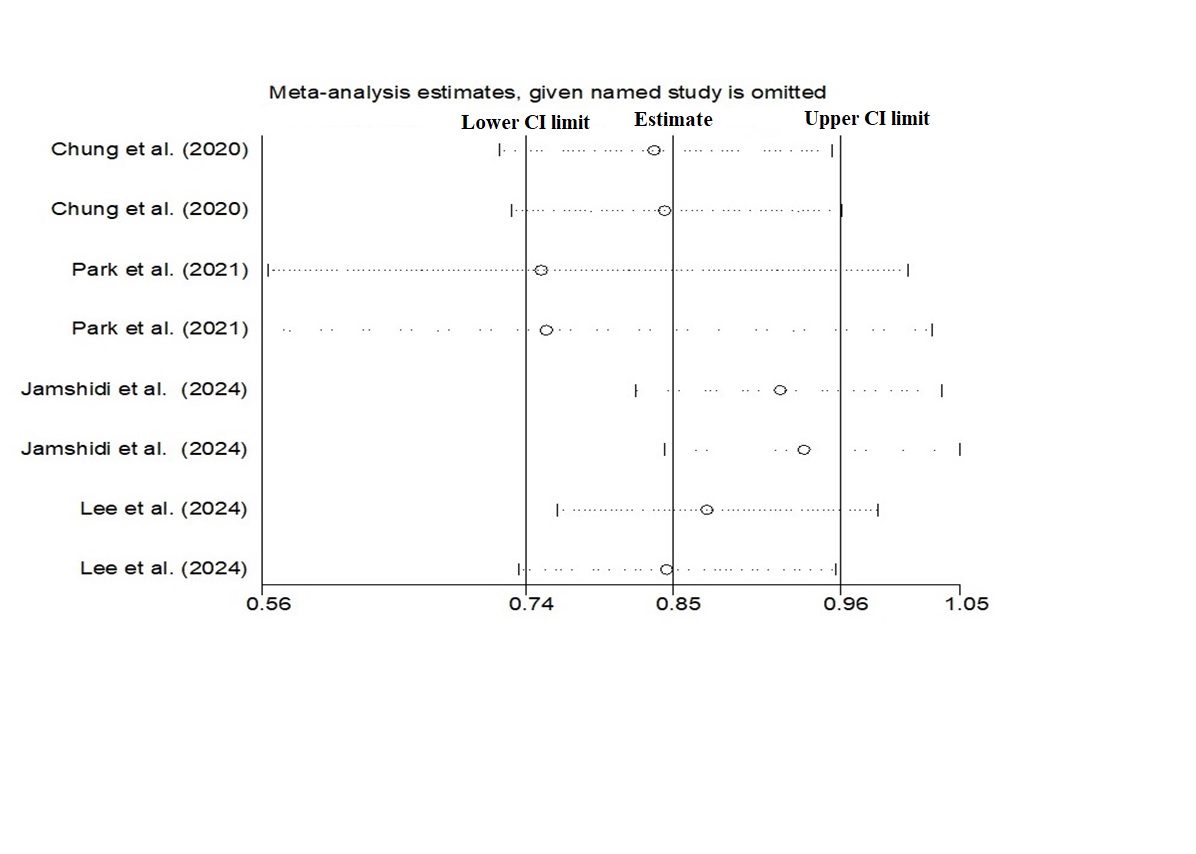


**Supplementary Fig. 39.** The sensitivity analysis for PP and TG


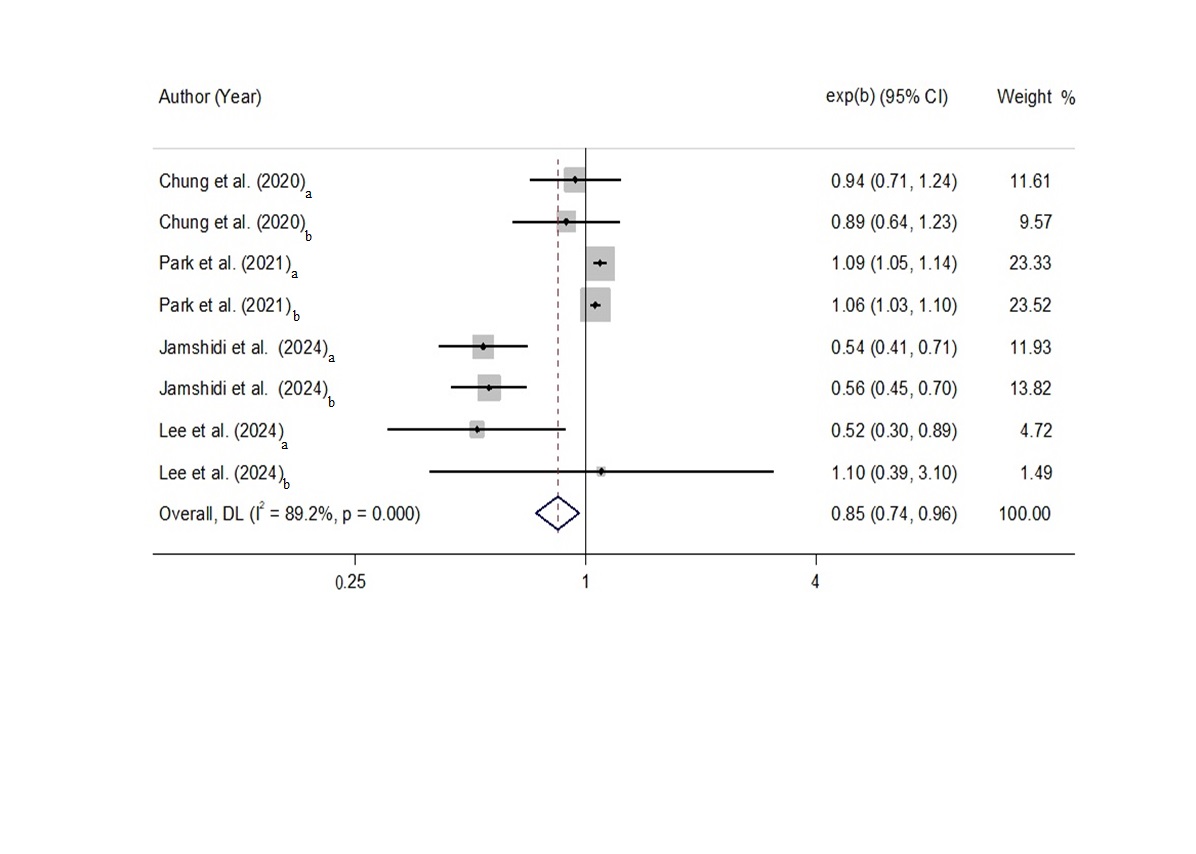


**Supplementary Fig. 40**. The random model for PP and HDL, a: males, b: females


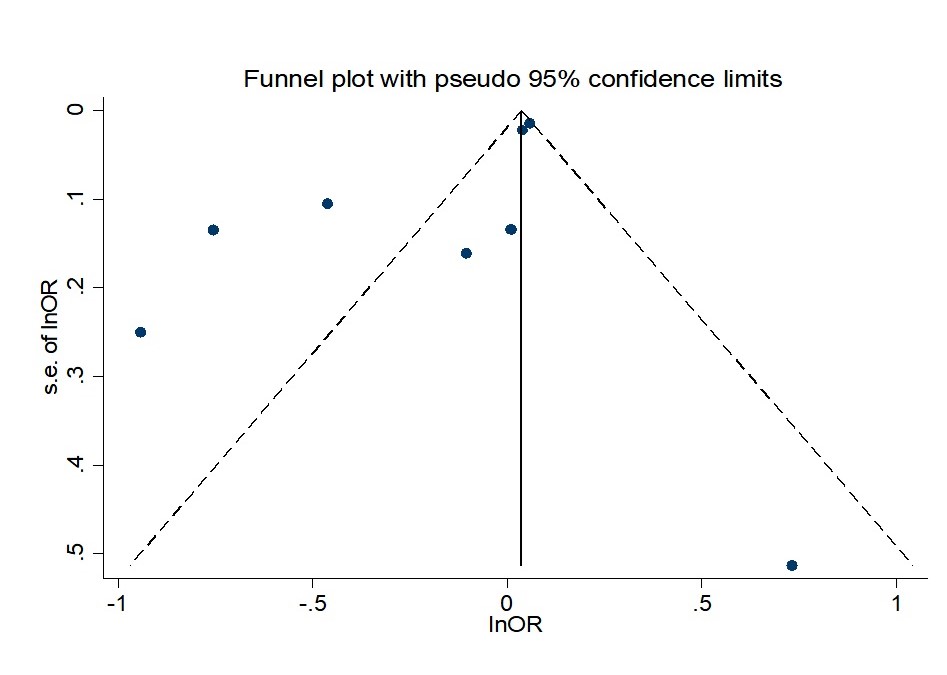


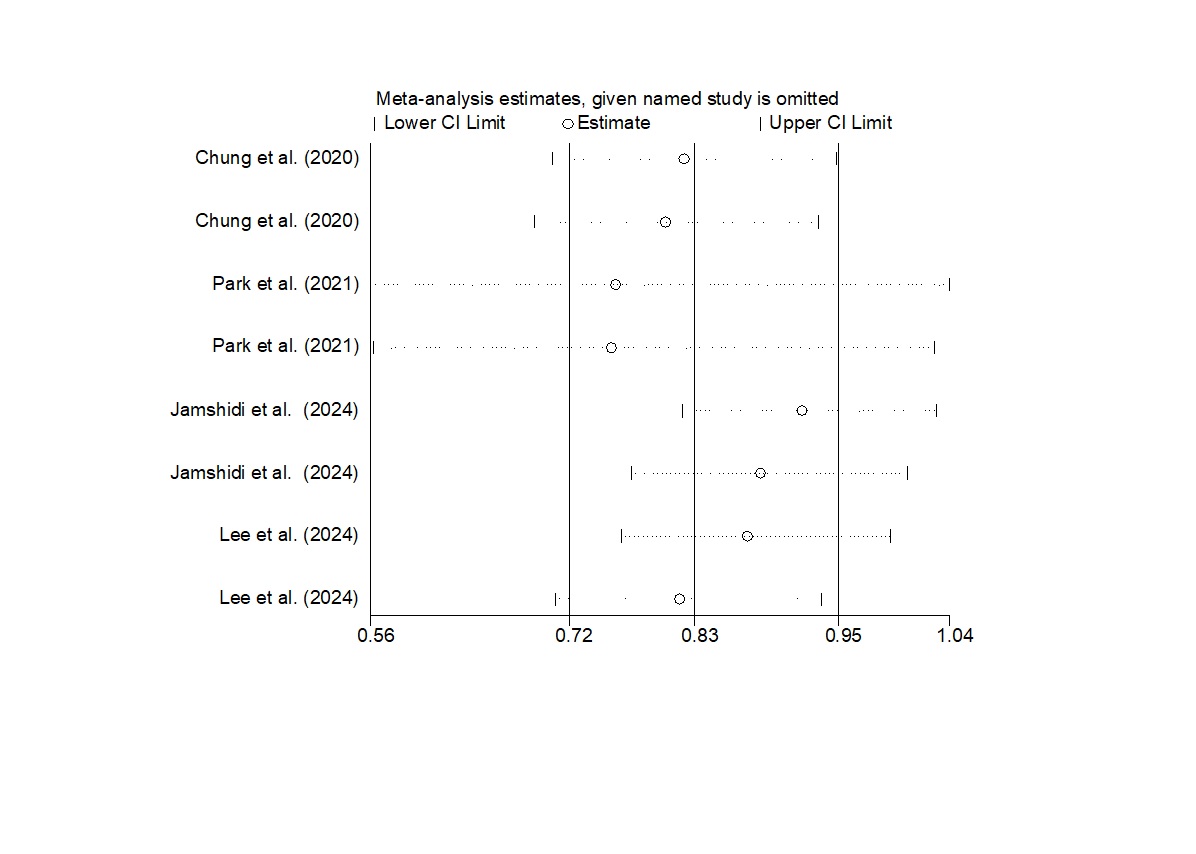
**Supplementary Fig. 41.** The funnel plot for PP and HDL

**Supplementary Fig. 42.** The sensitivity analysis for PP and HDL


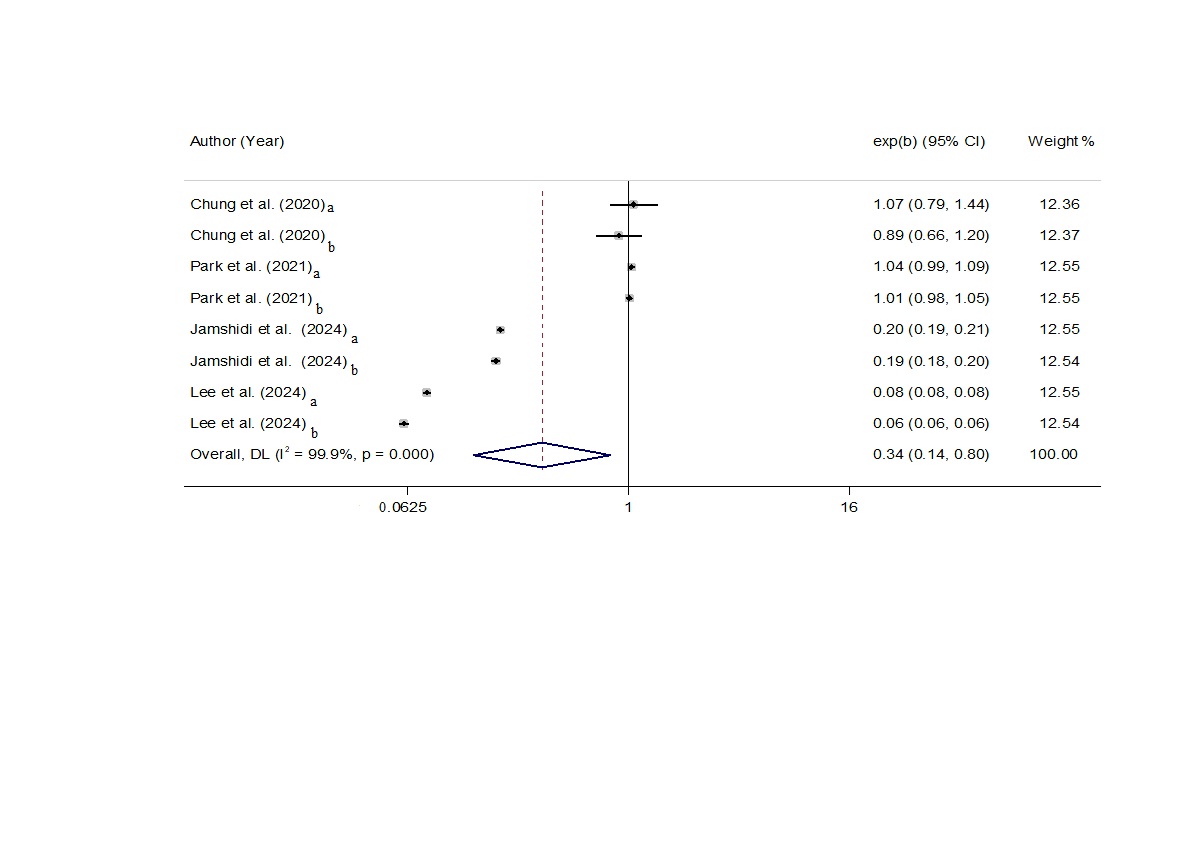
**Supplementary Fig. 43**. The random model for PP and WC, a: males, b: females


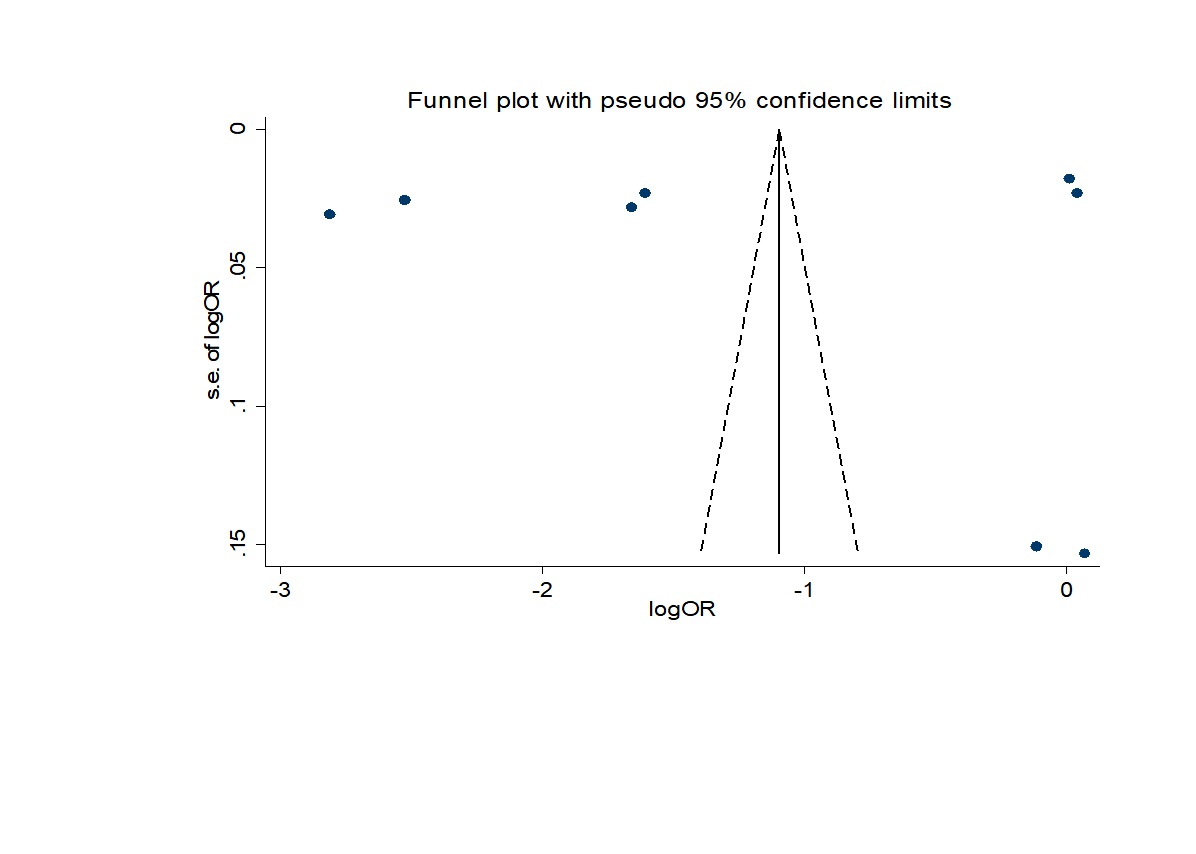


S**upplementary Fig. 44.** The funnel plot for PP and WC

**Supplementary Fig. 45.** The sensitivity analysis for PP and WC


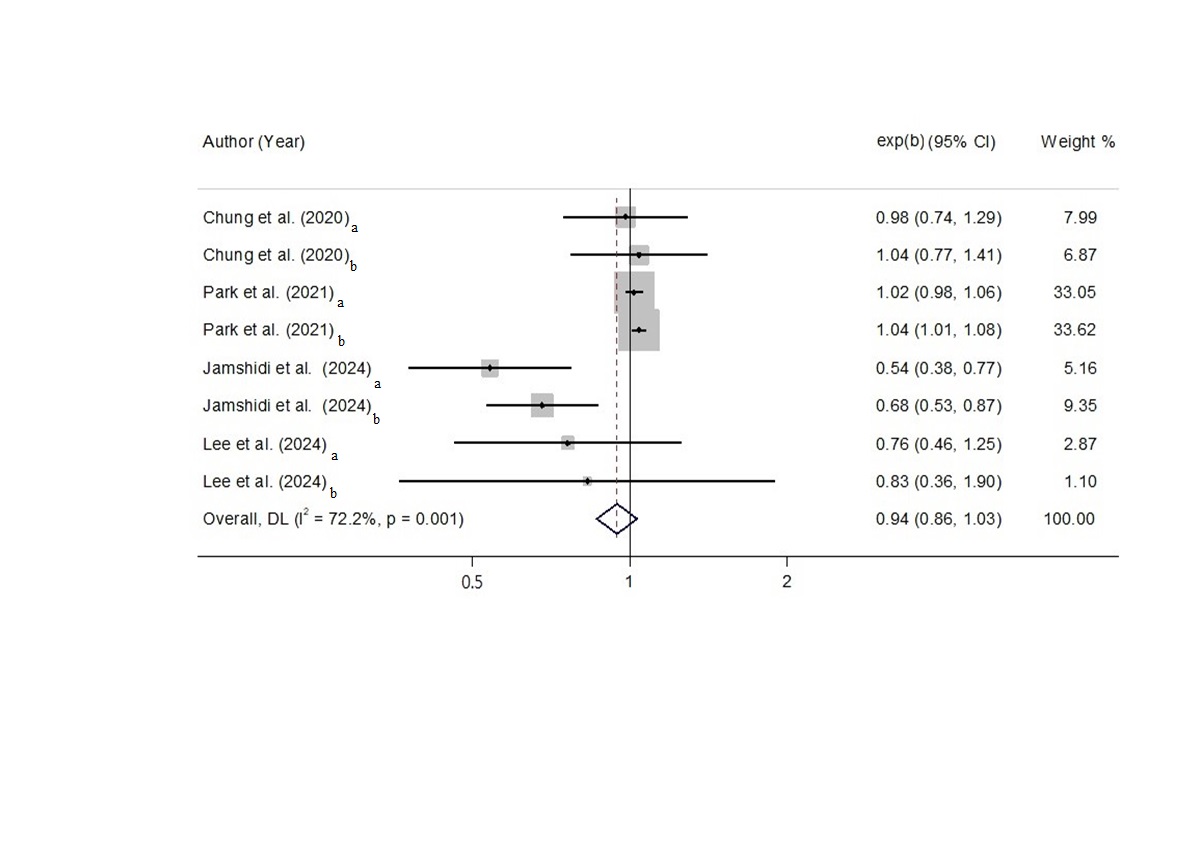


**Supplementary Fig. 46**. The random model for PP and FBS, a: males, b: females


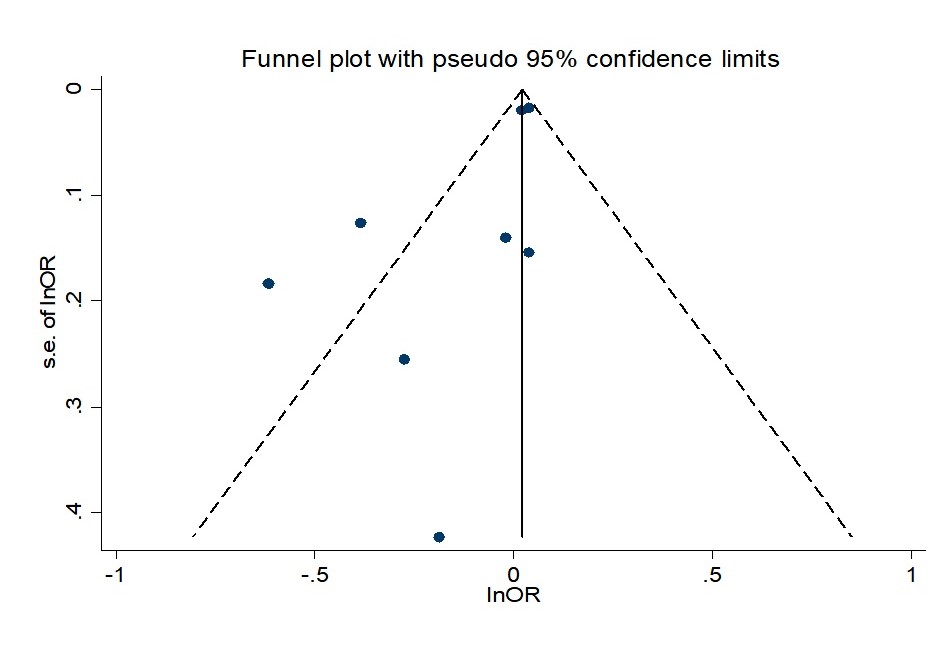


S**upplementary Fig. 47.** The funnel plot for PP and FBS


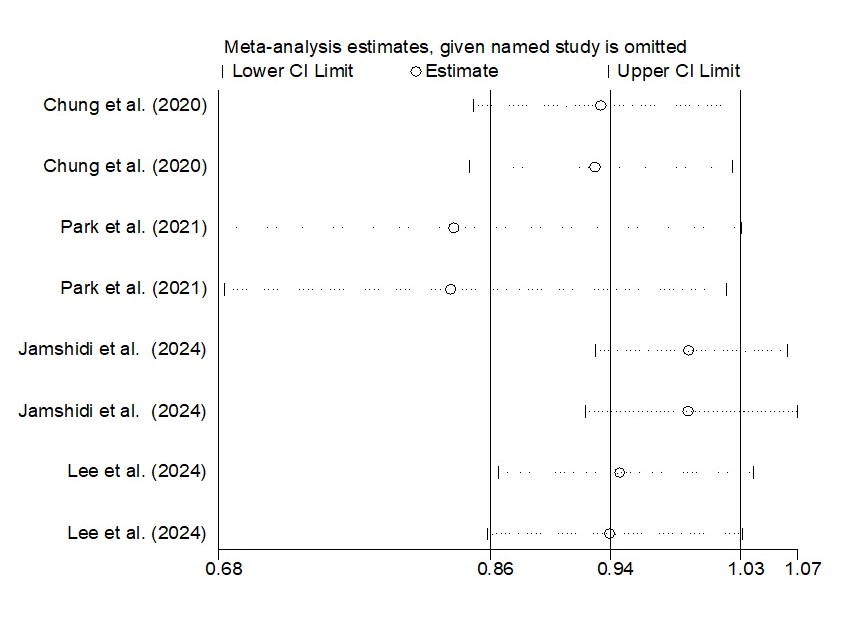


**Supplementary Fig. 48.** The sensitivity analysis for PP and FBS


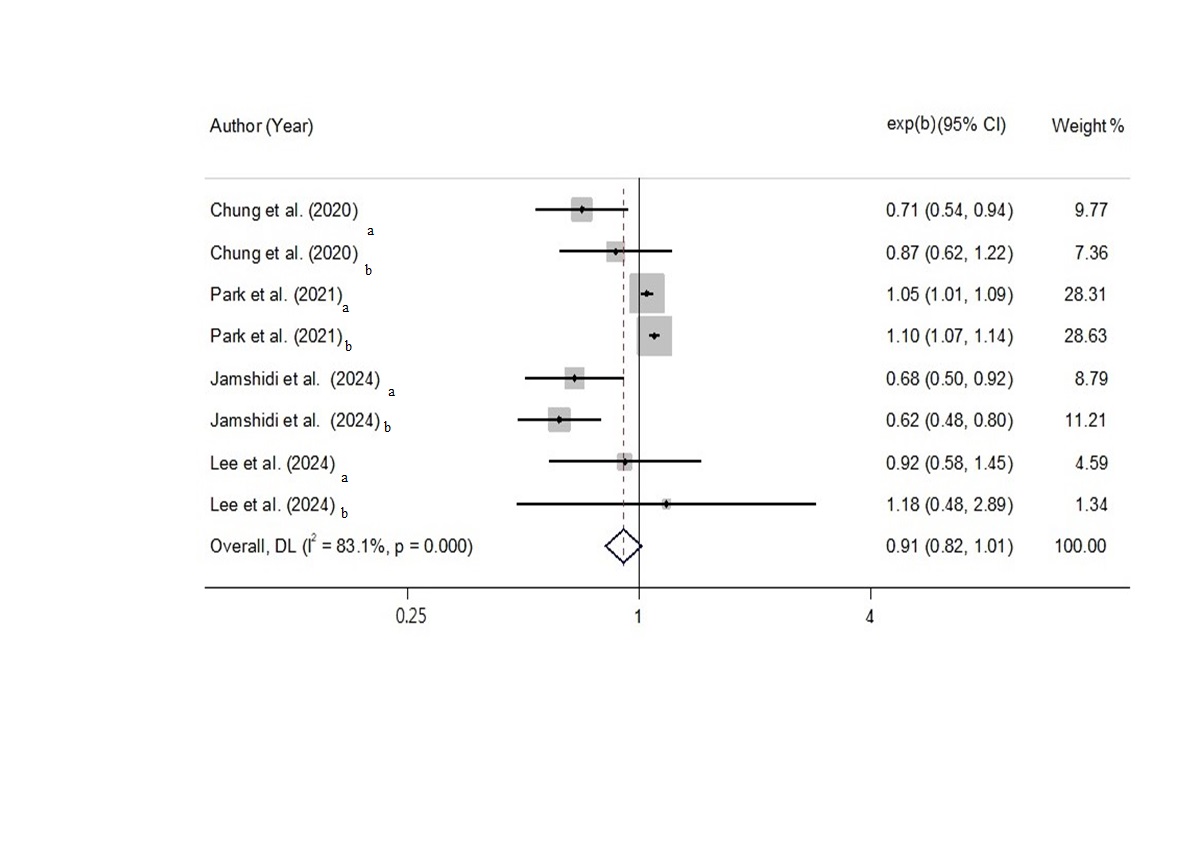


**Supplementary Fig. 49**. The random model for PP and BP, a: males, b: females


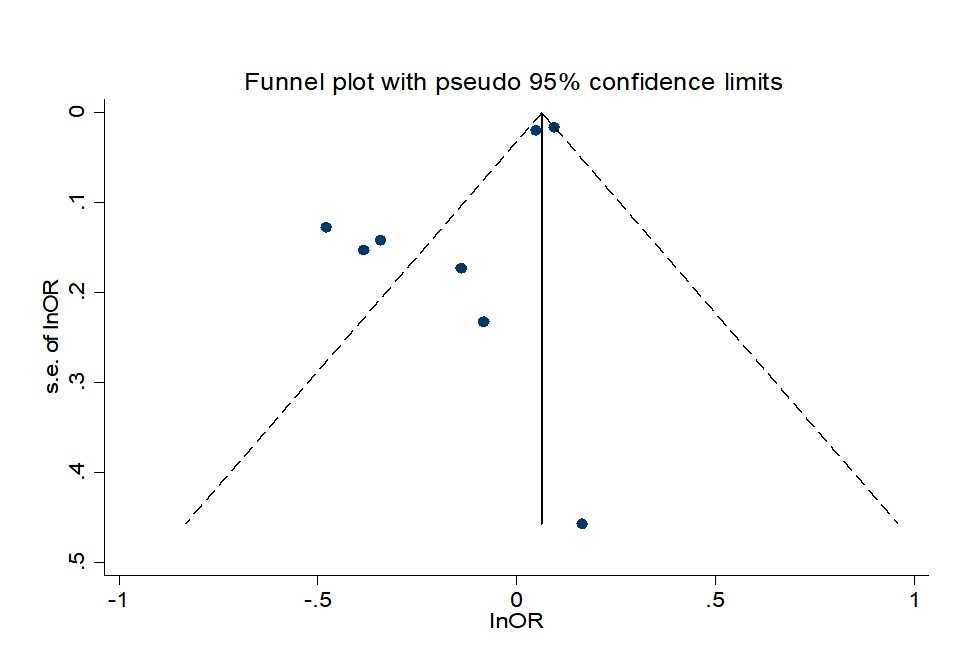


**Supplementary Fig. 50.** The funnel plot for PP and BP


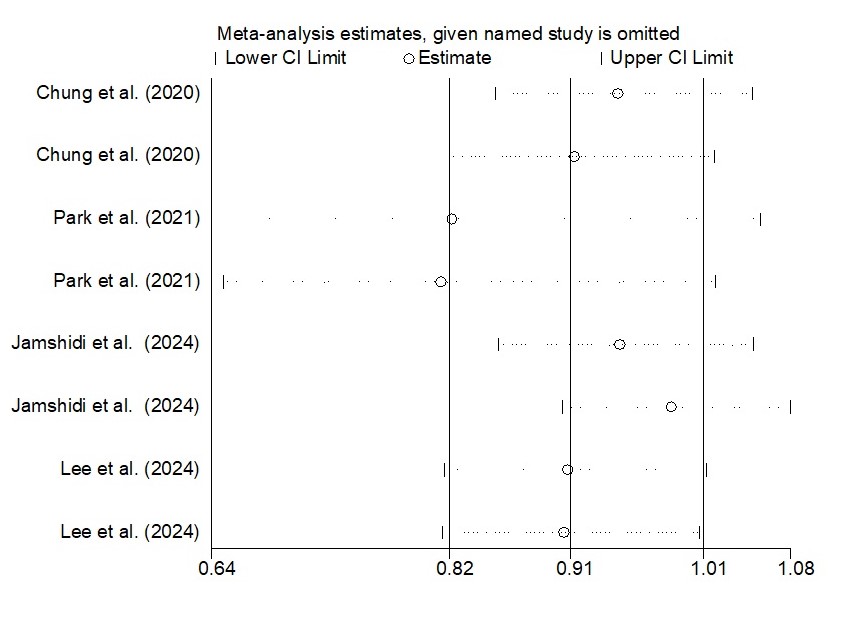
**Supplementary Fig. 51.** The sensitivity analysis for PP and BP
