# Supplementary material for: The association between dietary protein intake and metabolic syndrome: a GRADE-assessed systematic review and meta-analysis of observational studies
Source: Diabetol Metab Syndr. 2025 Dec 29;18:41. doi: 10.1186/s13098-025-02011-0 (PMC12866194; doi:10.1186/s13098-025-02011-0)
Supplement: Supplementary file 2 — Supplementary Material 2 [file 13098_2025_2011_MOESM2_ESM.docx]

**Contents**

**Supplementary Tables for Quality Assessment** 3

**Supplementary Table 1: NOS (Cross-sectional)** 3

**Supplementary Table 2: NOS (COHORT)** 3

**Supplementary Table 3: NOS (CASE CONTROL)** 4

**Supplementary Tables for Primary Outcomes** 4

**Supplementary Table 4**: Subgroup analysis based on Fixed-effects models for the association between dietary TP and Mets 4

**Supplementary Table 5**: Subgroup analysis based on Fixed-effects models for the association between dietary AP and Mets 5

**Supplementary Table 6**: Subgroup analysis based on Fixed-effects models for the association between dietary PP and Mets6

**Supplementary Tables for Secondary Outcomes** 6

**Supplementary Table 7**: Subgroup analysis based on Fixed-effects models for the association between dietary TP and TG7

**Supplementary Table 8**: Subgroup analysis based on Fixed-effects models for the association between dietary TP and HDL7

**Supplementary Table 9**: Subgroup analysis based on Fixed-effects models for the association between dietary TP and WC8

**Supplementary Table 10**: Subgroup analysis based on Fixed-effects models for the association between dietary TP and FBS9

**Supplementary Table 11**: Subgroup analysis based on Fixed-effects models for the association between dietary TP and BP10

**Supplementary Table 12**: Subgroup analysis based on Fixed-effects models for the association between dietary AP and TG10

**Supplementary Table 13**: Subgroup analysis based on Fixed-effects models for the association between dietary AP and HDL11

**Supplementary Table 14**: Subgroup analysis based on Fixed-effects models for the association between dietary AP and WC11

**Supplementary Table 15**: Subgroup analysis based on Fixed-effects models for the association between dietary AP and FBS12

**Supplementary Table 16**: Subgroup analysis based on Fixed-effects models for the association between dietary AP and BP13

**Supplementary Table 17**: Subgroup analysis based on Fixed-effects models for the association between dietary PP and TG14

**Supplementary Table 18**: Subgroup analysis based on Fixed-effects models for the association between dietary PP and HDL14

**Supplementary Table 19**: Subgroup analysis based on Fixed-effects models for the association between dietary PP and WC15

**Supplementary Table 20**: Subgroup analysis based on Fixed-effects models for the association between dietary PP and FBS15

**Supplementary Table 21**: Subgroup analysis based on Fixed-effects models for the association between dietary PP and BP16

**Supplementary Table 1: NOS (Cross-sectional)**

| **First author year**  **Ref.** | **Selection** | | | | **Comparability (based on exposure)** | **Outcome** | | **NOS Score** |
| --- | --- | --- | --- | --- | --- | --- | --- | --- |
|  | **Representativeness**  Sample size> 400 | **Sample size** | **Non-respondents/ excluded** | **Ascertainment of exposure** |  | **Assessment of outcome** | **Statistical test** |  |
| Brunner et al.  2001  30 | ***** | ***** | ***** | ***** | ** | ***** | ***** | **8** |
| Eilat-Adar et al.  2009  21 | ***** | ***** | ***** | ***** | ** | ***** | ***** | **8** |
| Ahola et al. 2017  28 | ***** | ***** | ***** | ***** | ** | ***** | ***** | **8** |
| Nabuco et al. 2018  31 | ***** | ***** | ***** | ***** | * | ***** | ***** | **7** |
| Kim et al.  2019  26 | ***** | ***** | ***** | ***** | ** | ***** | ***** | **8** |
| Azemati et al. 2021  29 | ***** | ***** | ***** | ***** | * | ***** | ***** | **7** |
| Lee et al.  2024  25 | ***** | ***** | ***** | ****** | * | ***** | ***** | **8** |

<http://www.ncbi.nlm.nih.gov/pubmedhealth/PMH0049229/>

**Supplementary Table 2: NOS (COHORT)**

| **First author year**  **Ref.** | **Selection** | | | | **Comparability (based on exposure)** | **Outcome** | | | **NOS Score** |
| --- | --- | --- | --- | --- | --- | --- | --- | --- | --- |
|  | **Representativeness** | **Non-exposed cohort** | **Ascertainment of exposure** | **Outcome at start of study** |  | **Assessment of outcome** | **Follow-up time** | **Follow-up adequacy** |  |
| Gadgil et al. (2015)  23 | ***** | ***** | ***** | ***** | * | * | ***** | ***** | **8** |
| Shang et al.  (2016)  32 | ***** | ***** | ***** | ***** | * | * | ***** | ***** | **8** |
| Chung et al.  2020  16 | ***** | ***** | ***** | ***** | ** |  | ***** | ***** | **8** |
| Vasbinder et al.  2021  24 | ***** | ***** | ***** | ***** | * | * | ***** | ***** | **8** |
| Hajihashemi et al.  2021  33 | ***** | ***** | ***** | ***** | * | * | ***** | ***** | **8** |
| Jamshidi et al. 2024  34 | ***** |  | ***** | ***** | ** | ***** | ***** | ***** | **8** |

| **First author year**  **Ref.** | **Selection** | | | | **Comparability (based on exposure)** | **Exposure** | | | **NOS score** |
| --- | --- | --- | --- | --- | --- | --- | --- | --- | --- |
|  | **Adequate definition** | **Representativeness** | **Selection of controls** | **Definition of controls** |  | **Ascertainment of exposure** | **The same method of ascertainment for cases and controls** | **Non-response rate** |  |
| Park et al.  2021  27 | ***** | ***** |  | ***** | * | ***** | ***** |  | **7** |

**Supplementary Table 3: NOS (CASE CONTROL)**

**Supplementary Table 4**: Subgroup analysis based on Fixed-effects models for the association between dietary TP and Mets

| **Subgroup** | **Effect sizes**  **(n)** | **Effect sizes**  **(95% CI)** | **I^2^**  **(%)** | **P**  **Heterogeneity** | **P**  **Within** | **P**  **Between** |
| --- | --- | --- | --- | --- | --- | --- |
| **Random** | 19 | 0.90 (0.82, 1.00) | 91.3 | < 0.001 | < 0.051 |  |
| **Fix** | 19 | 0.90 (0.88, 0.91) | 91.3 | < 0.001 | < 0.001 |  |
| **Continent** | 19 |  |  |  |  | < 0.001 |
| America | 5 | 0.95 (0.91, 1.00) | 64.3 | 0.024 | 0.058 |  |
| Europe | 4 | 1.28, (1.07, 1.52) | 2.9 | 0.378 | 0.006 |  |
| Asia | 9 | 0.89 (0.87, 0.91) | 95.1 | < 0.001 | < 0.001 |  |
| Australia | 1 | 1.46, (1.01, 2.11) | 0.0 | - | 0.043 |  |
| **Design** | 19 |  |  |  |  | < 0.001 |
| Cohort | 5 | 0.82 (0.79, 0.85) | 96.7 | < 0.001 | < 0.001 |  |
| Cross-sectional | 12 | 0.98 (0.93, 1.03) | 62.8 | 0.002 | 0.335 |  |
| Case-control | 2 | 0.94 (0.91, 0.96) | 88.6 | 0.003 | < 0.001 |  |
| **Gender** | 19 |  |  |  |  | < 0.001 |
| Both | 3 | 0.87 (0.85, 0.90) | 89.7 | < 0.001 | < 0.001 |  |
| Male | 7 | 0.97 (0.93, 1.00) | 93.7 | < 0.001 | 0.078 |  |
| Female | 9 | 0.89 (0.86, 0.93) | 89.3 | 0.003 | < 0.001 |  |
| **Protein assessment** | 19 |  |  |  |  | 0.043 |
| FFQ | 9 | 0.89 (0.87, 0.91) | 95.6 | < 0.001 | < 0.001 |  |
| 24-h dietary recall | 8 | 0.95 (0.91, 1.00) | 58.0 | 0.020 | 0.072 |  |
| Others | 2 | 1.06 (0.70, 1.60) | 0.0 | 0.332 | 0.775 |  |
| **Mets assessment** | 19 |  |  |  |  | < 0.001 |
| Biochemical and anthropometric assessment | 2 | 1.33 (1.10, 1.62) | 16.1 | 0.275 | 0.004 |  |
| ATP III | 14 | 0.92 (0.90, 0.95) | 92.3 | < 0.001 | < 0.001 |  |
| Self-reported | 2 | 1.06 (0.70, 1.60) | 0.0 | 0.332 | 0.775 |  |
| Joint scientific statement | 1 | 0.84; (0.81, 0.87) | 0.0 | - | < 0.001 |  |
| **Age-adjusted** | 19 |  |  |  |  | 0.144 |
| Yes | 15 | 0.90 (0.88, 0.92) | 93.0 | < 0.001 | < 0.001 |  |
| NO | 4 | 1.09 (0.84, 1.42) | 35.7 | - | 0.503 |  |
| **PA-adjusted** | 19 |  |  |  |  | < 0.001 |
| Yes | 8 | 0.85 (0.83, 0.88) | 95.0 | < 0.001 | < 0.001 |  |
| NO | 11 | 0.95 (0.92, 0.97) | 74.4 | < 0.001 | < 0.001 |  |
| **Smoking-adjusted** | 19 |  |  |  |  | 0.002 |
| Yes | 11 | 0.89 (0.87, 0.91) | 94.2 | < 0.001 | < 0.001 |  |
| NO | 8 | 0.97 (0.92, 1.02) | 70.1 | 0.001 | 0.245 |  |
| **BMI-adjusted** | 19 |  |  |  |  | < 0.001 |
| Yes | 7 | 0.89 (0.88, 0.91) | 96.4 | < 0.001 | < 0.001 |  |
| NO | 12 | 1.15 (1.03, 1.29 ) | 41.1 | 0.067 | 0.010 |  |

**Supplementary Table 5**: Subgroup analysis based on Fixed-effects models for the association between dietary AP and Mets

| **Subgroup** | **Effect sizes**  **(n)** | **Effect sizes**  **(95% CI)** | **I^2^**  **(%)** | **P**  **Heterogeneity** | **P**  **Within** | **P**  **Between** |
| --- | --- | --- | --- | --- | --- | --- |
| **Random** | 16 | 0.92 (0.86, 0.98) | 83.5 | < 0.001 | 0.012 |  |
| **Fix** | 16 | 0.92 (0.90, 0.93) | 83.5 | < 0.001 | < 0.001 |  |
| **Continent** | 16 |  |  |  |  | 0.001 |
| America | 5 | 0.96 (0.92, 1.00) | 57.0 | 0.054 | 0.081 |  |
| Asia | 10 | 0.91 (0.89, 0.92) | 86.5 | < 0.001 | < 0.001 |  |
| Australia | 1 | 1.67 (1.13, 2.47) | 0.0 | - | 0.011 |  |
| **Design** | 16 |  |  |  |  | < 0.001 |
| Cohort | 8 | 0.85 (0.82, 0.89) | 87.2 | < 0.001 | < 0.001 |  |
| Cross-sectional | 6 | 0.94 (0.90, 0.99) | 31.7 | 0.198 | 0.025 |  |
| Case-control | 2 | 0.93 (0.91, 0.95) | 90.8 | 0.001 | < 0.001 |  |
| **Gender** |  |  |  |  |  | < 0.001 |
| Male | 4 | 0.97 (0.94, 1.00) | 86.8 | < 0.001 | 0.024 |  |
| Female | 5 | 0.92 (0.90, 0.94) | 67.4 | 0.016 | 0.150 |  |
| Both | 7 | 0.86 (0.83, 0.89) | 80.0 | < 0.001 | < 0.001 |  |
| **Protein assessment** | 16 |  |  |  |  | 0.163 |
| FFQ | 8 | 0.91 (0.90, 0.93) | 90.8 | < 0.001 | < 0.001 |  |
| 24-h dietary recall | 8 | 0.95 (0.90, 1.00) | 43.3 | 0.090 | 0.038 |  |
| **Mets assessment** | 16 |  |  |  |  | < 0.001 |
| ATP III | 14 | 0.93 (0.92, 0.95) | 75.2 | < 0.001 | < 0.001 |  |
| Joint scientific statement | 1 | 0.81 (0.78, 0.85) | 0.0 | - | < 0.001 |  |
| Biochemical and anthropometric | 1 | 0.69 (0.43, 1.10) | 0.0 | - | 0.121 |  |
| **Age-adjusted** | 16 |  |  |  |  | 0.266 |
| Yes | 14 | 0.91 (0.90, 0.93) | 85.4 | < 0.001 | < 0.001 |  |
| NO | 2 | 0.91 (0.90, 0.93) | 5.2 | 0.304 | 0.240 |  |
| **PA-adjusted** | 16 |  |  |  |  | < 0.002 |
| Yes | 10 | 0.88 (0.86, 0.91) | 85.7 | < 0.001 | < 0.001 |  |
| NO | 6 | 0.93 (0.91, 0.95) | 72.6 | 0.003 | < 0.001 |  |
| **Smoking-adjusted** | 16 |  |  |  |  | 0.405 |
| Yes | 13 | 0.92 (0.90, 0.93 ) | 86.4 | < 0.001 | < 0.001 |  |
| NO | 3 | 0.94 (0.89, 0.99) | 0.0 | 0.437 | 0.015 |  |
| **BMI-adjusted** | 16 |  |  |  |  | 0.005 |
| Yes | 8 | 0.91 (0.90, 0.93) | 90.2 | < 0.001 | < 0.001 |  |
| NO | 8 | 1.01 (0.94, 1.08) | 38.7 | 0.122 | 0.755 |  |

**Supplementary Table 6**: Subgroup analysis based on Fixed-effects models for the association between dietary PP and Mets

| **Subgroup** | **Effect sizes**  **(n)** | **Effect sizes**  **(95% CI)** | **I^2^**  **(%)** | **P**  **Heterogeneity** | **P**  **Within** | **P**  **Between** |
| --- | --- | --- | --- | --- | --- | --- |
| **Random** | 14 | 0.77 (0.69, 0.87) | 93.0 | < 0.001 | < 0.001 |  |
| **Fix** | 14 | 1.01 (0.99, 1.03) | 93.0 | < 0.001 | 0.447 |  |
| **Continent** | 14 |  |  |  |  | 0.028 |
| Asia | 9 | 1.02 (0.99, 1.05) | 95.5 | < 0.001 | 0.123 |  |
| America | 4 | 0.98 (0.93, 1.02) | 18.2 | 0.300 | 0.308 |  |
| Australia | 1 | 0.60 (0.37, 0.97) | 0.0 | - | 0.038 |  |
| **Design** | 14 |  |  |  |  | < 0.001 |
| Cohort | 7 | 0.88 (0.84, 0.92) | 93.9 | < 0.001 | < 0.001 |  |
| Cross-sectional | 5 | 0.89 (0.81, 0.98) | 85.5 | < 0.001 | 0.018 |  |
| Case-control | 2 | 1.07 (1.04, 1.10) | 41.0 | 0.193 | < 0.001 |  |
| **Gender** | 14 |  |  |  |  | < 0.001 |
| Male | 4 | 1.02 (0.98, 1.07) | 93.6 | < 0.001 | 0.265 |  |
| Female | 5 | 1.04 (1.01, 1.07) | 94.6 | < 0.001 | 0.011 |  |
| Both | 5 | 0.83 (0.78, 0.89) | 86.7 | < 0.001 | < 0.001 |  |
| **Protein assessment** | 14 |  |  |  |  | 0.005 |
| FFQ | 7 | 1.02 (0.99, 1.04) | 96.0 | < 0.001 | 0.138 |  |
| 24-h dietary recall | 7 | 0.90 (0.82, 0.98) | 78.3 | < 0.001 | 0.012 |  |
| **Mets assessment** | 14 |  |  |  |  | < 0.001 |
| ATP III | 13 | 1.02 (1.00, 1.05) | 92.5 | < 0.001 | 0.045 |  |
| Joint scientific statement | 1 | 0.79 (0.72, 0.87) | 0.0 | - | < 0.001 |  |
| **PA-adjusted** | 14 |  |  |  |  | < 0.001 |
| Yes | 10 | 0.89 (0.85, 0.92) | 92.8 | < 0.001 | <0.001 |  |
| NO | 4 | 1.07 (1.04, 1.10) | 49.2 | 0.116 | <0.001 |  |
| **Smoking-adjusted** | 14 |  |  |  |  | 0.158 |
| Yes | 13 | 1.01 (0.99, 1.04) | 93.5 | < 0.001 | 0.288 |  |
| NO | 1 | 0.94 (0.85, 1.04) | 0.0 | - | 0.229 |  |
| **BMI-adjusted** | 14 |  |  |  |  | 0.080 |
| Yes | 7 | 1.02 (0.99, 1.05) | 96.1 | < 0.001 | 0.147 |  |
| NO | 7 | 0.97 (0.92, 1.02) | 80.7 | < 0.001 | 0.214 |  |

All studies were adjusted for age

**Supplementary Table 7**: Subgroup analysis based on Fixed-effects models for the association between dietary TP and TG

| **Subgroup** | **Effect sizes**  **(n)** | **Effect sizes**  **(95% CI)** | **I^2^**  **(%)** | **P**  **Heterogeneity** | **P**  **Within** | **P**  **Between** |
| --- | --- | --- | --- | --- | --- | --- |
| **Random** | 7 | 0.80 (0.70, 0.91) | 89.5 | <0.001 | 0.001 |  |
| **Fix** | 7 | 0.92 (0.90, 0.95) | 89.5 | <0.001 | <0.001 |  |
| **Continent** | 7 |  |  |  |  | 0.504 |
| America | 1 | 1.62 (0.63, 4.19) | 0.0 | - | 0.320 |  |
| Europe | 2 | 0.89 (0.60, 1.33) | 0.0 | 0.599 | 0.573 |  |
| Asia | 4 | 0.92 (0.90, 0.95) | 94.6 | < 0.001 | < 0.001 |  |
| **Design** | 7 |  |  |  |  | < 0.001 |
| Cohort | 2 | 0.57 (0.49, 0.66) | 78.4 | 0.031 | < 0.001 |  |
| Cross-sectional | 3 | 0.98 (0.67, 1.41) | 0.0 | 0.457 | 0.896 |  |
| Case-control | 2 | 0.94 (0.91, 0.96) | 91.4 | 0.001 | < 0.001 |  |
| **Gender** | 7 |  |  |  |  | 0.002 |
| Male | 3 | 0.96 (0.93, 1.00) | 94.6 | < 0.001 | 0.040 |  |
| Female | 4 | 0.89 (0.86, 0.92) | 71.2 | 0.015 | < 0.001 |  |
| **Protein assessment** | 7 |  |  |  |  | 0.504 |
| FFQ | 4 | 0.92 (0.90, 0.95) | 94.6 | < 0.001 | < 0.001 |  |
| 24-h dietary recall | 1 | 1.62 (0.63, 4.19) | 0.0 | - | 0.320 |  |
| Others | 2 | 0.89 (0.60, 1.33) | 0.0 | 0.599 | 0.573 |  |
| **Mets assessment** | 7 |  |  |  |  | 0.854 |
| ATP III | 5 | 0.93 (0.90, 0.95) | 93.0 | < 0.001 | < 0.001 |  |
| Self-reported | 2 | 0.89 (0.60, 1.33) | 0.0 | 0.599 | 0.573 |  |
| **PA-adjusted** | 7 |  |  |  |  | < 0.001 |
| Yes | 4 | 0.60 (0.52, 0.70) | 66.8 | 0.029 | < 0.001 |  |
| NO | 3 | 0.94 (0.91, 0.96) | 84.5 | 0.002 | < 0.001 |  |
| **Smoking-adjusted** | 7 |  |  |  |  | 0.248 |
| Yes | 6 | 0.92 (0.90, 0.95) | 91.0 | < 0.001 | < 0.001 |  |
| NO | 1 | 1.62 (0.63, 4.19) | 0.0 | - | 0.320 |  |
| **BMI-adjusted** | 7 |  |  |  |  | 0.778 |
| Yes | 4 | 0.92 (0.90, 0.95) | 94.6 | < 0.001 | < 0.001 |  |
| NO | 3 | 0.98 (0.67, 1.41 ) | 0.0 | 0.457 | 0.896 |  |

All studies were adjusted for age

**Supplementary Table 8**: Subgroup analysis based on Fixed-effects models for the association between dietary TP and HDL

| **Subgroup** | **Effect sizes**  **(n)** | **Effect sizes**  **(95% CI)** | **I^2^**  **(%)** | **P**  **Heterogeneity** | **P**  **Within** | **P**  **Between** |
| --- | --- | --- | --- | --- | --- | --- |
| **Random** | 7 | 0.80 (0.72, 0.89) | 85.6 | <0.001 | <0.001 |  |
| **Fix** | 7 | 0.89 (0.87, 0.91) | 85.6 | <0.001 | <0.001 |  |
| **Continent** | 7 |  |  |  |  | 0.365 |
| America | 1 | 1.32 (0.64, 2.74) | 0.0 | - | 0.455 |  |
| Europe | 2 | 1.08 (0.72, 1.61) | 53.2 | 0.144 | 0.720 |  |
| Asia | 4 | 0.89 (0.87, 0.91) | 92.0 | < 0.001 | < 0.001 |  |
| **Design** | 7 |  |  |  |  | < 0.001 |
| Cohort | 2 | 0.60 (0.52, 0.69) | 62.1 | 0.104 | < 0.001 |  |
| Cross-sectional | 3 | 1.13 (0.79, 1.60) | 15.5 | 0.306 | 0.501 |  |
| Case-control | 2 | 0.90 (0.88, 0.92) | 69.0 | 0.072 | < 0.001 |  |
| **Gender** | 7 |  |  |  |  | 0.043 |
| Male | 3 | 0.86 (0.83, 0.89) | 91.1 | < 0.001 | < 0.001 |  |
| Female | 4 | 0.90 (0.88, 0.93) | 80.1 | 0.002 | < 0.001 |  |
| **Protein assessment** | 7 |  |  |  |  | 0.365 |
| FFQ | 4 | 0.89 (0.87, 0.91) | 92.0 | < 0.001 | < 0.001 |  |
| 24-h dietary recall | 1 | 1.32 (0.64, 2.74) | 0.0 | - | 0.455 |  |
| Diet 3.2 software | 2 | 1.08 (0.72, 1.61) | 53.2 | 0.144 | 0.720 |  |
| **Mets assessment** | 7 |  |  |  |  | 0.348 |
| ATP III | 5 | 0.89 (0.87, 0.91) | 89.7 | < 0.001 | < 0.001 |  |
| Self-reported | 2 | 1.08 (0.72, 1.61) | 53.2 | 0.144 | 0.720 |  |
| **PA-adjusted** | 7 |  |  |  |  | < 0.001 |
| Yes | 4 | 0.64 (0.56, 0.73) | 75.4 | 0.007 | < 0.001 |  |
| NO | 3 | 0.90 (0.88, 0.92) | 53.6 | 0.116 | < 0.001 |  |
| **Smoking-adjusted** | 7 |  |  |  |  | 0.287 |
| Yes | 6 | 0.89 (0.87, 0.91) | 87.7 | < 0.001 | < 0.001 |  |
| NO | 1 | 1.32 (0.64, 2.74) | 0.0 | - | 0.455 |  |
| **BMI-adjusted** | 7 |  |  |  |  | 0.181 |
| Yes | 4 | 0.89 (0.87, 0.91) | 92.0 | < 0.001 | < 0.001 |  |
| NO | 3 | 1.13 (0.79, 1.60 ) | 15.5 | 0.306 | 0.501 |  |

All studies were adjusted for age

**Supplementary Table 9**: Subgroup analysis based on Fixed-effects models for the association between dietary TP and WC

| **Subgroup** | **Effect sizes**  **(n)** | **Effect sizes**  **(95% CI)** | **I^2^**  **(%)** | **P**  **Heterogeneity** | **P**  **Within** | **P**  **Between** |
| --- | --- | --- | --- | --- | --- | --- |
| **Random** | 7 | 0.78 (0.55, 1.12) | 80.0 | <0.001 | 0.178 |  |
| **Fix** | 7 | 1.01 (0.91, 1.12) | 80.0 | <0.001 | 0.890 |  |
| **Continent** | 7 |  |  |  |  |  |
| America | 1 | 4.81 (0.17, 138.53) | 0.0 | - | 0.360 | 0250 |
| Europe | 2 | 1.19 (0.92, 1.55) | 0.0 | 0.924 | 0.186 |  |
| Asia | 4 | 0.97 (0.87, 1.09) | 89.0 | < 0.001 | 0.656 |  |
| **Design** | 7 |  |  |  |  | < 0.001 |
| Cohort | 2 | 0.13 (0.06, 0.28) | 0.0 | 0.777 | < 0.001 |  |
| Cross-sectional | 3 | 1.20 (0.93, 1.56) | 0.0 | 0.717 | 0.164 |  |
| Case-control | 2 | 1.02 (0.91, 1.14) | 0.0 | 0.816 | 0.745 |  |
| **Gender** | 7 |  |  |  |  | 0.553 |
| Male | 3 | 0.95 (0.78, 1.17) | 88.3 | < 0.001 | 0.641 |  |
| Female | 4 | 1.03 (0.91, 1.16) | 76.0 | 0.006 | 0.662 |  |
| **Protein assessment** | 7 |  |  |  |  | 0.250 |
| FFQ | 4 | 0.97 (0.87, 1.09) | 89.0 | < 0.001 | 0.656 |  |
| 24-h dietary recall | 1 | 4.81 (0.17, 138.53) | 0.0 | - | 0.360 |  |
| Others | 2 | 1.19 ( 0.92, 1.56) | 0.0 | 0.924 | 0.186 |  |
| **Mets assessment** | 7 |  |  |  |  | 0.168 |
| ATP III | 5 | 0.98 (0.87, 1.09) | 85.5 | < 0.001 | 0.678 |  |
| Self-reported | 2 | 1.19 (0.92, 1.55) | 0.0 | 0.924 | 0.176 |  |
| **PA-adjusted** | 7 |  |  |  |  | 0.581 |
| Yes | 4 | 0.95 (0.74, 1.21) | 89.6 | < 0.001 | 0.657 |  |
| NO | 3 | 1.02 (0.91, 1.14) | 0.0 | 0.646 | 0.722 |  |
| **Smoking-adjusted** | 7 |  |  |  |  | 0.362 |
| Yes | 6 | 1.01 (0.91, 1.11) | 82.9 | < 0.001 | 0.912 |  |
| NO | 1 | 4.81 (0.17, 138.53) | 0.0 | - | 0.360 |  |
| **BMI-adjusted** | 7 |  |  |  |  | 0.146 |
| Yes | 4 | 0.97 (0.87, 1.09) | 89.0 | < 0.001 | 0.656 |  |
| NO | 3 | 1.20 (0.93, 1.56 ) | 0.0 | 0.717 | 0.164 |  |

All studies were adjusted for age

**Supplementary Table 10**: Subgroup analysis based on Fixed-effects models for the association between dietary TP and FBS

| **Subgroup** | **Effect sizes**  **(n)** | **Effect sizes**  **(95% CI)** | **I^2^**  **(%)** | **P**  **Heterogeneity** | **P**  **Within** | **P**  **Between** |
| --- | --- | --- | --- | --- | --- | --- |
| **Random** | 5 | 0.93 (0.82, 1.05) | 91.0 | <0.001 | 0.231 |  |
| **Fix** | 5 | 1.05 (1.02, 1.07) | 91.0 | <0.001 | <0.001 |  |
| **Continent** | 5 |  |  |  |  | 0.009 |
| America | 1 | 2.89 (1.35, 6.17) | 0.0 | - | 0.006 |  |
| Asia | 4 | 1.05 (1.02, 1.07) | 92.0 | < 0.001 | <0.001 |  |
| **Design** | 5 |  |  |  |  | < 0.001 |
| Cohort | 2 | 0.61 (0.51, 0.73) | 31.4 | 0.227 | < 0.001 |  |
| Cross-sectional | 1 | 2.89 (1.35, 6.17) | 0.0 | - | 0.006 |  |
| Case-control | 2 | 1.06 (1.03, 1.08) | 29.7 | 0.233 | < 0.001 |  |
| **Gender** | 5 |  |  |  |  | 0.211 |
| Male | 2 | 1.06 (1.03, 1.10) | 94.8 | < 0.001 | < 0.001 |  |
| Female | 3 | 1.03 (1.00, 1.07) | 91.5 | < 0.001 | 0.069 |  |
| **Protein assessment** | 5 |  |  |  |  | 0.009 |
| FFQ | 4 | 1.05 (1.02, 1.07) | 92.0 | < 0.001 | < 0.001 |  |
| 24-h dietary recall | 1 | 2.89 (1.35, 6.17) | 0.0 | - | 0.006 |  |
| **PA-adjusted** | 5 |  |  |  |  | < 0.001 |
| Yes | 2 | 0.61 (0.51, 0.73) | 31.4 | 0.227 | < 0.001 |  |
| NO | 3 | 1.06 (1.03, 1.08) | 75.6 | 0.017 | < 0.001 |  |
| **Smoking-adjusted** | 5 |  |  |  |  | 0.009 |
| Yes | 4 | 1.05 (1.02, 1.07) | 92.0 | < 0.001 | 0.912 |  |
| NO | 1 | 2.89 (1.35, 6.17) | 0.0 | - | 0.006 |  |
| **BMI-adjusted** | 5 |  |  |  |  | 0.009 |
| Yes | 4 | 1.05 (1.02, 1.07) | 92.0 | < 0.001 | < 0.001 |  |
| NO | 1 | 2.89 (1.35, 6.17 ) | 0.0 | - | 0.006 |  |

All studies were adjusted for age, and applied the ATP III as a type of MetS assessment

**Supplementary Table 11**: Subgroup analysis based on Fixed-effects models for the association between dietary TP and BP

| **Subgroup** | **Effect sizes**  **(n)** | **Effect sizes**  **(95% CI)** | **I^2^**  **(%)** | **P**  **Heterogeneity** | **P**  **Within** | **P**  **Between** |
| --- | --- | --- | --- | --- | --- | --- |
| **Random** | 7 | 0.86 (0.76, 0.96) | 87.9 | <0.001 | 0.008 |  |
| **Fix** | 7 | 0.98 (0.96, 1.00) | 87.9 | < 0.001 | 0.037 |  |
| **Continent** | 7 |  |  |  |  | 0.424 |
| America | 1 | 1.56 (0.68, 3.55) | 0.0 | - | 0.290 |  |
| Europe | 2 | 1.18 (0.70, 1.98) | 46.0 | 0.174 | 0.542 |  |
| Asia | 4 | 0.98 (0.96, 1.00) | 93.5 | < 0.001 | 0.033 |  |
| **Design** | 7 |  |  |  |  | < 0.001 |
| Cohort | 2 | 0.56 (0.47, 0.66) | 0.0 | 0.837 | < 0.001 |  |
| Cross-sectional | 3 | 1.27 (0.87, 1.97) | 8.0 | 0.337 | 0.279 |  |
| Case-control | 2 | 0.99 (0.97, 1.01) | 0.0 | 0.648 | 0.199 |  |
| **Gender** | 7 |  |  |  |  | 0.649 |
| Male | 3 | 0.97 (0.94, 1.01) | 88.5 | < 0.001 | 0.115 |  |
| Female | 4 | 0.98 (0.96, 1.01) | 90.6 | < 0.001 | 0.150 |  |
| **Protein assessment** | 7 |  |  |  |  | 0.424 |
| FFQ | 4 | 0.98 (0.96, 1.00) | 93.5 | < 0.001 | 0.033 |  |
| 24-h dietary recall | 1 | 1.56 (0.68, 3.55) | 0.0 | - | 0.290 |  |
| Others | 2 | 1.18 (0.70, 1.98) | 46.0 | 0.174 | 0.542 |  |
| **Mets assessment** | 7 |  |  |  |  | 0.489 |
| ATP III | 5 | 0.98 (0.96, 1.00) | 91.5 | < 0.001 | 0.035 |  |
| Self-reported | 2 | 1.18 (0.70, 1.98) | 46.0 | 0.174 | 0.542 |  |
| **PA-adjusted** | 7 |  |  |  |  | < 0.001 |
| Yes | 4 | 0.60 (0.51, 0.70) | 67.0 | 0.028 | < 0.001 |  |
| NO | 3 | 0.99 (0.97, 1.01) | 0.0 | 0.497 | 0.209 |  |
| **Smoking-adjusted** | 7 |  |  |  |  | 0.267 |
| Yes | 6 | 0.98 (0.96, 1.00) | 89.7 | < 0.001 | 0.035 |  |
| NO | 1 | 1.56 (0.63, 3.55) | 0.0 | - | 0.290 |  |
| **BMI-adjusted** | 7 |  |  |  |  | 0.238 |
| Yes | 4 | 0.98 (0.96, 1.00) | 93.5 | < 0.001 | 0.033 |  |
| NO | 3 | 1.27 ( 0.82, 1.98) | 8.0 | 0.337 | 0.279 |  |

All studies were adjusted for age

**Supplementary Table 12**: Subgroup analysis based on Fixed-effects models for the association between dietary AP and TG

| **Subgroup** | **Effect sizes**  **(n)** | **Effect sizes**  **(95% CI)** | **I^2^**  **(%)** | **P**  **Heterogeneity** | **P**  **Within** | **P**  **Between** |
| --- | --- | --- | --- | --- | --- | --- |
| **Random** | 8 | 0.92 (0.87, 0.97) | 64.4 | 0.006 | 0.003 |  |
| **Fix** | 8 | 0.92 (0.91, 0.94) | 64.4 | 0.006 | < 0.001 |  |
| **Design** | 8 |  |  |  |  | 0.730 |
| Cohort | 4 | 0.89 (0.79, 1.01) | 37.0 | 0.190 | 0.072 |  |
| Cross-sectional | 2 | 0.82 (0.54, 1.25) | 0.0 | 0.395 | 0.361 |  |
| Case-control | 2 | 0.92 (0.91, 0.94) | 92.6 | <0.001 | < 0.001 |  |
| **Gender** | 8 |  |  |  |  | 0.002 |
| Male | 3 | 0.96 (0.93, 0.98) | 57.6 | 0.094 | 0.001 |  |
| Female | 3 | 0.90 (0.88, 0.92) | 0.0 | 0.369 | < 0.001 |  |
| Both | 2 | 0.82 (0.54, 1.25) | 0.0 | 0.395 | 0.361 |  |
| **Protein assessment** | 8 |  |  |  |  | 0.514 |
| FFQ | 4 | 0.92 (0.91, 0.94) | 83.6 | <0.001 | < 0.001 |  |
| 24-h dietary recall | 4 | 0.87 (0.74, 1.04) | 0.0 | 0.815 | 0.120 |  |
| **PA-adjusted** | 8 |  |  |  |  | 0.481 |
| Yes | 6 | 0.88 (0.78, 1.00) | 11.0 | 0.345 | 0.047 |  |
| NO | 2 | 0.92 (0.91, 0.94) | 92.6 | <0.001 | < 0.001 |  |
| **BMI-adjusted** | 8 |  |  |  |  | 0.514 |
| Yes | 4 | 0.92 (0.91, 0.94) | 83.6 | <0.001 | < 0.001 |  |
| NO | 4 | 0.87 (0.74, 1.04) | 0.0 | 0.815 | 0.120 |  |

All studies were conducted in Asia, used ATP III for evaluating Mets, and adjusted for age and smoking

**Supplementary Table 13**: Subgroup analysis based on Fixed-effects models for the association between dietary AP and HDL

| **Subgroup** | **Effect sizes**  **(n)** | **Effect sizes**  **(95% CI)** | **I^2^**  **(%)** | **P**  **Heterogeneity** | **P**  **Within** | **P**  **Between** |
| --- | --- | --- | --- | --- | --- | --- |
| **Random** | 8 | 0.90 (0.85, 0.95) | 60.0 | 0.014 | < 0.001 |  |
| **Fix** | 8 | 0.90 (0.89, 0.92) | 60.0 | 0.014 | < 0.001 |  |
| **Design** | 8 |  |  |  |  | 0.722 |
| Cohort | 4 | 0.91 (0.81, 1.02) | 76.1 | 0.006 | 0.104 |  |
| Cross-sectional | 2 | 0.76 (0.51, 1.15) | 64.6 | 0.093 | 0.195 |  |
| Case-control | 2 | 0.90 (0.89, 0.92) | 32.4 | 0.224 | < 0.001 |  |
| **Gender** | 8 |  |  |  |  | 0.427 |
| Male | 3 | 0.89 (0.87, 0.92) | 83.8 | 0.002 | < 0.001 |  |
| Female | 3 | 0.91 (0.89, 0.93) | 0.0 | 0.740 | < 0.001 |  |
| Both | 2 | 0.76 (0.51, 1.15) | 64.6 | 0.093 | 0.195 |  |
| **Protein assessment** | 8 |  |  |  |  | 0.222 |
| FFQ | 4 | 0.90 (0.89, 0.92) | 25.6 | 0.258 | < 0.001 |  |
| 24-h dietary recall | 4 | 1.00 (0.85, 1.17) | 75.0 | 0.007 | 0.970 |  |
| **PA-adjusted** | 8 |  |  |  |  | 0.894 |
| Yes | 6 | 0.90 (0.80, 1.00) | 68.8 | 0.007 | 0.055 |  |
| NO | 2 | 0.90 (0.89, 0.92) | 32.4 | 0.224 | < 0.001 |  |
| **BMI-adjusted** | 8 |  |  |  |  | 0.222 |
| Yes | 4 | 0.90 (0.89, 0.92) | 25.6 | 0.258 | < 0.001 |  |
| NO | 4 | 1.00 (0.85, 1.17) | 75.0 | 0.007 | 0.970 |  |

All studies were conducted in Asia, used ATP III for evaluating Mets, and adjusted for age and smoking

**Supplementary Table 14**: Subgroup analysis based on Fixed-effects models for the association between dietary AP and WC

| **Subgroup** | **Effect sizes**  **(n)** | **Effect sizes**  **(95% CI)** | **I^2^**  **(%)** | **P**  **Heterogeneity** | **P**  **Within** | **P**  **Between** |
| --- | --- | --- | --- | --- | --- | --- |
| **Random** | 8 | 0.78 (0.68, 0.90) | 94.0 | < 0.001 | 0.001 |  |
| **Fix** | 8 | 1.00 (0.98, 1.02) | 94.0 | < 0.001 | 0.654 |  |
| **Design** | 8 |  |  |  |  | < 0.001 |
| Cohort | 4 | 0.64 (0.56, 1.02) | 95.0 | < 0.001 | < 0.001 |  |
| Cross-sectional | 2 | 0.62 (0.42, 0.89) | 0.0 | 0.331 | 0.011 |  |
| Case-control | 2 | 1.02 (1.00, 1.04) | 0.0 | 0.363 | 0.103 |  |
| **Gender** | 8 |  |  |  |  | 0.034 |
| Male | 3 | 1.01 (0.98, 1.05) | 97.7 | < 0.001 | 0.492 |  |
| Female | 3 | 1.00 (0.98, 1.03) | 90.4 | < 0.001 | 0.825 |  |
| Both | 2 | 0.62 (0.42, 0.89) | 0.0 | 0.331 | 0.011 |  |
| **Protein assessment** | 8 |  |  |  |  | 0.381 |
| FFQ | 4 | 1.01 (0.99, 1.03) | 97.1 | < 0.001 | 0.585 |  |
| 24-h dietary recall | 4 | 0.93 (0.78, 1.11) | 76.5 | 0.005 | 0.413 |  |
| **PA-adjusted** | 8 |  |  |  |  | < 0.001 |
| Yes | 6 | 0.64 (0.56, 0.72) | 91.8 | < 0.001 | < 0.001 |  |
| NO | 2 | 1.02 (1.00, 1.04) | 0.0 | 0.363 | 0.103 |  |
| **BMI-adjusted** | 8 |  |  |  |  | 0.381 |
| Yes | 4 | 0.59 (0.46, 0.75) | 97.1 | < 0.001 | 0.585 |  |
| NO | 4 | 0.93 (0.78, 1.11) | 76.5 | 0.005 | 0.413 |  |

All studies were conducted in Asia, used ATP III for evaluating Mets, and adjusted for age and smoking

**Supplementary Table 15**: Subgroup analysis based on Fixed-effects models for the association between dietary AP and FBS

| **Subgroup** | **Effect sizes**  **(n)** | **Effect sizes**  **(95% CI)** | **I^2^**  **(%)** | **P**  **Heterogeneity** | **P**  **Within** | **P**  **Between** |
| --- | --- | --- | --- | --- | --- | --- |
| **Random** | 9 | 1.04 (1.00, 1.07) | 29.6 | 0.182 | 0.061 |  |
| **Fix** | 9 | 1.03 (1.01, 1.05) | 29.6 | 0.182 | 0.001 |  |
| **Continent** | 9 |  |  |  |  | 0.046 |
| Asia | 8 | 1.03 (1.01, 1.05) | 5.3 | 0.389 | 0.001 |  |
| America | 1 | 4.26 (1.06, 17.15) | 0.0 | - | 0.041 |  |
| **Design** | 9 |  |  |  |  | 0.942 |
| Cohort | 5 | 10.6 (0.92, 1.21) | 55.1 | 0.063 | 0.425 |  |
| Cross-sectional | 2 | 1.02 (0.73, 1.44) | 0.0 | 0.830 | 0.900 |  |
| Case-control | 2 | 1.03 (1.01, 1.05) | 56.3 | 0.131 | 0.001 |  |
| **Gender** | 9 |  |  |  |  | 0.263 |
| Male | 3 | 1.05 (1.02, 1.08) | 58.5 | 0.090 | 0.001 |  |
| Female | 3 | 1.02 (1.00, 1.04) | 0.0 | 0.987 | 0.113 |  |
| Both | 3 | 1.11 (0.80, 1.55) | 48.1 | 0.146 | 0.543 |  |
| **Protein assessment** | 9 |  |  |  |  | 0.357 |
| FFQ | 5 | 1.03 (1.01, 1.05) | 50.8 | < 0.087 | 0.001 |  |
| 24-h dietary recall | 4 | 1.12 (0.94, 1.32) | 0.0 | 0.496 | 0.195 |  |
| **Mets assessment** | 9 |  |  |  |  | 0.046 |
| ATP III | 8 | 1.03 (1.01, 1.05) | 5.3 | 0.389 | 0.001 |  |
| Biochemical and anthropometric | 1 | 4.26 (1.06, 17.15) | 0.0 | - | 0.041 |  |
| **Age-adjusted** | 9 |  |  |  |  | 0.046 |
| Yes | 8 | 1.03 (1.01, 1.05) | 5.3 | 0.389 | 0.001 |  |
| NO | 1 | 4.26 (1.06, 17.15) | 0.0 | - | 0.041 |  |
| **PA-adjusted** | 9 |  |  |  |  | 0.913 |
| Yes | 6 | 1.04 (0.92, 1.18) | 1.8 | 0.405 | 0.546 |  |
| NO | 3 | 1.03 (1.01, 1.05) | 68.1 | 0.044 | 0.001 |  |
| **Smoking-adjusted** | 9 |  |  |  |  | 0.046 |
| Yes | 8 | 1.03 (1.01, 1.05) | 5.3 | 0.389 | 0.001 |  |
| NO | 1 | 4.26 (1.06, 17.15) | 0.0 | - | 0.041 |  |
| **BMI-adjusted** | 9 |  |  |  |  | 0.357 |
| Yes | 5 | 1.03 (1.01, 1.05) | 50.8 | 0.087 | 0.001 |  |
| NO | 4 | 1.12 (0.94, 1.32) | 0.0 | 0.496 | 0.195 |  |

**Supplementary Table 16**: Subgroup analysis based on Fixed-effects models for the association between dietary AP and BP

| **Subgroup** | **Effect sizes**  **(n)** | **Effect sizes**  **(95% CI)** | **I^2^**  **(%)** | **P**  **Heterogeneity** | **P**  **Within** | **P**  **Between** |
| --- | --- | --- | --- | --- | --- | --- |
| **Random** | 9 | 0.96 (0.95, 0.98) | 0.0 | 0.759 | < 0.001 |  |
| **Fix** | 9 | 0.96 (0.95, 0.98) | 0.0 | 0.759 | < 0.001 |  |
| **Continent** | 9 |  |  |  |  | 0.770 |
| Asia | 8 | 0.06 (0.95, 0.98) | 0.0 | 0.627 | < 0.001 |  |
| America | 1 | 1.03 (0.65, 1.63) | 0.0 | - | 0.899 |  |
| **Design** | 9 |  |  |  |  | 0.209 |
| Cohort | 5 | 0.86 (0.76, 0.98) | 0.0 | 0.839 | 0.021 |  |
| Cross-sectional | 2 | 1.02 (0.73, 1.43) | 0.0 | 0.829 | 0.894 |  |
| Case-control | 2 | 0.96 (0.95, 0.98) | 0.0 | 0.539 | < 0.001 |  |
| **Gender** | 9 |  |  |  |  | 0.752 |
| Male | 3 | 0.97 (0.94, 0.99) | 14.2 | 0.312 | 0.012 |  |
| Female | 3 | 0.96 (0.94, 0.98) | 1.9 | 0.361 | < 0.001 |  |
| Both | 3 | 1.03 (0.78, 1.35) | 0.0 | 0.977 | 0.855 |  |
| **Protein assessment** | 9 |  |  |  |  | 0.535 |
| FFQ | 5 | 0.96 (0.95, 0.98) | 50.8 | 0.448 | < 0.001 |  |
| 24-h dietary recall | 4 | 0.91 (0.77, 1.08) | 0.0 | 0.825 | 0.286 |  |
| **Mets assessment** | 9 |  |  |  |  | 0.770 |
| ATP III | 8 | 0.96 (0.95, 0.98) | 0.0 | 0.672 | < 0.001 |  |
| Biochemical and anthropometric | 1 | 1.03 (0.65, 1.63) | 0.0 | - | 0.899 |  |
| **Age-adjusted** | 9 |  |  |  |  | 0.770 |
| Yes | 8 | 0.96 (0.95, 0.98) | 0.0 | 0.672 | <0.001 |  |
| NO | 1 | 1.03 (0.65, 1.63) | 0.0 | - | 0.899 |  |
| **PA-adjusted** | 9 |  |  |  |  | 0.103 |
| Yes | 6 | 0.87 (0.77, 0.98) | 0.0 | 0.867 | 0.026 |  |
| NO | 3 | 0.96 (0.95, 0.98) | 0.0 | 0.795 | <0.001 |  |
| **Smoking-adjusted** | 9 |  |  |  |  | 0.770 |
| Yes | 8 | 0.96 (0.95, 0.98) | 0.0 | 0.795 | <0.001 |  |
| NO | 1 | 1.03 (0.65, 1.63) | 0.0 | - | 0.899 |  |
| **BMI-adjusted** | 9 |  |  |  |  | 0.535 |
| Yes | 5 | 0.96 (0.95, 0.98) | 0.0 | 0.448 | <0.001 |  |
| NO | 4 | 0.91 (0.77, 1.08) | 0.0 | 0.825 | 0.286 |  |

**Supplementary Table 17**: Subgroup analysis based on Fixed-effects models for the association between dietary PP and TG

| **Subgroup** | **Effect sizes**  **(n)** | **Effect sizes**  **(95% CI)** | **I^2^**  **(%)** | **P**  **Heterogeneity** | **P**  **Within** | **P**  **Between** |
| --- | --- | --- | --- | --- | --- | --- |
| **Random** | 8 | 0.85 (0.74, 0.96) | 89.2 | < 0.001 | 0.012 |  |
| **Fix** | 8 | 1.05 (1.03, 1.08) | 89.2 | < 0.001 | < 0.001 |  |
| **Design** | 8 |  |  |  |  | < 0.001 |
| Cohort | 4 | 0.68 (0.59, 0.78) | 77.9 | 0.004 | < 0.001 |  |
| Cross-sectional | 2 | 0.61 (0.38, 0.98) | 36.8 | 0.209 | 0.042 |  |
| Case-control | 2 | 1.07 (1.04, 1.10) | 6.7 | 0.301 | < 0.001 |  |
| **Gender** | 8 |  |  |  |  | 0.054 |
| Male | 3 | 1.07 (1.03, 1.11) | 92.5 | < 0.001 | 0.001 |  |
| Female | 3 | 1.04 (1.01, 1.08) | 93.4 | < 0.001 | 0.009 |  |
| Both | 2 | 0.61 (0.38, 0.98) | 36.8 | 0.209 | 0.042 |  |
| **Protein assessment** | 8 |  |  |  |  | 0.038 |
| FFQ | 4 | 1.06 (1.03, 1.08) | 94.7 | < 0.001 | < 0.001 |  |
| 24-h dietary recall | 4 | 0.86 (0.71, 1.04) | 25.4 | 0.259 | 0.124 |  |
| **PA-adjusted** | 8 |  |  |  |  | < 0.001 |
| Yes | 6 | 0.67 (0.59, 0.77) | 67.4 | 0.009 | <0.001 |  |
| NO | 2 | 1.07 (1.04, 1.10) | 6.7 | 0.301 | <0.001 |  |
| **BMI-adjusted** | 8 |  |  |  |  | 0.038 |
| Yes | 4 | 1.06 (1.03, 1.08) | 94.7 | < 0.001 | <0.001 |  |
| NO | 4 | 0.86 (0.71, 1.04) | 25.4 | 0.259 | 0.124 |  |

All studies were conducted in Asia, used ATP III for evaluating Mets, and adjusted for age and smoking

**Supplementary Table 18**: Subgroup analysis based on Fixed-effects models for the association between dietary PP and HDL

| **Subgroup** | **Effect sizes**  **(n)** | **Effect sizes**  **(95% CI)** | **I^2^**  **(%)** | **P**  **Heterogeneity** | **P**  **Within** | **P**  **Between** |
| --- | --- | --- | --- | --- | --- | --- |
| **Random** | 8 | 0.83 (0.72, 0.95) | 90.9 | < 0.001 | 0.007 |  |
| **Fix** | 8 | 1.04 (1.01, 1.06) | 90.9 | < 0.001 | 0.002 |  |
| **Design** | 8 |  |  |  |  | < 0.001 |
| Cohort | 4 | 0.70 (0.61, 0.79) | 84.7 | < 0.001 | < 0.001 |  |
| Cross-sectional | 2 | 0.54 (0.35, 0.84) | 88.4 | 0.003 | 0.006 |  |
| Case-control | 2 | 1.05 (1.03, 1.08) | 0.0 | 0.472 | < 0.001 |  |
| **Gender** | 8 |  |  |  |  | 0.006 |
| Male | 3 | 1.02 (0.97, 1.06) | 94.2 | < 0.001 | 0.463 |  |
| Female | 3 | 1.05 (1.02, 1.08) | 91.7 | < 0.001 | 0.001 |  |
| Both | 2 | 0.54 (0.35, 0.84) | 88.4 | 0.003 | 0.006 |  |
| **Protein assessment** | 8 |  |  |  |  | 0.059 |
| FFQ | 4 | 1.04 (1.02, 1.07) | 94.9 | < 0.001 | 0.001 |  |
| 24-h dietary recall | 4 | 0.87 (0.72, 1.05) | 79.2 | 0.002 | 0.139 |  |
| **PA-adjusted** | 8 |  |  |  |  | < 0.001 |
| Yes | 6 | 0.68 (0.60, 0.77) | 83.0 | <0.001 | <0.001 |  |
| NO | 2 | 1.05 (1.03, 1.08) | 0.0 | 0.472 | <0.001 |  |
| **BMI-adjusted** | 8 |  |  |  |  | 0.059 |
| Yes | 4 | 1.04 (1.02, 1.07) | 94.9 | < 0.001 | 0.001 |  |
| NO | 4 | 0.87 (0.72, 1.05) | 79.2 | 0.002 | 0.139 |  |

All studies were conducted in Asia, used ATP III for evaluating Mets, and adjusted for age and smoking

**Supplementary Table 19**: Subgroup analysis based on Fixed-effects models for the association between dietary PP and WC

| **Subgroup** | **Effect sizes**  **(n)** | **Effect sizes**  **(95% CI)** | **I^2^**  **(%)** | **P**  **Heterogeneity** | **P**  **Within** | **P**  **Between** |
| --- | --- | --- | --- | --- | --- | --- |
| **Random** | 8 | 0.34 (0.14, 0.80) | 99.9 | < 0.001 | 0.014 |  |
| **Fix** | 8 | 0.33 (0.33, 0.34) | 99.9 | < 0.001 | < 0.001 |  |
| **Design** | 8 |  |  |  |  | < 0.001 |
| Cohort | 4 | 0.20 (0.20, 0.21) | 84.7 | < 0.001 | < 0.001 |  |
| Cross-sectional | 2 | 0.07 (0.07, 0.07) | 98.1 | < 0.001 | < 0.001 |  |
| Case-control | 2 | 1.02 (0.99, 1.05) | 1.3 | 0.314 | 0.137 |  |
| **Gender** | 8 |  |  |  |  | < 0.001 |
| Male | 3 | 0.46 (0.45, 0.48) | 99.9 | < 0.001 | < 0.001 |  |
| Female | 3 | 0.63 (0.61, 0.64) | 99.9 | < 0.001 | < 0.001 |  |
| Both | 2 | 0.07 (0.07, 0.07) | 98.1 | < 0.001 | < 0.001 |  |
| **Protein assessment** | 8 |  |  |  |  | < 0.001 |
| FFQ | 4 | 0.54 (0.53, 0.55) | 99.9 | < 0.001 | < 0.001 |  |
| 24-h dietary recall | 4 | 0.08 (0.07, 0.08) | 99.5 | < 0.001 | < 0.001 |  |
| **PA-adjusted** | 8 |  |  |  |  | < 0.001 |
| Yes | 6 | 0.13 (0.12, 0.13) | 99.7 | <0.001 | <0.001 |  |
| NO | 2 | 1.02 (1.03, 1.08) | 0.0 | 0.472 | 0.137 |  |
| **BMI-adjusted** | 8 |  |  |  |  | < 0.001 |
| Yes | 4 | 0.54 (0.53, 0.55) | 99.9 | < 0.001 | <0.001 |  |
| NO | 4 | 0.08 (0.07, 0.08) | 99.5 | < 0.001 | <0.001 |  |

All studies were conducted in Asia, used ATP III for evaluating Mets, and adjusted for age and smoking

**Supplementary Table 20**: Subgroup analysis based on Fixed-effects models for the association between dietary PP and FBS

| **Subgroup** | **Effect sizes**  **(n)** | **Effect sizes**  **(95% CI)** | **I^2^**  **(%)** | **P**  **Heterogeneity** | **P**  **Within** | **P**  **Between** |
| --- | --- | --- | --- | --- | --- | --- |
| **Ramdom** | 8 | 0.94 (0.86, 1.03) | 72.2 | 0.001 | 0.207 |  |
| **Fix** | 8 | 1.02 (1.00, 1.05) | 72.2 | 0.001 | 0.085 |  |
| **Design** | 8 |  |  |  |  | 0.001 |
| Cohort | 4 | 0.80 (0.69, 0.92) | 73.3 | 0.011 | 0.002 |  |
| Cross-sectional | 2 | 0.78 (0.51, 1.19) | 88.4 | 0.858 | 0.251 |  |
| Case-control | 2 | 1.03 (1.01, 1.06) | 0.0 | 0.463 | 0.018 |  |
| **Gender** | 8 |  |  |  |  | 0.341 |
| Male | 3 | 1.01 (0.97, 1.05) | 83.2 | 0.003 | 0.553 |  |
| Female | 3 | 1.03 (1.00, 1.07) | 82.0 | 0.004 | 0.064 |  |
| Both | 2 | 0.78 (0.51, 1.19) | 0.0 | 0.858 | 0.251 |  |
| **Protein assessment** | 8 |  |  |  |  | 0.498 |
| FFQ | 4 | 1.02 (1.00, 1.05) | 87.2 | < 0.001 | 0.072 |  |
| 24-h dietary recall | 4 | 0.96 (0.80, 1.15) | 0.0 | 0.742 | 0.663 |  |
| **PA-adjusted** | 8 |  |  |  |  | < 0.001 |
| Yes | 6 | 0.80 (0.69, 0.91) | 55.6 | 0.046 | 0.001 |  |
| NO | 2 | 1.03 (1.01, 1.06) | 0.0 | 0.463 | 0.018 |  |
| **BMI-adjusted** | 8 |  |  |  |  | 0.498 |
| Yes | 4 | 1.02 (1.00, 1.05) | 87.2 | < 0.001 | 0.072 |  |
| NO | 4 | 0.96 (0.80, 1.15) | 0.0 | 0.742 | 0.663 |  |

All studies were conducted in Asia, used ATP III for evaluating Mets, and adjusted for age and smoking

**Supplementary Table 21**: Subgroup analysis based on Fixed-effects models for the association between dietary PP and BP

| **Subgroup** | **Effect sizes**  **(n)** | **Effect sizes**  **(95% CI)** | **I^2^**  **(%)** | **P**  **Heterogeneity** | **P**  **Within** | **P**  **Between** |
| --- | --- | --- | --- | --- | --- | --- |
| **Random** | 8 | 0.91(0.82, 1.01) | 83.1 | <0.001 | 0.090 |  |
| **Fix** | 8 | 1.07 (1.04, 1.09) | 83.1 | <0.001 | <0.001 |  |
| **Design** | 8 |  |  |  |  | <0.001 |
| Cohort | 4 | 0.70 (0.60, 0.80) | 0.0 | 0.468 | < 0.001 |  |
| Cross-sectional | 2 | 0.97 (0.65, 1.45) | 0.0 | 0.627 | 0.876 |  |
| Case-control | 2 | 1.08 (1.05, 1.11) | 70.5 | 0.066 | < 0.001 |  |
| **Gender** | 8 |  |  |  |  | 0.125 |
| Male | 3 | 1.04 (1.00, 1.07) | 86.9 | < 0.001 | 0.068 |  |
| Female | 3 | 1.09 (1.05, 1.12) | 90.8 | < 0.001 | < 0.001 |  |
| Both | 2 | 0.97 (0.65, 1.45) | 0.0 | 0.627 | 0.876 |  |
| **Protein assessment** | 8 |  |  |  |  | 0.004 |
| FFQ | 4 | 1.07 (1.04, 1.10) | 90.4 | < 0.001 | < 0.001 |  |
| 24-h dietary recall | 4 | 0.81 (0.67, 0.98) | 0.0 | 0.569 | 0.029 |  |
| **PA-adjusted** | 8 |  |  |  |  | < 0.001 |
| Yes | 6 | 0.72 (0.63, 0.83) | 0.3 | 0.414 | < 0.001 |  |
| NO | 2 | 1.08 (1.05, 1.11) | 70.5 | 0.066 | < 0.001 |  |
| **BMI-adjusted** | 8 |  |  |  |  | 0.004 |
| Yes | 4 | 1.07 (1.04, 1.10) | 90.4 | < 0.001 | < 0.001 |  |
| NO | 4 | 0.81 (0.67, 0.98) | 0.0 | 0.569 | 0.029 |  |

All studies were conducted in Asia, used ATP III for evaluating Mets, and adjusted for age and smoking
